# Synthesis and cytotoxicity studies of novel *N*-arylbenzo[*h*]quinazolin-2-amines

**DOI:** 10.3762/bjoc.20.218

**Published:** 2024-10-14

**Authors:** Battini Veeraiah, Kishore Ramineni, Dabbugoddu Brahmaiah, Nangunoori Sampath Kumar, Hélène Solhi, Rémy Le Guevel, Chada Raji Reddy, Frédéric Justaud, René Grée

**Affiliations:** 1 Anurag University, Ghatkesar, Hyderabad 500088, TS, India; 2 Chemveda Life Sciences India Pvt. Ltd., #B-11/1, IDA Uppal, Hyderabad-500039, Telangana, India; 3 Univ Rennes, Plateform ImPACcell, BIOSIT, F-35000 Rennes, France; 4 CSIR-Indian Institute of Chemical Technology, Uppal Road, Tarnaka, Hyderabad 500007, TS, India; 5 Univ Rennes, CNRS, ISCR (Institut des Sciences Chimiques de Rennes), UMR 6226, F-35000 Rennes, France

**Keywords:** Buchwald–Hartwig coupling, cytotoxicity, heterocycles, Pd catalysis, quinazolines

## Abstract

In this paper, we report a short and efficient synthesis of novel *N*-arylbenzo[*h*]quinazoline-2-amines. We have prepared a focused library of nineteen representative examples which have been submitted to cytotoxicity assays against a representative panel of eight cancer cell lines and several molecules gave attractive results in this area.

## Introduction

**Figure 1:**
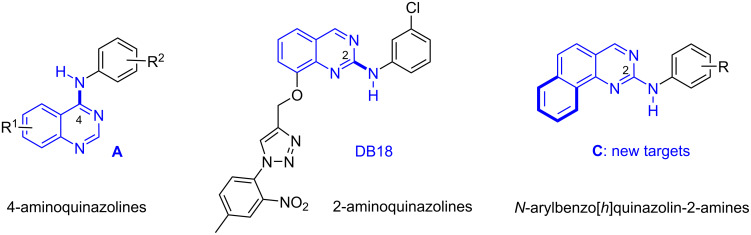
Aminoquinazolines and our new target molecules.

Nitrogen-containing heterocyclic molecules are ubiquitous in living systems. Among them, quinazolines, and especially the 4-aminoquinazolines (type **A** molecules, Figure 1), are recognized as a “privileged scaffold” in bioorganic and medicinal chemistry [1]. On the other hand, the 2-anilinoquinazolines have been less studied but our groups have developed recently the synthesis of new molecules of this family and discovered a compound (DB18) with a potent inhibition (at 10–30 nM) of the CLK1, CLK2 and CLK4 kinases, and a remarkable selectivity since it does not show any action (at 100 μM) against the closely related DYRKs kinases [2–3]. Very recently, this skeleton proved to be also very useful in the research of antimicrobial agents [4], and the closely related dihydropteridinone derivatives were found as potent inhibitors of vaccinia-related kinase 1 and casein kinase 1δ/ε [5]. In addition, it is worth mentioning that closely related compounds like 2-(arylamino)benzo[*h*]quinazolin-4-ones [6–8] and *N*2-arylbenzo[*h*]quinazoline-2,4-diamines [9–11] are well identified scaffolds for bioactive molecules. Thus, it appeared interesting to explore novel molecules with extended aromatic units around this basic quinazoline core and our first choice was *N*-arylbenzo[*h*]quinazoline-2-amines with the general structure **C** as indicated in Figure 1.

Very limited studies have been devoted to the synthesis of this core structure and, to the best of our knowledge, only one molecule of this type was obtained in low yield as a secondary product during studies on the use of the Friedländer annulation reaction starting from naphthylamines [12]. Therefore, the goal of this paper is to describe a short and efficient synthesis for this type of novel skeleton and to prepare a focused library for these targets **C**. Further, we will report results on their cytotoxic activities.

## Results and Discussion

### Chemical synthesis

**Scheme 1:**
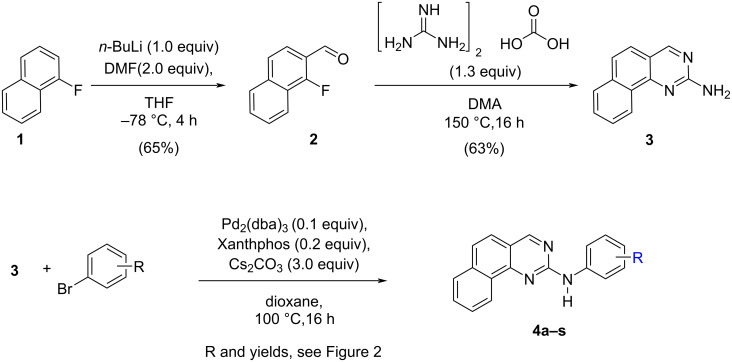
Synthesis of the desired targets **4**.

For the preparation of our targets, we selected a flexible strategy which should allow us to introduce the molecular diversity on the anilino moiety in the last step through a palladium-catalyzed reaction (Scheme 1).

**Figure 2:**
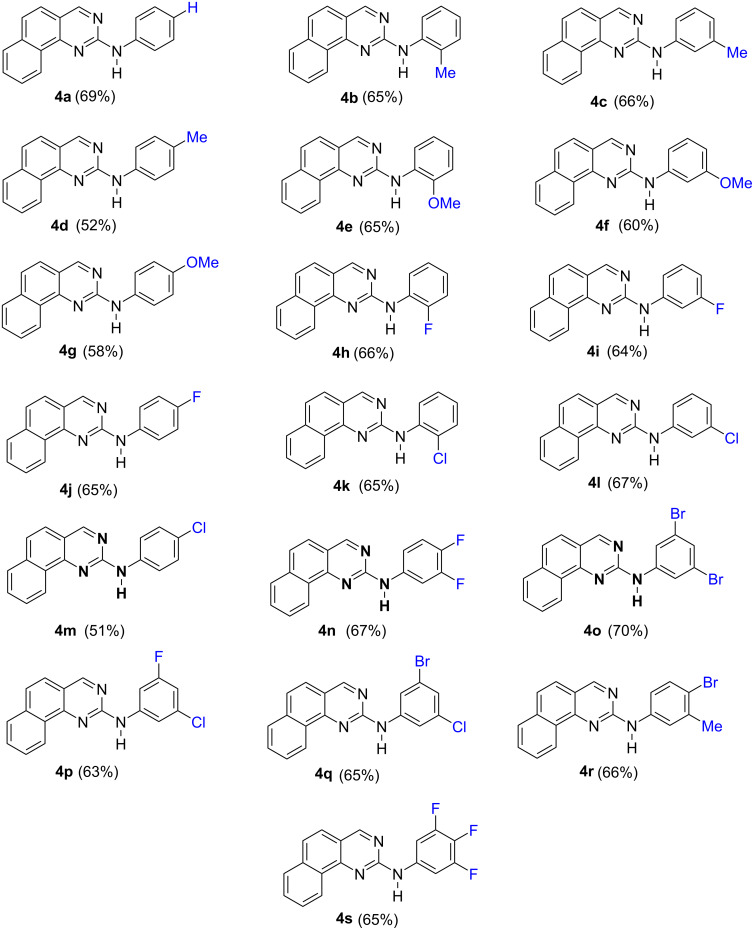
Target molecules **4** prepared with the yields for the last step.

Therefore, we prepared first the core structure **3** in two steps from the commercially available fluoronaphthalene **1**. Metallation, followed by trapping with DMF afforded the known aldehyde **2** in 65% yield [13]. Then, reaction with guanidinium carbonate in DMA at high temperature [12], gave the desired intermediate 2-aminobenzo[*h*]quinazoline (**3**). In a final step, classical Buchwald–Hartwig coupling [14–17] with bromobenzene under the conditions described recently [18], gave the first target **4a** (R = H) in 69% yield. In a similar way, we prepared a designed library with eighteen additional members **4b** to **4s** (Figure 2) having in the different *ortho*, *meta* and *para* positions either methyl groups, halogens (F, Cl) or methoxy groups. We have also prepared compounds with two substituents or with three fluorine atoms. All these compounds have been obtained in fair yields (51–70%) and they have spectral and analytical data in agreement with the indicated structures, as given in the experimental section and Supporting Information File 1.

### Cytotoxicity studies

Next, we performed a cytotoxicity screening of the nineteen molecules **4a–s**, at 25 μM, investigating seven representative cancer cell lines: hepatocellular carcinoma HuH-7, colorectal adenocarcinoma CaCo-2, breast carcinoma MDA-MB-231 and MDA-MB-468, colorectal carcinoma HCT-116, prostate carcinoma PC3 and breast carcinoma MCF7. In parallel, human skin fibroblasts were used as reference for non-tumor cells and roscovitine (ROSCO), doxorubicin (DOXO) and Taxol as positive controls. For details see: ref. [2] and Supporting Information File 1, Table S1.

Compounds **4a–f**, **4h, 4i**, **4k**, **4l**, **4o** and **4q** that induced a more than 30% decrease of cell population for at least one cell line were further investigated.

The determination of IC_50_ confirmed that, although most molecules have low to no activity, a few of them exhibited significant cytotoxicity against several cell lines (Table 1). This is the case in particular for **4a** which has demonstrated low micromolar toxicities (IC_50_ from 1.7 to 6 μM) against HuH-7, Caco-2, MDA-MB-468 and HCT-116 cells, with low to no action, on the other cells. Similarly, the *meta*-fluoro analogue **4i** is active only on Caco-2, MDA-MB-468, HCT-116 and MCF7 (IC_50_ from 2 to 6 μM) while the *meta*-methoxy **4f** and *ortho*-fluoro **4h** show significant toxicity only on Caco-2 (IC_50_ 4.3 and 4.6 μM, respectively). Further, the *para*-methyl compound **4d** acts on HCT-116 only (IC_50_ 5 μM). On the other hand, all molecules exhibited no effect on normal human fibroblasts at the highest concentration tested (25 μM). For these more potent molecules, further studies will be required to rationalize their activities and selectivities and explore their mechanism(s) of action.

**Table 1:** Cytotoxic studies (IC_50_ determination) of selected quinazolines **4**.^a^

cells	HuH-7	Caco-2	MDA-MB-231	MDA-MB-468	HCT-116	PC3	MCF7	Fibro
compounds	IC_50_ µM	IC_50_ µM	IC_50_ µM	IC_50_ µM	IC_50_ µM	IC_50_ µM	IC_50_ µM	IC_50_ µM

DMSO	NE	NE	NE	NE	NE	NE	NE	NE
roscovitine	12	14	15	13	7	10	11	16
doxorubicin	0.03	NE	0.03	0.03	0.04	0.06	0.07	NE
Taxol	0.009	0.07	0.02	0.006	0.003	0.004	0.02	NE
**4a**	**1.7** (±0.4)	**4.7** (±1.3)	29 (±5.5)	**6** (±2.0)	**4** (±0.4)	34.5 (±4.5)	13 (±3.6)	NE
**4b**	NE	NE	NE	NE	NE	NE	NE	NE
**4c**	NE	28 (±7.0)	NE	22 (±3.2)	18 (±1.3)	NE	30 (±3.0)	NE
**4d**	NE	NE	NE	31 (±6.0)	**5** (±1.9)	30 (±8.5)	NE	NE
**4e**	NE	20 (±4.1)	25 (±2.3)	25 (±4.1)	26 (±11)	NE	22 (±4.6)	NE
**4f**	23 (±3.9)	**4.3** (±1.7)	NE	33 (±6.9)	10 (±1.0)	26 (±6.0)	NE	NE
**4h**	NE	**4.6** (±1.6)	NE	NE	NE	NE	NE	NE
**4i**	17 (±3.7)	**2** (±0.6)	17 (±2.1)	**4** (±2.1)	**6** (±0.2)	11 (±0.2)	**3** (±0.8)	NE
**4k**	NE	NE	NE	NE	NE	NE	NE	NE
**4l**	22 (±4.7)	17 (±5.1)	NE	26 (±4.5)	24 (±3.3)	26 (±2.2)	18 (±2.5)	NE
**4o**	24 (±5.1)	16 (±3.5)	NE	20 (±3.0)	23 (±13)	21 (±2.0)	21 (±1.2)	NE
**4q**	16 (±2.3)	14 (±2.9)	20 (±3.0)	19 (±3.1)	13 (±4.4)	19 (±5.2)	14 (±0.6)	NE

^a^IC_50_ determination of effects of quinazolines on seven representative tumor cell lines and normal human fibroblasts. IC_50_ (μM) were calculated from dose-response curves after 48 h exposure (mean of triplicates). NE: no effect.

## Conclusion

In conclusion, we have designed a short and versatile strategy for the preparation of new *N*-arylbenzo[*h*]quinazoline-2-amines and it was exemplified through the preparation of a focused chemical library with 19 members. In addition, the cytotoxicity assays afforded interesting results demonstrating that the substitution on the anilino part can have significant effects on their bioactivity. Two molecules (**4a** and **4i**) exhibited a relatively broad cytotoxicity on four cancer cell lines among the seven assayed. On the other hand, two others (**4f** and **4h**) were active only on Caco-2, while another one (**4d**) only on HCT-116. Thus, the results obtained with these compounds indicate that such a novel heterocyclic scaffold, which has not been used earlier, should offer attractive potentialities in bioorganic and medicinal chemistry. Extension of these studies are ongoing in our laboratories and corresponding results will be reported in due course.

## Experimental

### Chemical synthesis

#### General information

All reactions were performed in heat gun-dried round-bottomed flasks under a dry argon or nitrogen atmosphere. Air and moisture-sensitive compounds were introduced via syringes or cannula, using standard inert atmosphere techniques. In addition, the gas stream was passed through glass cylinder filled with P_2_O_5_ to remove any traces of residual moisture. Reactions were monitored by thin-layer chromatography (TLC) using E. Merck silica gel plates and components were visualized by illumination with short wavelength UV light and/or staining (ninhydrin or basic KMnO_4_). All aldehydes were distilled right before use. All aryl bromides and other reagents were used as they were received from commercial suppliers, unless otherwise noted. THF and Et_2_O were dried over sodium-benzophenone and distilled prior to use.

^1^H NMR spectra were recorded at 300 and 400 MHz, and ^13^C NMR spectra at 75 and 100 MHz, in CDCl_3_ or DMSO-*d*_6_ using TMS (tetramethylsilane) as an internal standard. Multiplicity was tabulated using standard abbreviations: s for singlet, d for doublet, dd for doublet of doublets, t for triplet, q for quadruplet, ddd for doublet of doublets of doublets and m for multiplet (br means broad). When necessary, in particular in order to have better accuracy on small coupling constants, resolution in ^1^H NMR was enhanced using Traficante. All compounds were purified by flash column chromatography on silca gel unless otherwise noted.

Melting points were obtained using an Electrothermal *IA9200* series digital apparatus with thin-wall glass tube used to hold mp samples for capillary mp determination. FTIR spectra were recorded on a Bruker Alpha apparatus with spectra further analysed with spectragryph 1.2.4 version. The absorption bands were reported in cm^−1^. High-resolution mass spectra were recorded in the Centre Régional de Mesures Physiques de l’Ouest, Rennes (CRMPO), on a Maxis 4G.

#### Representative synthesis: preparation of compound **4a**

**Step 1: Synthesis of 1-fluoro-2-naphthaldehyde (2):** To a stirred solution of compound **1** (3.0 g, 0.02 mol) in THF (60 mL) was added *n*-butyllithium (1.6 M, 12.8 mL, 0.02 mol) at −78 °C. The mixture was stirred at −78 °C for 2 h, and then dry DMF (3.3 mL, 0.041 mol) was added. After stirring for 2 h at −78 °C, the mixture was quenched with water and the crude product was extracted with ethyl acetate (30 mL × 3). The combined organic phase was dried over anhydrous Na_2_SO_4_, filtered and concentrated under reduced pressure to afford crude compound **2** as pale-yellow solid (65% yield). Its NMR data are in agreement with literature [5]. ^1^H NMR (400 MHz, DMSO-*d*_6_, δ ppm) 10.49 (s, 1H), 8.26 (d, *J* = 8.4 Hz, 1H), 8.10 (d, *J* = 8.0 Hz, 1H), 7.90 (d, *J* = 8.8 Hz, 1H), 7.84–7.74 (m, 3H).

**Step 2: Synthesis of benzo[*h*]quinazolin-2-amine (3):** To a stirred solution of guanidinium carbonate (2.0 g, 0.011 mol) in DMA (15 mL) was added compound **2** (1.5 g, 0.008 mol). Then the reaction mixture was heated to stir at 150 °C for 16 h. After completion of the reaction (TLC), the mixture was poured into ice water, a solid was formed, filtered and dried to get compound **3** as a brown colored solid (63% yield). ^1^H NMR (400 MHz, DMSO-*d*_6_, δ ppm) 9.06 (s, 1H), 8.91 (d, *J* = 8.0 Hz, 1H), 7.93 (d, *J* = 7.6 Hz, 1H), 7.76–7.64 (m, 3H), 7.56 (d, *J* = 8.8 Hz, 1H), 6.99 (br s, 2H); ^13^C NMR (75 MHz, DMSO-*d*_6_, δ ppm) 162.18, 161.31, 152.03, 136.05, 130.07, 129.07, 128.36, 126.72, 124.51, 124.38, 122.67, 116.44; ^1^H-^13^C NMR ((300, 75) MHz, DMSO-*d*_6_, δ ppm) (9.09 161.23), (8.95 124.31), (7.95 128.17), (7.76 129.93), (7.69 126.77), (7.61 124.66), (7.58 122.55); FTIR (KBr 1%, cm^−1^) ν̃: 3440, 3316, 3192, 1625, 1611, 1598, 1571, 1496, 1471, 1460, 1410, 801, 763; HRESIMS (*m*/*z*): [M + H]^+^ calcd for C_12_H_10_N_3_,196.0869; found, 196.0872 (1 ppm); mp: 193–195 °C (lit. 194–196 °C [12]).

**Step 3: Representative procedure for the preparation of compounds 4:** To the stirred solution of benzo[*h*]quinazolin-2-amine (**3a**, 0.256 mmol, 1.0 equiv) in 1,4-dioxane (4 mL) was added bromobenzene (0.384 mmol, 1.5 equiv), Xanthphos (0.051 mmol, 0.2 equiv) and cesium carbonate (0.769 mmol, 3 equiv). This mixture was degassed for 15 minutes under N_2_ atmosphere. Pd_2_(dba)_3_ (0.0256 mmol, 0.1 equiv) was added and the reaction mixture was stirred for 16 h at 100 °C. After cooling to room temperature, the mixture was filtered through Celite and washed with EtOAc (2 × 10 mL). The filtrate was evaporated under reduced pressure and the crude product was purified by using 60–120 silica mesh column chromatography using 10–20% ethyl acetate in hexane as eluent afforded target compound **4a** (69% yield).

**Compound 4a: *N*-phenylbenzo[*h*]quinazolin-2-amine:** 69% yield (off white solid). ^1^H NMR (400 MHz, DMSO-*d*_6_, δ ppm) 10.01 (br s, 1H, NH), 9.29 (s, 1H), 9.03 (dd, *J* = 7.2, 0.8 Hz, 1H), 8.07–8.01 (m, 3H), 7.84–7.72 (m, 3H), 7.43 (d, *J* = 2.0 Hz, 1H), 7.41 (t, *J* = 7.6 Hz, 1H), 7.05 (t, *J* = 6.8 Hz, 1H); ^13^C NMR (75 MHz, DMSO-*d*_6_, δ ppm) δ 161.2, 158.1, 151.2, 141.0, 136.1, 130.5, 129.2, 129.1, 128.6, 127.4, 124.5, 124.4, 124.3, 122.1, 119.3, 117.6; FTIR (KBr 1%, cm^−1^) ν̃: 3423, 3274, 1643, 1601, 1591, 1542, 1504, 1450, 1393, 1025, 993, 804, 750; HRESIMS (*m*/z): [M + H]^+^ calcd for C_18_H_14_N_3_, 272.1182; found, 272.1181 (0 ppm); mp: 195–197 °C.

### Protocole ImPACell for cytotoxicity studies

**Cell culture.** Skin normal fibroblastic cells are purchased from Lonza (Basel, Switzerland), HuH-7, Caco-2, MDA-MB-231, HCT-116, PC3, MCF7 and NCI-H727 cancer cell lines were obtained from ATCC (American Type Culture Collection). Cells are grown at 37 °C, 5% CO_2_ in ATCC recommended media: DMEM for HuH-7, MDA-MB-231, MDA-MB-468 and fibroblast, EMEM for MCF7 and CaCo-2, McCoy’s for HCT-116 and RPMI for PC3 and NCI-H727. All culture media are supplemented by 10% of FBS, 1% of penicillin-streptomycin and 2 mM glutamine.

### Cytotoxic assays: primary screen (unique concentration) and secondary screen (IC_50_)

Both studies have been performed following the protocols described in a previous paper, see ref. [2].

## Supporting Information

File 1 Supplementary Table S1; spectral and analytical data for compounds **4b**–**s** as well as all copies of ^1^H and ^13^C NMR spectra of compounds **4**.

## Data Availability

All data that supports the findings of this study is available in the published article and/or the supporting information to this article.

## References

[1] Das D, Hong J (2019). Eur J Med Chem.

[2] Brahmaiah D, Kanaka Durga Bhavani A, Aparna P, Sampath Kumar N, Solhi H, Le Guevel R, Baratte B, Ruchaud S, Bach S, Singh Jadav S (2021). Bioorg Med Chem.

[3] Brahmaiah D, Bhavani A K D, Aparna P, Kumar N S, Solhi H, Le Guevel R, Baratte B, Robert T, Ruchaud S, Bach S (2022). Molecules.

[4] Das N, Roy J, Patra B, Saunders E, Sarkar D, Goon S, Sinha B P, Roy S, Roy S, Sarif J (2024). Eur J Med Chem.

[5] de Souza Gama F H, Dutra L A, Hawgood M, Vinícius dos Reis C, Serafim R A M, Ferreira M A, Teodoro B V M, Takarada J E, Santiago A S, Balourdas D-I (2024). J Med Chem.

[6] Alagarsamy V, Chitra K, Saravanan G, Solomon V R, Sulthana M T, Narendhar B (2018). Eur J Med Chem.

[7] Collet J W, van der Nol E A, Roose T R, Maes B U W, Ruijter E, Orru R V A (2020). J Org Chem.

[8] Dong Y, Zhang J, Yang J, Yan C, Wu Y (2021). New J Chem.

[9] Sengupta S K, Chatterjee S, Protopapa H K, Modest E J (1972). J Org Chem.

[10] Rosowsky A, Papathanasopoulos N (1974). J Org Chem.

[11] Khan I, Ibrar A, Abbas N, Saeed A (2014). Eur J Med Chem.

[12] Malinowski Z, Fornal E, Warpas A, Nowak M (2018). Monatsh Chem.

[13] Leroux F, Mangano G, Schlosser M (2005). Eur J Org Chem.

[14] Paul F, Patt J, Hartwig J F (1994). J Am Chem Soc.

[15] Guram A S, Buchwald S L (1994). J Am Chem Soc.

[16] Guram A S, Rennels R A, Buchwald S L (1995). Angew Chem, Int Ed Engl.

[17] Louie J, Hartwig J F (1995). Tetrahedron Lett.

[18] Xiang J, Wang Y, Wang W, Yu J, Zheng L, Hong Y, Shi L, Zhang C, Chen N, Xu J (2023). Bioorg Chem.

