# Supplementary material for: Synthesis and cytotoxicity studies of novel N-arylbenzo[h]quinazolin-2-amines
Source: Beilstein J Org Chem. 2024 Oct 14;20:2592–8. doi: 10.3762/bjoc.20.218 (PMC11496704; doi:10.3762/bjoc.20.218)
Supplement: File 1 — Supplementary Table S1; spectral and analytical data for compounds 4b–s as well as all copies of 1H and 13C NMR spectra of compounds 4. [file Beilstein_J_Org_Chem-20-2592-s001.pdf]

BEILSTEIN JOURNAL OF ORGANIC CHEMISTRY

Supporting Information

for

Synthesis and cytotoxicity studies of novel *N*-arylbenzo[*h*]quinazolin-2-amines

Battini Veeraiah, Kishore Ramineni, Dabbugoddu Brahmaiah, Nangunoori Sampath Kumar, Hélène Solhi, Rémy Le Guevel, Chada Raji Reddy, Frédéric Justaud and René Grée

Beilstein J. Org. Chem. doi:

Supplementary Table S1; spectral and analytical data for compounds 4b–s as well as all copies of ^{1}H and ^{13}C NMR spectra of compounds 4

License and Terms: This is a supporting information file under the terms of the Creative Commons Attribution License (https://creativecommons.org/licenses/by/4.0). Please note that the reuse, redistribution and reproduction in particular requires that the author(s) and source are credited and that individual graphics may be subject to special legal provisions.
The license is subject to the *Beilstein Journal of Organic Chemistry* terms and conditions: (https://www.beilstein-journals.org/bjoc/terms)

Table S1: Cytotoxicity studies (single dose at 25μM) of *N*-arylbenzo[*h*]quinazolin-2-amines 4a–s.................................S3
Spectral and analytical data for compounds **4b**–**s**. S4
Compound **4b**: *N*-(*o*-tolyl)benzo[*h*]quinazolin-2-amine: S4
Compound **4c**: *N*-(*m*-tolyl)benzo[*h*]quinazolin-2-amine..........S4
Compound **4d**: *N*-(*p*-tolyl)benzo[*h*]quinazolin-2-amine S4
Compound **4e**: *N*-(2-methoxyphenyl)benzo[*h*]quinazolin-2-amine S4
Compound **4f**: *N*-(3-methoxyphenyl)benzo[*h*]quinazolin-2-amine..........S4
Compound **4g**: *N*-(4-methoxyphenyl)benzo[*h*]quinazolin-2-amine S5
Compound **4h**: *N*-(2-fluorophenyl)benzo[*h*]quinazolin-2-amine S5
Compound **4i**: *N*-(3-fluorophenyl)benzo[*h*]quinazolin-2-amine S5
Compound **4j**: *N*-(4-fluorophenyl)benzo[*h*]quinazolin-2-amine S5
Compound **4k**: *N*-(2-chlorophenyl)benzo[*h*]quinazolin-2-amine..........S5
Compound **4l**: *N*-(3-chlorophenyl)benzo[*h*]quinazolin-2-amine..........S6
Compound **4m**: *N*-(4-chlorophenyl)benzo[*h*]quinazolin-2-amine..........S6
Compound **4n**: *N*-(3,4-difluorophenyl)benzo[*h*]quinazolin-2-amine S6
Compound **4o**: *N*-(3,5-dibromophenyl)benzo[*h*]quinazolin-2-amine S6
Compound **4p**: *N*-(3-chloro-5-fluorophenyl)benzo[*h*]quinazolin-2-amine S6
Compound **4q**: *N*-(3-bromo-5-chlorophenyl)benzo[*h*]quinazolin-2-amine S7
Compound **4r**: *N*-(4-bromo-3-methylphenyl)benzo[*h*]quinazolin-2-amine..........S7
Compound **4s**: *N*-(3,4,5-trifluorophenyl)benzo[*h*]quinazolin-2-amine S7
Spectra: 1H, 13C, 13C Jmod NMR and FT-IR data of compound **3** S8
Spectra: ^{1}H, ^{13}C, ^{13}C Jmod NMR and FT-IR data of compound **4a** (DV-02)..........S10
Spectra: ^{1}H, ^{13}C, ^{13}C Jmod NMR and FT-IR data of compound **4b** (DV-03)..........S13
Spectra: ^{1}H, ^{13}C, ^{13}C Jmod NMR and FT-IR data of compound **4c** (DV-04) S16
Spectra: ^{1}H, ^{13}C, ^{13}C Jmod NMR and FT-IR data of compound **4d** (DV-05)..........S19
Spectra: ^{1}H, ^{13}C, ^{13}C Jmod NMR and FT-IR data of compound **4e** (DV-06)..........S21
Spectra: ^{1}H, ^{13}C, ^{13}C Jmod NMR and FT-IR data of compound **4f** (DV-07) S23
Spectra: ^{1}H, ^{13}C, ^{13}C Jmod NMR and FT-IR data of compound **4g** (DV-08)..........S26
Spectra: ^{1}H, ^{13}C, ^{13}C Jmod NMR and FT-IR data of compound **4h** (DV-09)..........S28
Spectra: ^{1}H, ^{13}C, ^{13}C Jmod NMR and FT-IR data of compound **4i** (DV-10)..........S31
Spectra: ^{1}H, ^{13}C, ^{13}C Jmod NMR and FT-IR data of compound **4j** (DV-11)..........S34
Spectra: ^{1}H, ^{13}C, ^{13}C Jmod NMR and FT-IR data of compound **4k** (DV-12)..........S37
Spectra: ^{1}H, ^{13}C, ^{13}C Jmod NMR and FT-IR data of compound **4l** (DV-13)..........S39
Spectra: ^{1}H, ^{13}C, ^{13}C Jmod NMR and FT-IR data of compound **4m** (DV-14)..........S41
Spectra: ^{1}H, ^{13}C, ^{13}C Jmod NMR and FT-IR data of compound **4n** (DV-15)..........S43
Spectra: ^{1}H, ^{13}C, ^{13}C Jmod NMR and FT-IR data of compound **4o** (DV-16)..........S46
Spectra: ^{1}H, ^{13}C, ^{13}C Jmod NMR and FT-IR data of compound **4p** (DV-17)..........S48

Spectra: ^{1}H, ^{13}C, ^{13}C Jmod NMR and FT-IR data of compound **4q** (DV-18)......S51
Spectra: ^{1}H, ^{13}C, ^{13}C Jmod NMR and FT-IR data of compound **4s** (DV-20)......S53
Spectra: ^{1}H, ^{13}C, ^{13}C Jmod NMR and FT-IR data of compound **4r** (DV-19)......S55

Table S1: Cytotoxicity studies (single dose at 25µM)

of *N*-arylbenzo[*h*]quinazolin-2-amines 4a–s

N°	HuH7	CaCo-2	MDA231	HCT116	PC3	MDA468	MCF7
DMSO	109	78	97	88	86	92	97
ROSCO	30	11	18	8	19	9	22
DOXO	59	59	31	14	42	24	38
TAXOL	43	36	32	7	30	18	27
4a	60	33	61	48	58	56	59
4b	69	56	86	79	72	93	70
4c	68	50	81	52	60	65	50
4d	85	63	67	54	65	72	61
4e	55	59	40	8	21	46	82
4f	59	59	77	29	54	73	78
4g	79	81	87	72	82	90	83
4h	81	48	83	82	74	94	83
4i	60	34	49	11	36	59	59
4j	92	77	91	81	78	95	81
4k	76	61	91	83	73	93	56
4l	71	38	67	56	59	78	68
4m	96	75	83	68	74	88	88
4n	79	72	91	82	82	85	97
4o	62	46	67	27	38	47	85
4p	80	54	68	17	59	72	91
4q	49	35	41	13	24	38	73
4r	80	76	101	100	89	96	85
4s	77	65	78	63	50	78	54

Effects of quinazolines **4** on seven representative tumor cell lines. The toxicity is expressed as the percentage of cell survival (from 100 % to 0 %) over DMSO control after 48 h exposure (mean of triplicates). Roscovitine (ROSCO) at 25 µM, Doxorubicine (DOXO) and Taxol (TAXOL) both at 0.025 µM were used as reference compounds. Effect of the solvent (DMSO) was studied at 0.25%.

Spectral and analytical data for compounds 4b–s.

Compound 4b: *N*-(*o*-tolyl)benzo[*h*]quinazolin-2-amine

65% Yield (Light brown solid) **4b**: **^{1}H NMR (400 MHz, DMSO-d6, δ ppm):** 9.21 (br s, 1H, NH), 9.13 (s, 1H), 8.78 (d, J = 8.4 Hz, 1H), 7.97 (d, J = 7.6 Hz, 1H), 7.82-7.72 (m, 3H), 7.69-7.65 (m, 2H), 7.34-7.26 (m, 2H), 7.13-7.10 (m, 1H), 2.31 (s, 3H). **^{13}C NMR (75 MHz, DMSO-d6, δ ppm):** 161.3, 159.1, 151.4, 138.5, 136.1, 132.3, 130.8, 130.3, 129.2, 128.5, 127.1, 126.4, 124.8, 124.7, 124.4 (2 x C), 123.8, 117.4, 18.7. **FTIR (KBr 1%, cm−1): $\tilde{\nu}$ =** 3225, 3025, 2962, 2922, 1611, 1584, 1530, 1488, 1450, 1389, 826, 799, 763, 747, 724. **HRMS m/z (ESI):** calcd for $C_{19}H_{16}N_3$ $(M + H)^+$ 286.1338, found 286.1342 (1 ppm). **Mp:** 126-128 °C.

Compound 4c: *N*-(*m*-tolyl)benzo[*h*]quinazolin-2-amine

66% Yield (White solid) **4c**: **^{1}H NMR (400 MHz, DMSO-d6, δ ppm):** 9.96 (br s, 1H, NH), 9.29 (s, 1H), 8.99 (dd, J = 7.6, 2.0 Hz, 1H), 8.02 (dd, J = 6.8, 2.0 Hz, 1H), 7.90-7.79 (m, 5H), 7.78 (d, J = 5.6 Hz, 1H), 7.29 (t, J = 7.6 Hz, 1H), 6.85 (t, J = 7.6 Hz, 1H), 2.38 (s, 3H). **^{13}C NMR (75 MHz, DMSO-d6, δ ppm):** 161.2, 158.1, 151.2, 141.0, 138.1, 136.1, 130.4, 129.2, 129.0, 128.6, 127.4, 124.5, 124.4, 124.3, 122.8, 119.9, 117.6, 116.5, 22.0. **FTIR (KBr 1%, cm−1): $\tilde{\nu}$ =** 3281, 3212, 3139, 3111, 3052, 1609, 1585, 1544, 1506, 1452, 1395, 794, 778, 760. **HRMS m/z (ESI):** calcd for $C_{19}H_{16}N_3$ $(M + H)^+$ 286.1338, found 286.1343 (2 ppm). **Mp:** 161-163 °C.

Compound 4d: *N*-(*p*-tolyl)benzo[*h*]quinazolin-2-amine

52% Yield (Off white solid) **4d**: **^{1}H NMR (400 MHz, DMSO-d6, δ ppm):** 9.90 (br s, 1H, NH), 9.26 (s, 1H), 8.98 (dd, J = 7.2, 1.6 Hz, 1H), 8.00 (dd, J = 8.8, 1.2 Hz, 1H), 7.92 (d, J = 8.4 Hz, 2H), 7.83-7.75 (m, 4H), 7.22 (d, J = 8.4 Hz, 2H), 2.31 (s, 3H). **^{13}C NMR (75 MHz, DMSO-d6, δ ppm):** 161.2, 158.2, 151.2, 138.5, 136.1, 130.9, 130.4, 129.5, 129.2, 128.6, 127.3, 124.5, 124.4, 124.1, 119.4, 117.5, 20.9. **FTIR (KBr 1%, cm−1): $\tilde{\nu}$ =** 3450 (Br), 3115, 3050, 2256, 2130, 1650, 1618, 1607, 1593, 1547, 1511, 1450, 1412, 1391, 1049, 1021, 994, 822, 801, 763. **HRMS m/z (ESI):** calcd for $C_{19}H_{16}N_3$ $(M + H)^+$ 286.1338, found 286.1339 (0 ppm). **Mp:** 137-139°C.

SI Compound 4e: *N*-(2-methoxyphenyl)benzo[*h*]quinazolin-2-amine

65% Yield (Pale yellow solid) **4e**: **^{1}H NMR (400 MHz, DMSO-d6, δ ppm):** 9.28 (br s, 1H, NH), 8.98 (dd, J = 7.2, 0.8 Hz, 1H), 8.60 (dd, J = 7.6, 2.0 Hz, 1H), 8.40 (s, 1H), 8.02 (dd, J = 8.8, 1.2 Hz, 1H), 7.84-7.73 (m, 4H), 7.14-7.08 (m, 3H), 3.91 (s, 3H). **^{13}C NMR (75 MHz DMSO-d6, δ ppm):** 161.3, 157.9, 151.3, 149.4, 136.1, 130.5, 129.1, 129.1, 128.6, 127.4, 124.5, 124.3, 123.2, 121.1, 120.0, 117.8, 111.4, 56.3. **FTIR (KBr 1%, cm−1): $\tilde{\nu}$ =** 3430, 3400, 3048, 2972, 2944, 2841, 1584, 1531, 1482, 1453, 1427, 1383, 1245, 1020, 799, 760, 734. **HRMS m/z (ESI):** calcd for $C_{19}H_{16}N_3O$ $(M + H)^+$ 302.1287, found 302.1291 (1 ppm). **Mp:** 138-140°C.

Compound 4f: *N*-(3-methoxyphenyl)benzo[*h*]quinazolin-2-amine

60% Yield (Yellow solid) **4f**: **^{1}H NMR (400 MHz, DMSO-d6, δ ppm):** 10.02 (br s, 1H, NH), 9.30 (s, 1H), 9.02 (d, J = 7.6 Hz, 1H), 8.03 (d, J = 7.6 Hz, 1H), 7.87 (s, 1H), 7.84-7.73 (m, 4H), 7.57 (d, J = 8.0 Hz, 1H), 7.30 (t, J = 8.4 Hz, 1H), 6.62 (dd, J = 8.0, 2.0 Hz, 1H), 3.85 (s, 3H). **^{13}C NMR (75 MHz, DMSO-d6, δ ppm):** δ 161.2, 160.1, 158.0, 151.1, 142.3, 136.1, 130.5, 129.8, 129.2, 128.7, 127.3, 124.4, 124.4, 117.7, 111.7, 107.6, 104.9, 55.5. **FTIR (KBr 1%, cm−1): $\tilde{\nu}$ =** 3267, 3122, 3097, 3046, 2991,

2962, 2928, 2833, 1610, 1585, 1568, 1534, 1511, 1461, 1431, 1389, 1286, 1261, 1155, 861, 802, 764, 722. **HRMS m/z (ESI):** calcd for $C_{19}H_{16}N_3O$ $(M + H)^+$ 302.1287, found 302.1292 (1 ppm). **Mp:** 171-173°C.

Compound 4g: *N*-(4-methoxyphenyl)benzo[*h*]quinazolin-2-amine

58% Yield (Yellow solid) **4g**: **^{1}H NMR (400 MHz, DMSO-d6, δ ppm):** 9.83 (br s, 1H, NH), 9.24 (s, 1H), 8.98 (dd, J = 8.0, 1.6 Hz, 1H), 8.01 (dd, J = 8.0, 1.2 Hz, 1H), 7.94 (d, J = 8.8 Hz, 2H), 7.82-7.74 (m, 4H), 7.69 (d, J = 8.4 Hz, 1H), 7.00 (d, J = 7.2 Hz, 1H), 3.77 (s, 3H). **^{13}C NMR (101 MHz, DMSO-d6, δ ppm):** 161.2, 158.3, 154.8, 151.3, 136.2, 134.2, 130.4, 129.2, 128.6, 127.2, 124.6, 124.4, 123.9, 120.9, 117.4, 114.4, 55.7. **FTIR (KBr 1%, cm−1): $\tilde{\nu}$ =** 3426, 3248, 3046, 2964, 2920, 1610, 1588, 1544, 1504, 1452, 1414, 1389, 1256, 1231, 1099, 1026, 799, 766. **HRMS m/z (ESI):** calcd for $C_{19}H_{16}N_3O$ $(M + H)^+$ 302.1287, found 302.1290 (1 ppm). **Mp:** 186-188°C.

Compound 4h: *N*-(2-fluorophenyl)benzo[*h*]quinazolin-2-amine

66% Yield (Off white solid) **4h**: **1H NMR (400 MHz, DMSO-d6, δ ppm):** 9.44 (br s, 1H, NH), 9.27 (s, 1H), 8.87 (d, J = 8.0 Hz, 1H), 8.11 (t, J = 6.8 Hz, 1H), 8.00 (d, J = 8.0 Hz, 1H), 7.81-7.70 (m, 4H), 7.34-7.27 (m, 2H), 7.22-7.18 (m, 1H). **^{13}C NMR (101 MHz, DMSO-d6, δ ppm):** 161.3, 158.4, 155.3 (d, J_{CF} = 246.5 Hz), 151.3, 136.1, 130.5, 129.11, 128.5, 128.1 (d, J_{CF} = 11.3 Hz), 127.3, 125.1, 125.02 (d, J_{CF} = 5.6 Hz), 124.7 (d, J_{CF} = 3.8 Hz), 124.4 (d, J_{CF} = 3.1 Hz), 124.3, 117.9, 116.0 (d, J_{CF} = 19.7 Hz. **^{19}F NMR (282 MHz, DMSO, δ ppm):** -122.30 – -122.49 (m). FTIR (KBr 1%, cm−1): $\tilde{\nu}$ =3245, 3051, 3011, 1603, 1590, 1546, 1496, 1451, 1401, 1251, 1197, 804, 765, 739. **HRMS m/z (ESI):** calcd for $C_{18}H_{13}N_3F$ $(M + H)^+$ 290.1088, found 290.1087 (0 ppm). **Mp:** 161-163°C.

SI Compound 4i: *N*-(3-fluorophenyl)benzo[*h*]quinazolin-2-amine

64% Yield (Pale yellow solid) **4i**: **^{1}H NMR (400 MHz, DMSO-d6, δ ppm):** 10.27 (br s, 1H, NH), 9.34 (s, 1H), 8.99 (dd, J = 7.2, 3.2 Hz, 1H), 8.13-8.04 (m, 2H), 7.86-7.77 (m, 5H), 7.43 (q, J = 8.4 Hz, 1H), 6.86-6.84 (m, 1H). **^{13}C NMR (75 MHz, DMSO-d6, δ ppm):** 162.9 (d, J_{CF} = 240.4 Hz), 161.3, 157.8, 151.0, 143.0 (d, J_{CF} = 11.5 Hz), 136.1, 130.7, 130.6, 129.1, 128.7, 127.5, 124.8, 124.3, 124.3, 117.9, 115.0 (d, J_{CF} = 2.5 Hz), 108.2 (d, J_{CF} = 21.3 Hz), 105.6 (d, J_{CF} = 26.7 Hz). **FTIR (KBr 1%, cm−1): $\tilde{\nu}$ =** 3425, 3281, 1593, 1537, 1510, 1491, 1448, 1391, 1256, 1135, 1050, 1020, 1000, 853, 792, 760. ^{19}F NMR (282 MHz, **DMSO-d6, δ ppm):** -112.23 (dt, *J* = 12.8, 8.0 Hz). **HRMS m/z (ESI):** calcd for $C_{18}H_{13}N_3F$ $(M + H)^+$ 290.1088, found 290.1085 (1 ppm) (0 ppm). **Mp:** 187-189°C.

Compound 4j: *N*-(4-fluorophenyl)benzo[*h*]quinazolin-2-amine

65% Yield (Off white solid) **4j**: **^{1}H NMR (400 MHz, DMSO-d6, δ ppm):** 10.05 (br s, 1H, NH), 9.29 (s, 1H), 8.99 (dd, J = 8.0, 1.6 Hz, 1H), 8.07-8.01 (m, 3H), 7.84-7.72 (m, 4H), 7.24 (t, J = 8.8 Hz, 2H). **^{13}C NMR (75 MHz, DMSO-d6, δ ppm):** 161.3, 158.7 (d, J_{CF} = 238.9 Hz), 151.2, 137.4, 137.4, 136.1, 130.5, 129.1, 128.6, 127.3, 124.6, 124.3, 124.3, 120.9 (d, J_{CF} = 7.6 Hz), 117.7, 115.6 (d, J_{CF} = 22.2 Hz). **^{19}F NMR (282 MHz, DMSO-d6, δ ppm):** -121.55 (tt, *J* = 5.1, 8.7 Hz). **FTIR (KBr 1%, cm−1): $\tilde{\nu}$ =** 3420, 3258, 3058, 1606, 1584, 1551, 1510, 1450, 1413, 1390, 1205, 1026, 995, 835, 800, 761. **HRMS m/z (ESI):** calcd for $C_{18}H_{13}N_3F$ $(M + H)^+$ 290.1088, found 290.1089 (0 ppm). **Mp:** 233-235°C.

Compound 4k: *N*-(2-chlorophenyl)benzo[*h*]quinazolin-2-amine

65% Yield (Pale yellow solid) **4k**: **^{1}H NMR (400 MHz, DMSO-d6, δ ppm):** 9.28 (br s, 1H, NH), 9.09 (s, 1H), 8.86 (d, J = 8.0 Hz, 1H), 8.20 (dd, J = 8.4, 1.6 Hz, 1H), 8.01 (d, J = 8.0 Hz, 1H), 7.82-7.70 (m, 4H), 7.57 (dd, J = 8.0, 1.2 Hz, 1H), 7.47 (t, J = 8.4

Hz, 1H), 7.22 (t, J = 8.0 Hz, 1H). **^{13}C NMR (101 MHz, DMSO-d6, δ ppm):** 161.4, 158.3, 151.2, 137.0, 136.1, 130.5, 130.0, 129.1, 128.5, 128.0, 127.3, 126.8, 125.5, 125.2, 124.6, 124.5, 124.3, 118.0. **FTIR (KBr 1%, cm−1): $\tilde{\nu}$ =** 3420, 3103, 3063, 1582, 1523, 1443, 1385, 1296, 1049, 1028, 796, 761, 744. **HRMS m/z (ESI):** calcd for $C_{18}H_{13}N_3{}^{35}Cl$ $(M + H)^+$ 306.07925, found 306.0792 (0 ppm). **Mp:** 120-122°C.

Compound 4l: *N*-(3-chlorophenyl)benzo[*h*]quinazolin-2-amine

67% Yield (Pale yellow solid) **4l**: **^{1}H NMR (400 MHz, DMSO-d6, δ ppm):** 10.26 (br s, 1H, NH), 9.35 (s, 1H), 8.99 (dd, J = 7.6,1.6 Hz, 1H), 8.33 (t, J = 2.0 Hz, 1H), 8.04(dd, J = 8.8,1.6 Hz 1H), 7.98 (dd, J = 8.4, 1.2 Hz, 1H), 7.85-7.75 (m, 4H), 7.44 (t, J = 8.0 Hz, 1H), 7.08 (dd, J = 7.2, 1.6 Hz, 1H). **^{13}C NMR (101 MHz, DMSO-d6, δ ppm):** 161.4, 157.8, 151.0, 142.6, 136.2, 133.5, 130.8, 130.6, 129.1, 128.7, 127.5, 124.8, 124.3, 124.3, 121.5, 118.5, 118.0, 117.5. **FTIR (KBr 1%, cm−1): $\tilde{\nu}$ =** 3290, 3112, 3048, 1584, 1533, 1480, 1446, 1410, 1395, 800, 764. **HRMS m/z (ESI):** calcd for $C_{18}H_{13}N_3{}^{35}Cl$ $(M + H)^+$ 306.0792, found 306.0792 (0 ppm). **Mp:** 207-209°C.

Compound 4m: *N*-(4-chlorophenyl)benzo[*h*]quinazolin-2-amine

51% Yield (Off white solid) **4m**: **^{1}H NMR (400 MHz, DMSO-d6, δ ppm):** 10.17 (br s, 1H, NH), 9.31 (s, 1H), 9.01 (d, J = 7.6 Hz, 1H), 8.09 (d, J = 8.8 Hz, 2H), 8.07 (d, J = 3.2 Hz, 1H), 7.85-7.72 (m, 4H), 7.48 (d, J = 3.2 Hz, 2H). **^{13}C NMR (101 MHz, DMSO-d6, δ ppm):** 161.3, 157.9, 151.1, 140.0, 136.1, 130.5, 129.1, 129.0, 128.6, 127.5, 125.5, 124.6, 124.3, 120.7, 119.3, 117.8. **FTIR (KBr 1%, cm−1): $\tilde{\nu}$ =** 3426, 3256, 3099, 3046, 2924, 2851, 1605, 1582, 1544, 1490, 1452, 1400, 801, 755. **HRMS m/z (ESI):** calcd for $C_{18}H_{13}N_3{}^{35}Cl$ $(M + H)^+$ 306.0792, found 306.0792 (0 ppm). **Mp:** decomposition >200°C.

SI Compound 4n: *N*-(3,4-difluorophenyl)benzo[*h*]quinazolin-2-amine

67% Yield (Light brown solid) **4n**: **^{1}H NMR (400 MHz, DMSO-d6, δ ppm):** 10.26 (br s, 1H, NH), 9.33 (s, 1H), 8.97 (dd, J = 7.2, 2.0 Hz, 1H), 8.29-8.23 (m, 1H), 8.05 (dd, J = 6.8, 2.0 Hz, 1H), 7.86-7.75 (m, 5H), 7.45 (q, J = 9.6 Hz, 1H). **^{13}C NMR (101 MHz, DMSO-d6, δ ppm):** 161.4, 159.6 (dd, J_{CF} = 13.2 and 242.0 Hz), 144.6 (dd, J_{CF} = 13.1 and 240 Hz), 143. 6, 143.43, 138.2 (dd, J_{CF} = 2.9 and 9.6 Hz), 136.1, 130.6, 129.0, 128.7, 127.5, 124.8, 124.3, 124.3, 118.0, 117.8 (d, J_{CF} = 17.7 Hz), 115.3 (dd, J_{CF} = 2.3 and 5.2 Hz), 107.7 (d, J_{CF} = 21.8 Hz). **FTIR (KBr 1%, cm−1): $\tilde{\nu}$ =** 3422, 3283, 1612, 1588, 1544, 1509, 1450, 1387, 1271, 1198, 794, 760. **HRMS m/z (ESI):** calcd for $C_{18}H_{12}N_3F_2$ $(M + H)^+$ 308.09938, found 308.0995 (0 ppm). **Mp:** 217-218°C.

Compound 4o: *N*-(3,5-dibromophenyl)benzo[*h*]quinazolin-2-amine

70% Yield (Light brown solid) **4o**: **^{1}H NMR (400 MHz, DMSO-d6, δ ppm):** 10.43 (br s, 1H, NH), 9.39 (s, 1H), 8.96 (d, J = 8.0 Hz, 1H), 8.40 (d, J = 2.0 Hz, 2H), 8.07 (d, J = 7.2 Hz, 1H), 8.07 (d, J = 7.6 Hz, 1H), 7.87-7.78 (m, 4H), 7.44 (t, J = 1.6 Hz, 1H). **^{13}C NMR (75 MHz, DMSO-d6, δ ppm):** 161.5, 157.4, 150.8, 143.9, 136.2, 130.7, 129.0, 128.9, 127.5, 126.0, 125.3, 124.3, 124.1, 122.8, 120.2, 118.3. **FTIR (KBr 1%, cm−1): $\tilde{\nu}$ =** 3437, 3290, 2964, 1584, 1528, 1510, 1448, 1402, 1259, 1095, 1021, 862, 796. **HRMS m/z (ESI):** calcd for $C_{18}H_{12}N_3{}^{79}Br_2$ $(M + H)^+$ 427.9392, found 427.9393 (0 ppm). **Mp:** 234-236°C.

Compound 4p: *N*-(3-chloro-5-fluorophenyl)benzo[*h*]quinazolin-2-amine

63% Yield (Off white solid) **4p**: **^{1}H NMR (400 MHz, DMSO-d6, δ ppm):** 10.47 (br s, 1H, NH), 9.38 (s, 1H), 8.95 (d, J = 7.2 Hz, 1H), 8.08-8.00 (m, 3H), 7.87-7.80 (m, 4H), 7.03 (dd, J = 6.4, 2.0 Hz, 1H). **^{13}C NMR (75 MHz, DMSO-d6, δ ppm):** 162.8 (d, J_{CF} = 243 Hz), 161.3, 157.43 150.8, 143.7 (d, J_{CF} = 13 Hz), 136.1, 134.2 (d, J_{CF} = 13.5 Hz), 130.6, 129.0, 128.75 127.5, 125.2, 124.2, 124.1, 118.2, 114.6 (d, J_{CF} = 2.6 Hz), 108.6 (d, J_{CF} = 25.5 Hz), 104.2 (d, J_{CF} = 26.8 Hz). **FTIR (KBr 1%, cm−1): $\tilde{\nu}$ =** 3283,

3191, 3113, 1584, 1538, 1511, 1458, 1414, 1387, 1141, 801, 763. **^{19}F NMR (282 MHz, DMSO-d6, δ ppm):** -110.31 (dd, J_{HF} = 8.7 and 12.3 Hz). **HRMS m/z (ESI):** calcd for $C_{18}H_{12}N_3F^{35}Cl$ $(M + H)^+$ 324.0698, found 324.0696 (1 ppm). **Mp:** 233-235°C.

Compound 4q: *N*-(3-bromo-5-chlorophenyl)benzo[*h*]quinazolin-2-amine

65% Yield (Light brown solid) **4q**: **^{1}H NMR (400 MHz, DMSO-d6, δ ppm):** 10.44 (br s, 1H, NH), 9.39 (s, 1H), 8.96 (d, J = 7.6 Hz, 1H), 8.37 (t, J = 1.6 Hz, 1H), 8.23 (t, J = 1.6 Hz, 1H), 8.07 (d, J = 7.6 Hz, 1H), 7.87-7.79 (m, 4H), 7.32 (t, J = 1.6 Hz, 1H). **^{13}C NMR (75 MHz, DMSO-d6, δ ppm):** 161.5, 157.4, 150.8, 143.7, 136.2, 134.6, 130.7, 129.0, 128.8, 127.6, 125.3, 124.3, 124.1, 123.4, 122.6, 119.9, 118.3, 117.4. **FTIR (KBr 1%, cm−1):** $\tilde{\nu}$ = 3286, 3107, 3044, 1578, 1530, 1509, 1450, 1406, 1387, 755. **HRMS m/z (ESI):** calcd for $C_{18}H_{12}N_3F^{35}Cl$ $(M + H)^+$ 383.98976, found 383.9895 (1 ppm). **Mp:** 256-258°C.

Compound 4r: *N*-(4-bromo-3-methylphenyl)benzo[*h*]quinazolin-2-amine

66% Yield (Off white solid) **4r**: **^{1}H NMR (400 MHz, DMSO-d6, δ ppm):** 10.11 (br s, 1H, NH), 9.31 (s, 1H), 8.98 (dd, J = 7.2, 1.2 Hz, 1H), 8.06-8.02 (m, 2H), 7.90-7.87 (m, 1H), 7.84-7.74 (m, 4H), 7.59 (d, J = 8.4 Hz, 1H), 2.42 (s, 3H). **^{13}C NMR (75 MHz, DMSO-d6, δ ppm):** 161.2, 157.9, 151.1, 140.6, 137.5, 136.1, 132.5, 130.5, 129.1, 128.6, 124.6, 124.5, 124.3, 121.6, 118.8, 117.8, 115.9, 23.4. **FTIR (KBr 1%, cm−1):** $\tilde{\nu}$ = 3428, 3273, 2254, 2126, 1609, 1584, 1542, 1450, 1510, 1023, 1000, 822, 790, 754. **HRMS m/z (ESI):** calcd for $C_{19}H_{15}N_3{}^{79}Br$ $(M + H)^+$ 364.04438, found 364.0448 (1 ppm). **Mp:** 201-202°C.

Compound 4s: *N*-(3,4,5-trifluorophenyl)benzo[*h*]quinazolin-2-amine

65% Yield (Light brown solid) **4s**: **^{1}H NMR (400 MHz, DMSO-d6, δ ppm):** 10.42 (br s, 1H, NH), 9.37 (s, 1H), 8.95-8.93 (m, 1H), 8.07-8.05 (m, 1H), 8.01-7.97 (m, 2H), 7.87-7.80 (m, 4H). **^{13}C NMR (75 MHz, DMSO-d6, δ ppm):** 161.3, 157.3, 150.8, 150.6 (ddd, JCF = 5.8, 10,7 and 242.6 Hz), 136.5 (dtd, J_{CF} = 3.3, J_{FF} = 11.8 and 149.5 Hz), 132.2 (m), 130.6, 129.0, 128.7, 127.5, 125.1, 124.2, 124.1, 118.2, 103.0, 102.6. **^{19}F NMR (282 MHz, DMSO-d6, δ ppm):** -130.36 and -130.46 (dd, J_{HF} = 11.0 Hz, J_{FF} = 22.1 Hz), -166.03 (tt, J_{HF} = 6.7 Hz, J_{FF} = 22.1 Hz). **HRMS m/z (ESI):** calcd for $C_{18}H_{11}N_3F_3$ $(M + H)^+$ 326.0899, found 326.0900 (0 ppm). **FTIR (KBr 1%, cm−1):** $\tilde{\nu}$ **=** 3288, 3111, 3067, 1614, 1586, 1548, 1520, 1440, 1391, 1288, 1223, 1036, 847, 792, 761. **Mp:** 252-254°C.

Spectra: ^1H, ^{13}C, ^{13}C Jmod NMR and FT-IR data of compound 3

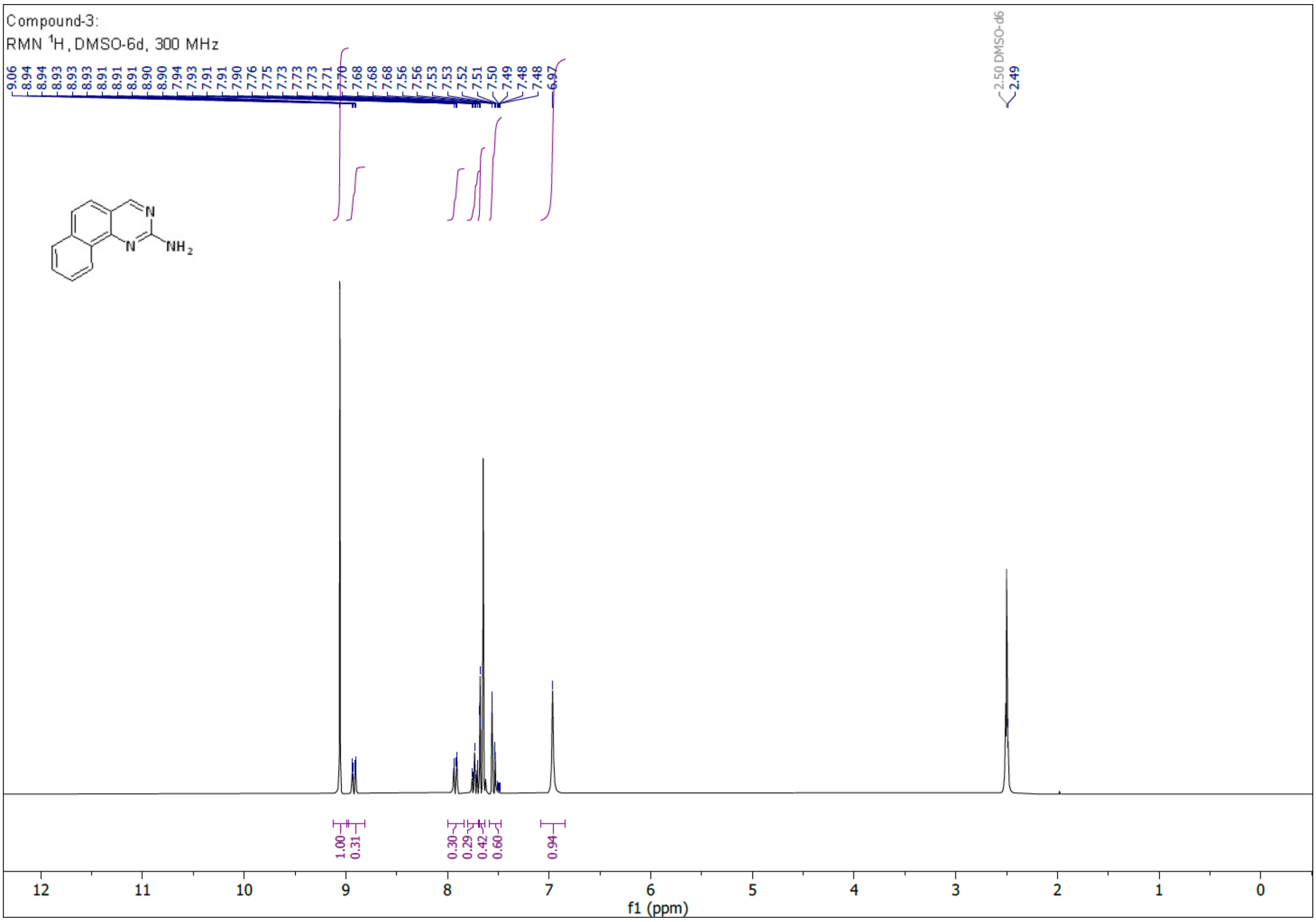

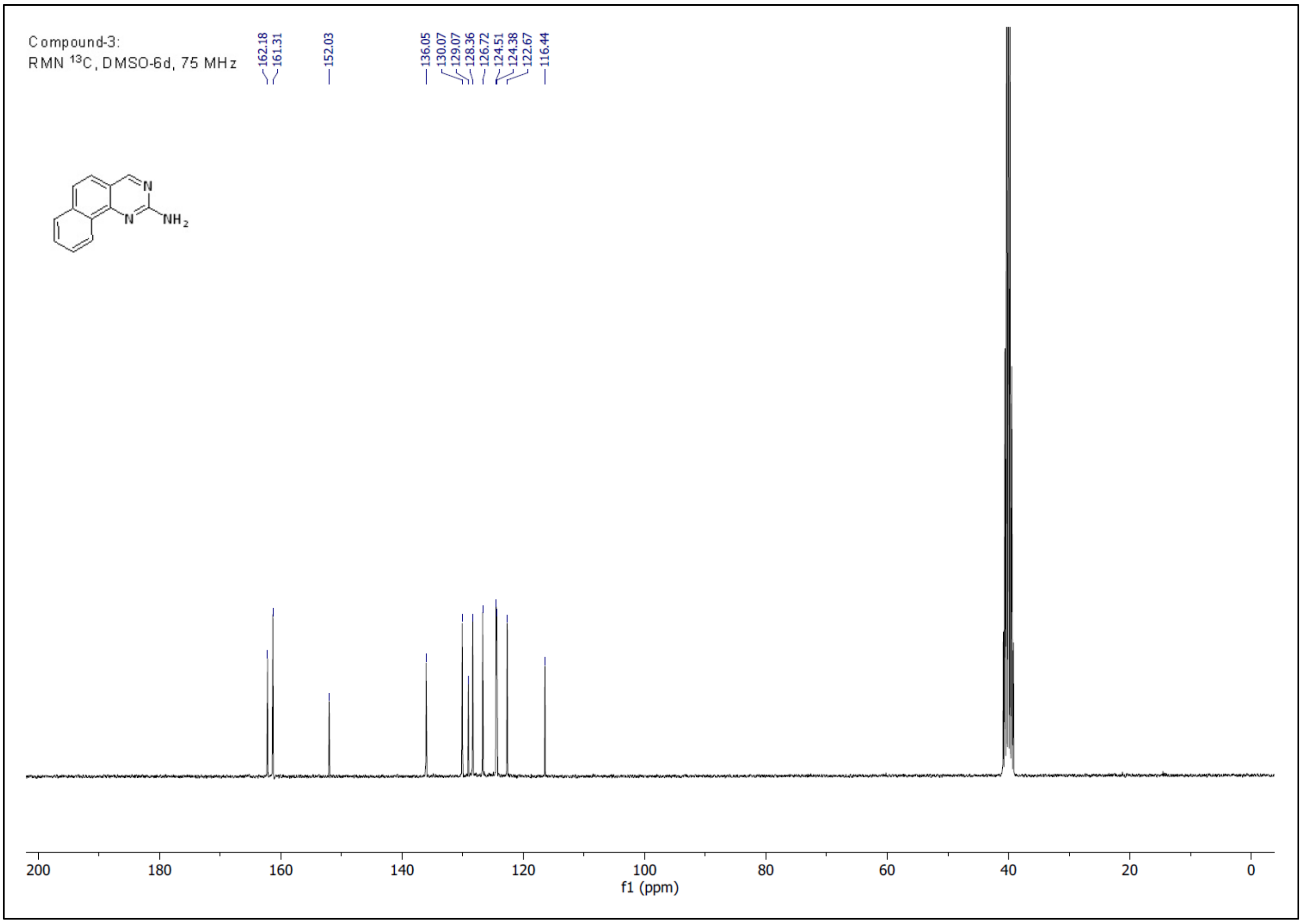

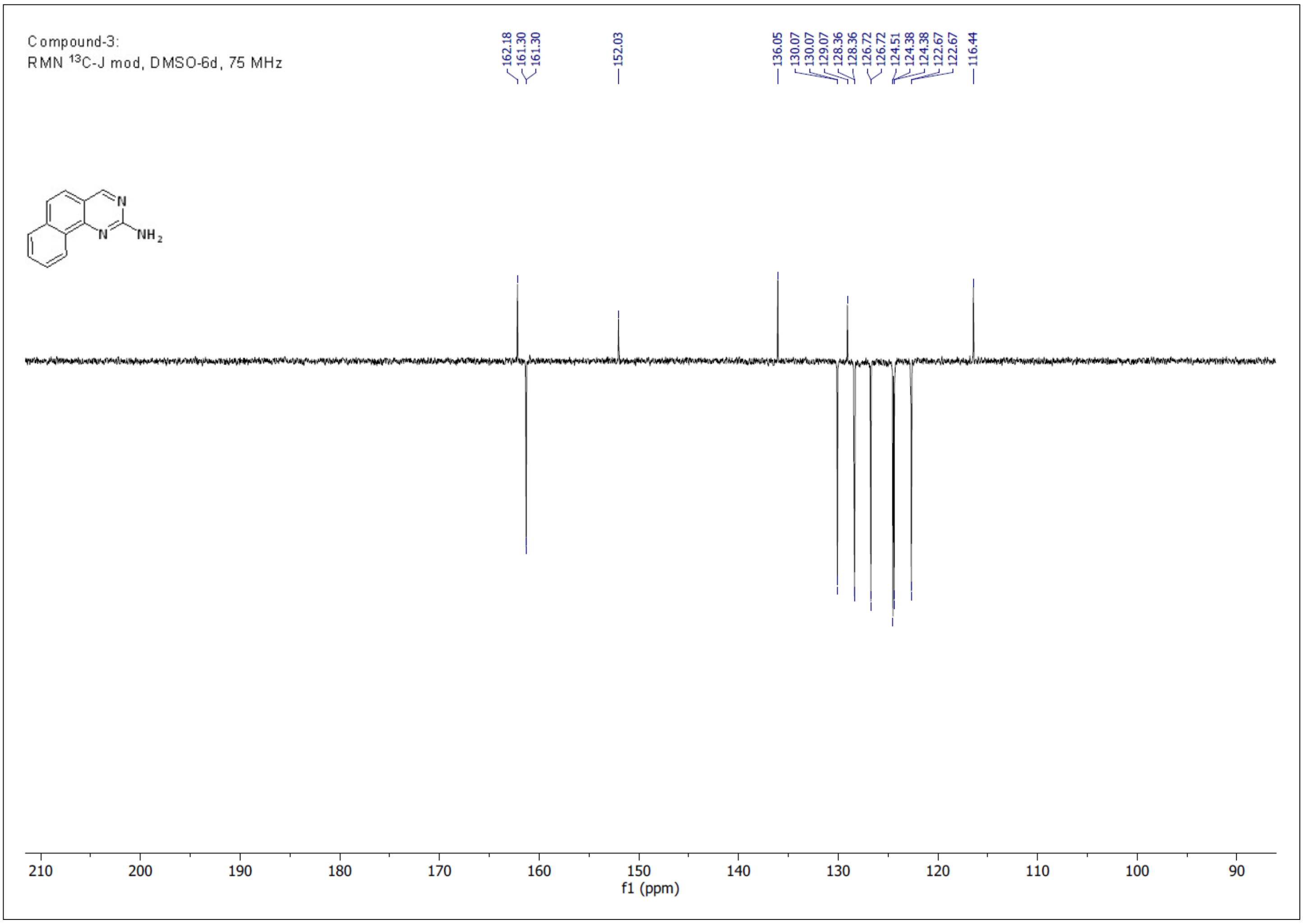
Compound-3:
RMN 13C-J mod, DMSO-6d, 75 MHz
162.18
161.30
161.30
152.03
136.05
130.07
130.07
129.07
128.36
128.36
126.72
126.72
124.51
124.38
124.38
122.67
122.67
116.44
N
N
NH2
210
200
190
180
170
160
150
140
130
120
110
100
90
f1 (ppm)

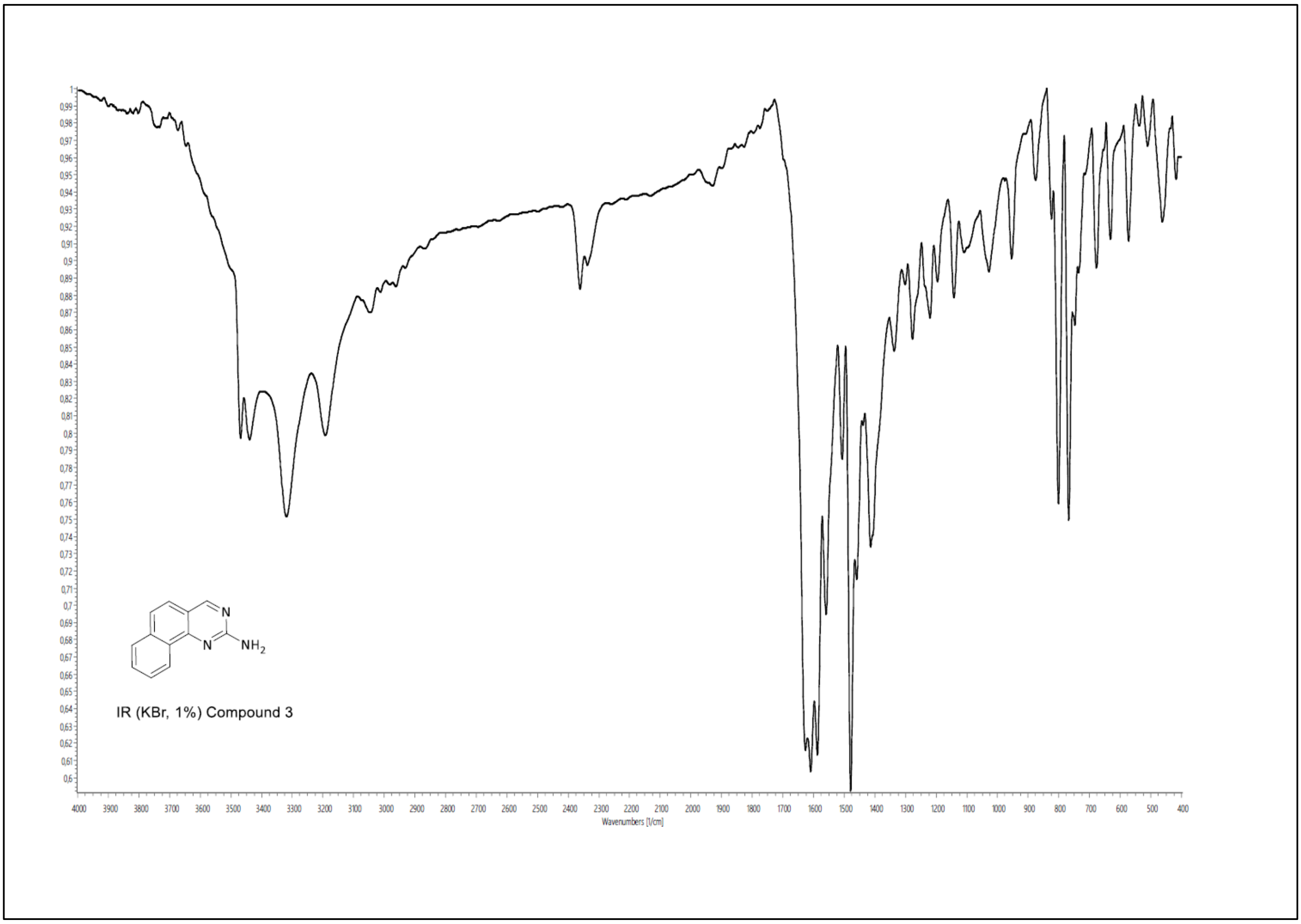
N
N
NH2
IR (KBr, 1%) Compound 3
Wavenumbers [1/cm]

Spectra: ^{1}H, ^{13}C, ^{13}C Jmod NMR and FT-IR data of compound 4a (DV-02)

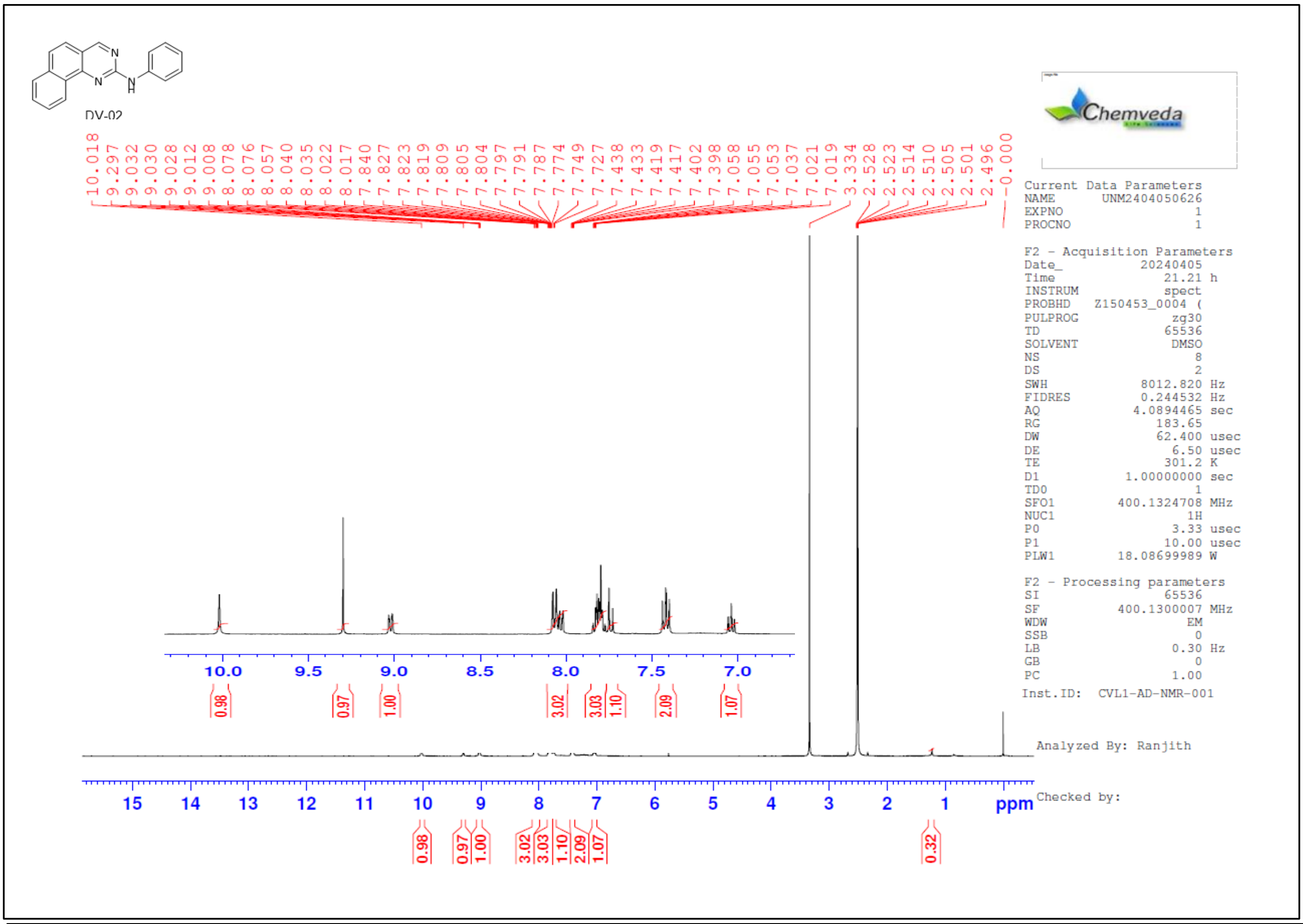

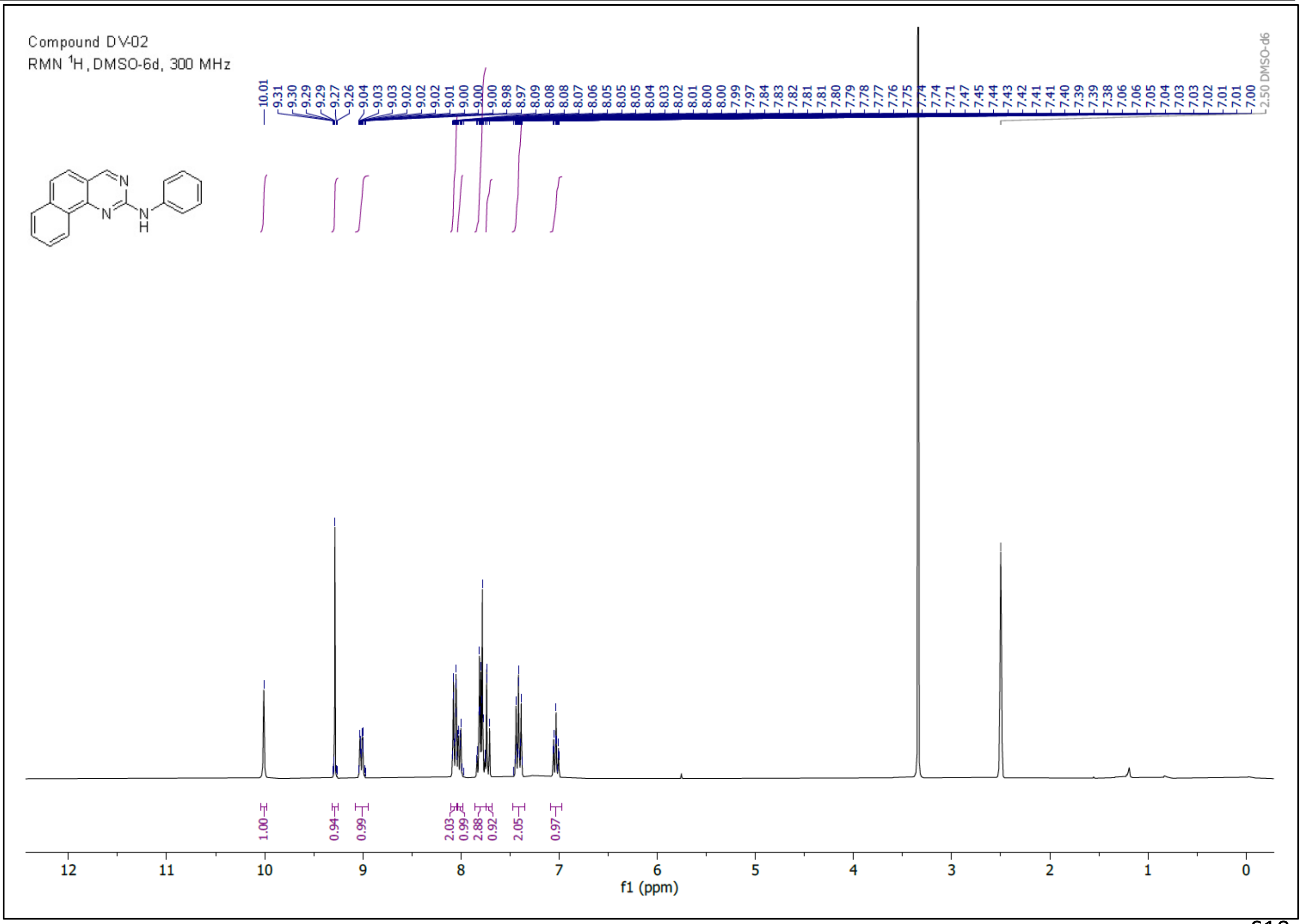

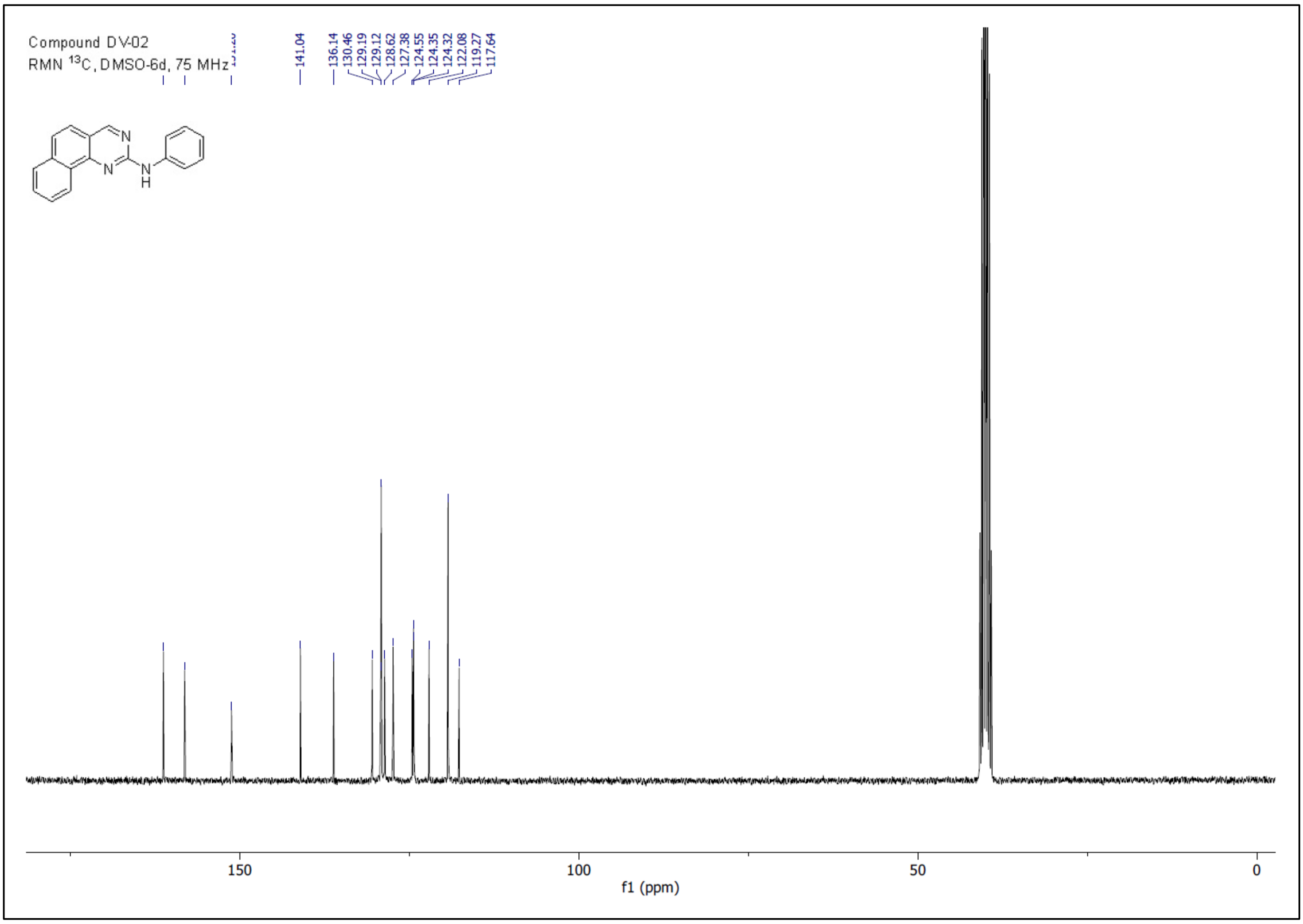
Compound DV-02
RMN 13C, DMSO-6d, 75 MHz
141.04
136.14
130.46
129.19
129.12
128.62
127.38
124.55
124.35
124.32
122.08
119.27
117.64
N
N
N
H
150
100
50
0
f1 (ppm)

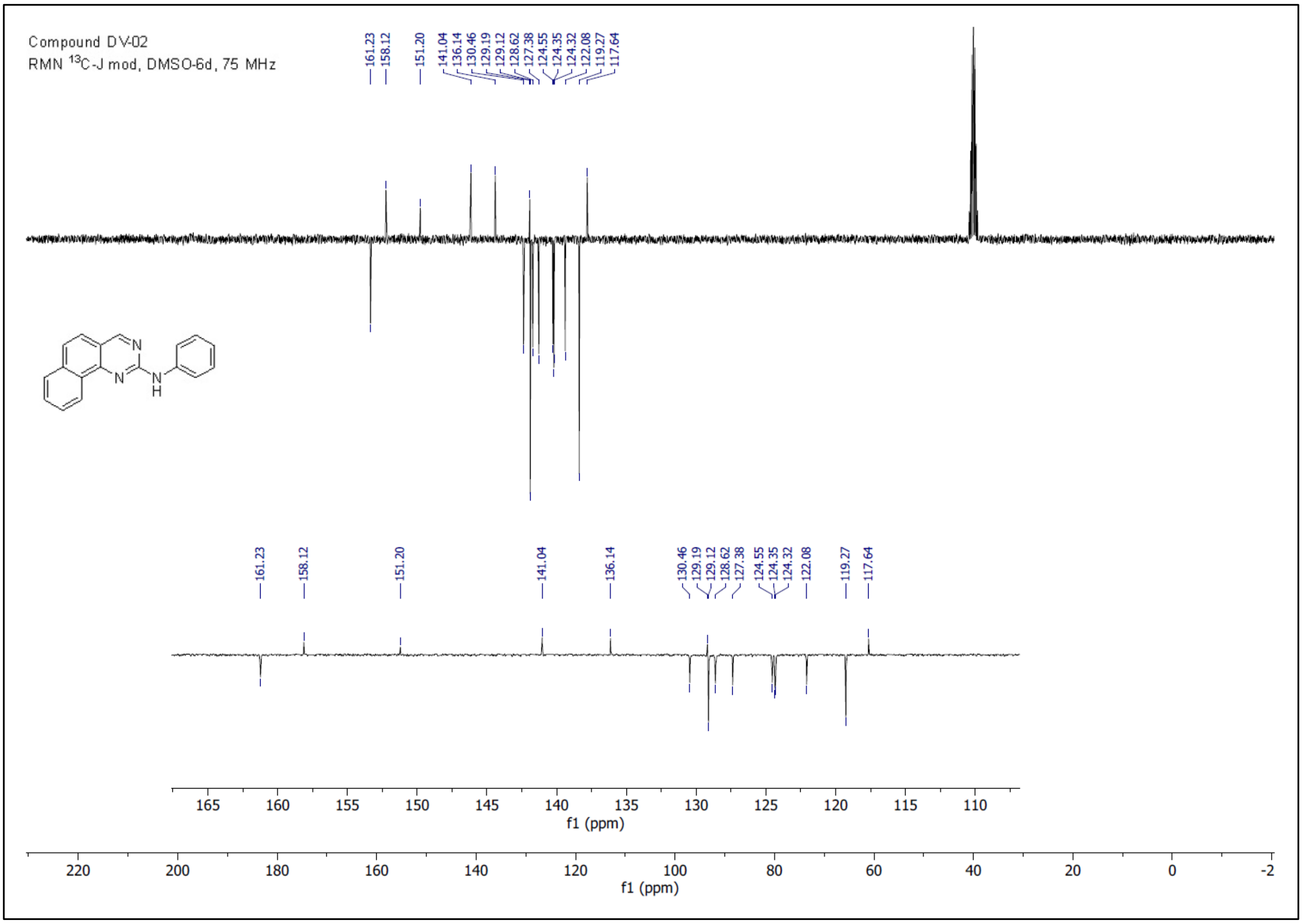
Compound DV-02
RMN 13C-J mod, DMSO-6d, 75 MHz
161.23
158.12
151.20
141.04
136.14
130.46
129.19
129.12
128.62
127.38
124.55
124.35
124.32
122.08
119.27
117.64
N
N
N
H
165
160
155
150
145
140
135
130
125
120
115
110
f1 (ppm)
220
200
180
160
140
120
100
80
60
40
20
0
-2
f1 (ppm)

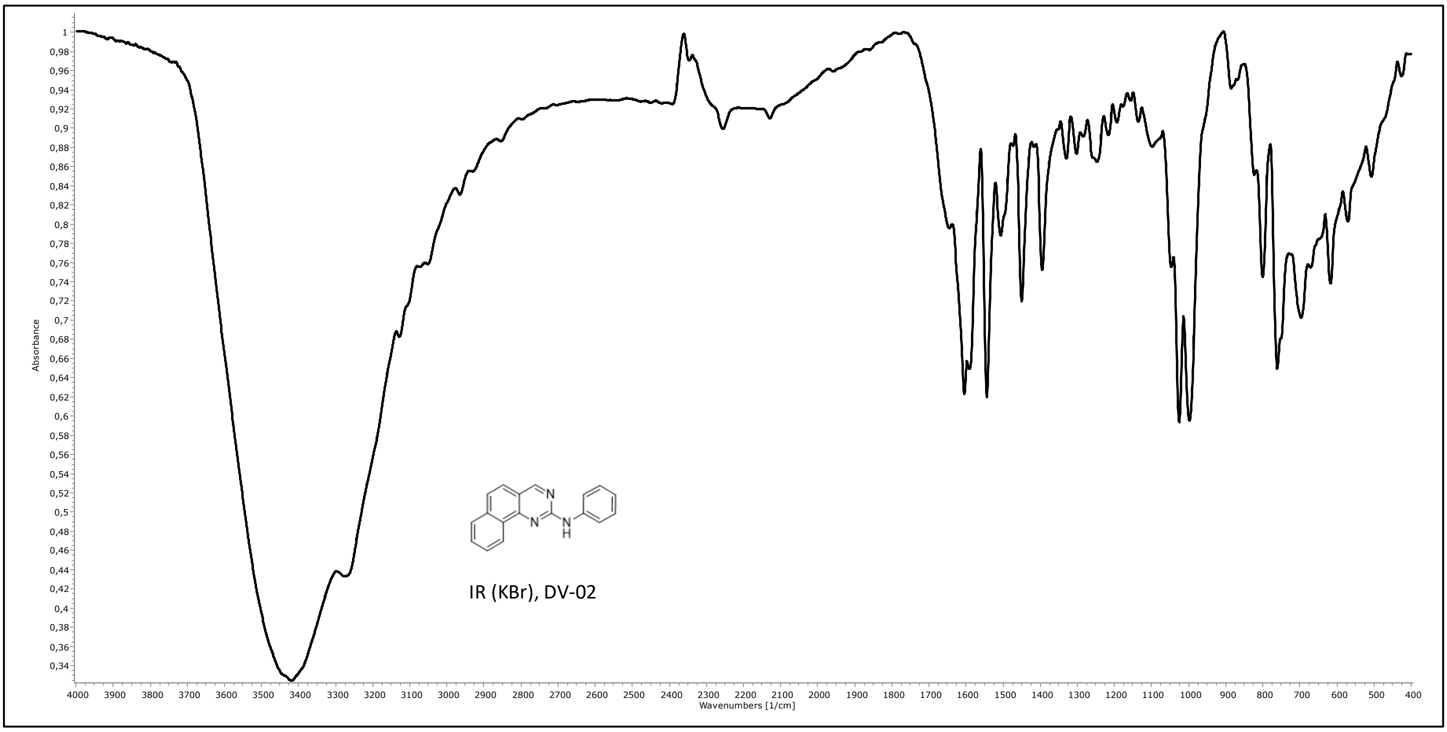
IR (KBr), DV-02
N
N
N
H
Absorbance
Wavenumbers [1/cm]

Spectra: ^{1}H, ^{13}C, ^{13}C Jmod NMR and FT-IR data of compound 4b (DV-03)

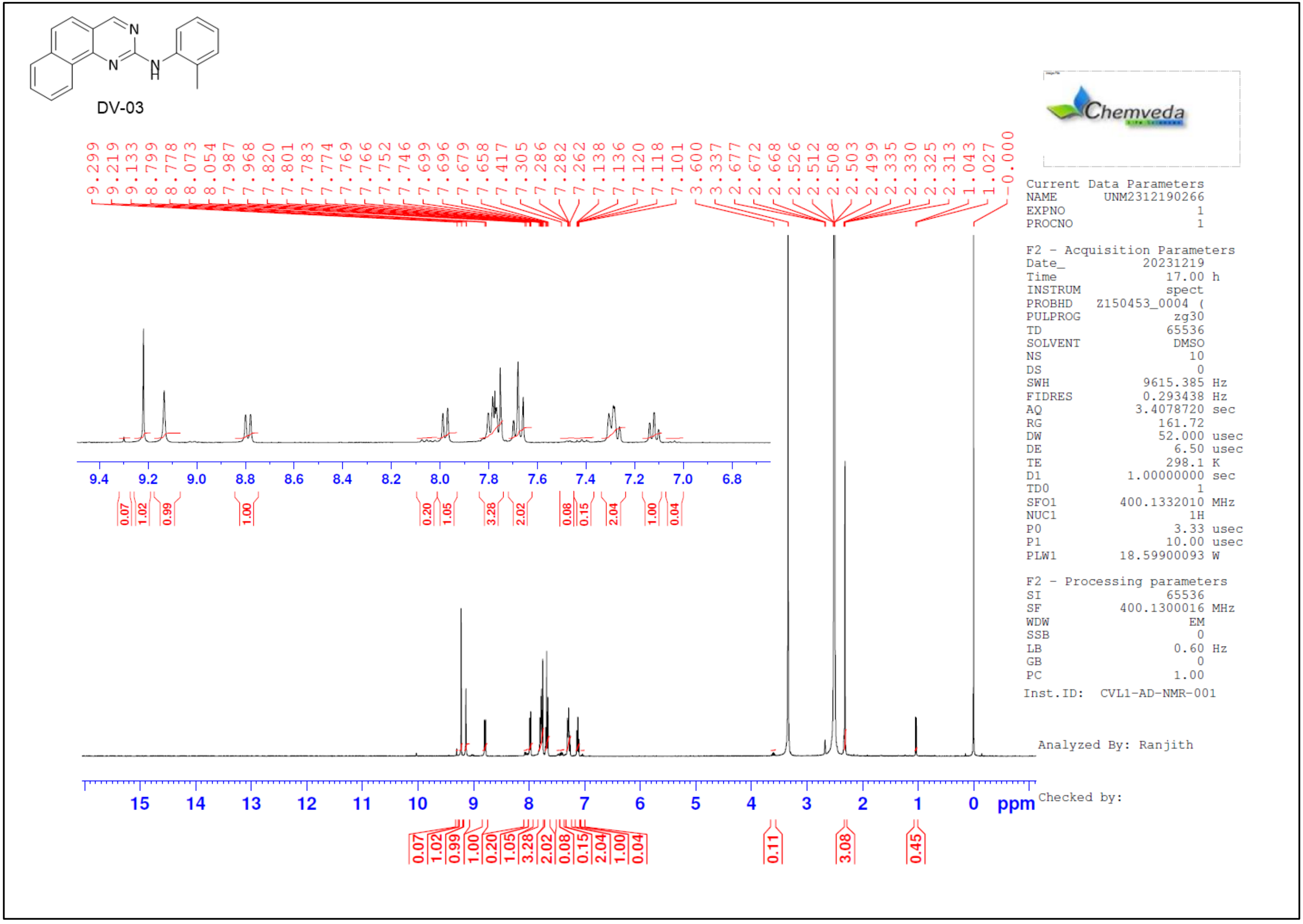

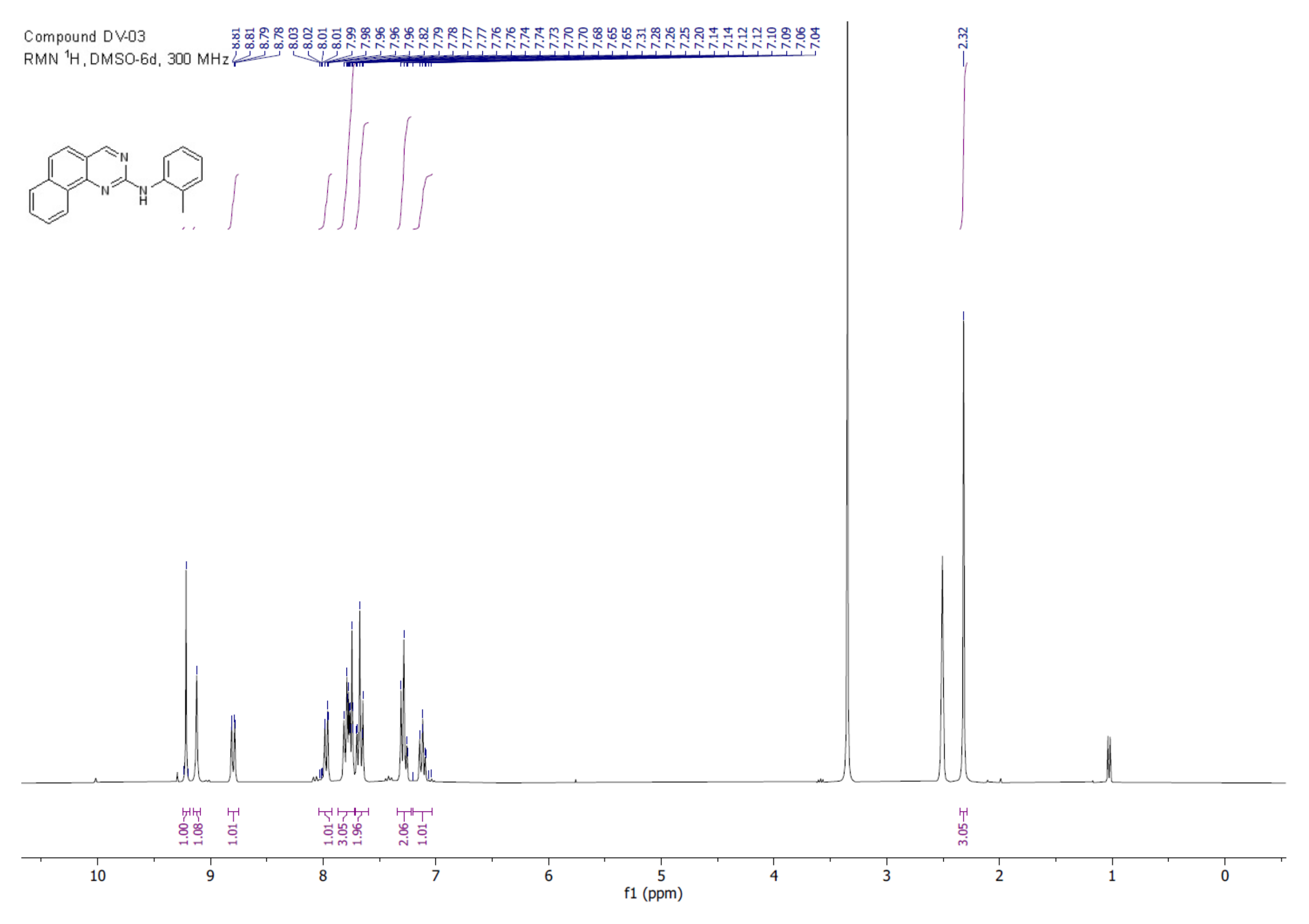

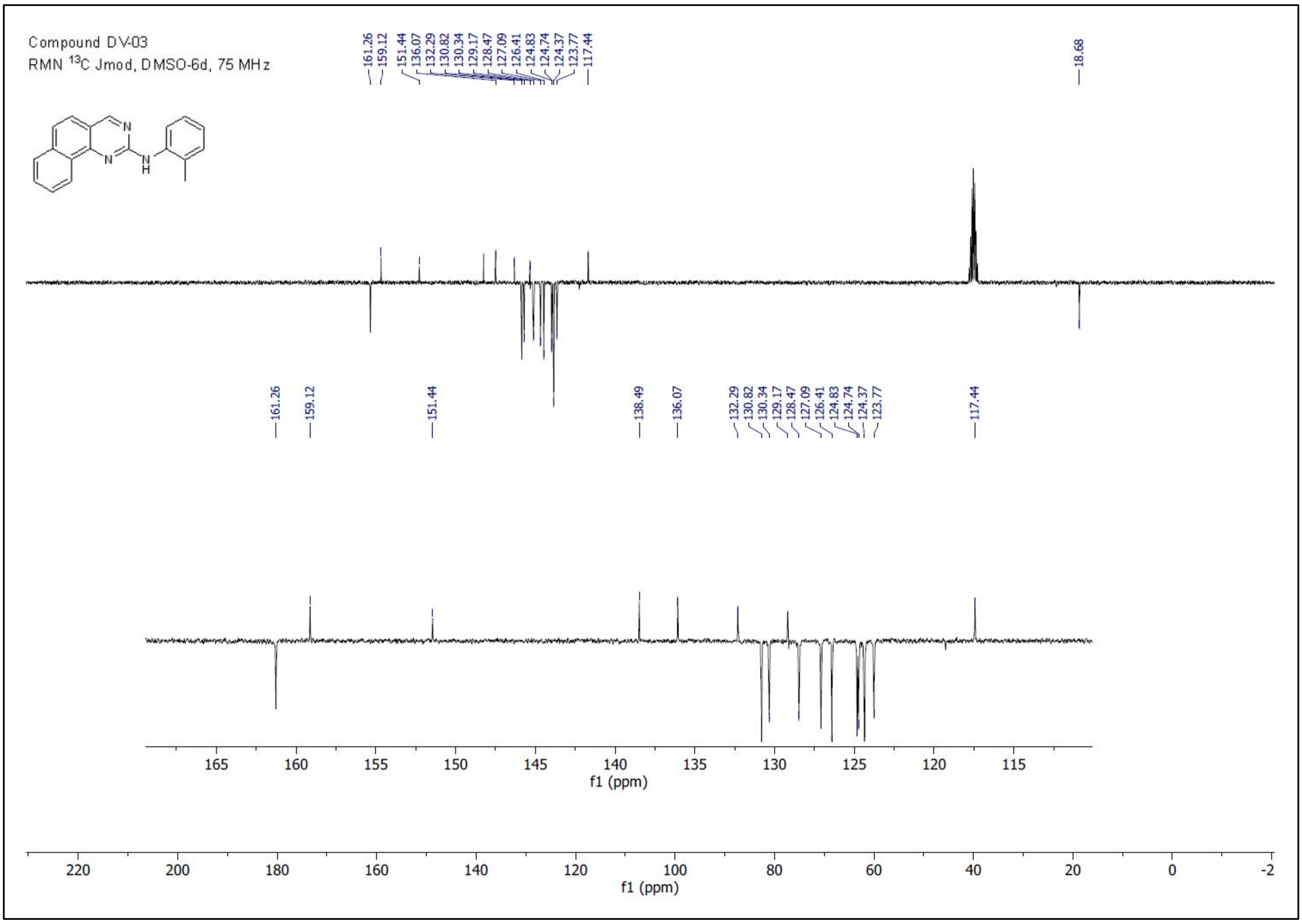
Compound DV-03
RMN 13C Jmod, DMSO-6d, 75 MHz
161.26
159.12
151.44
138.49
136.07
132.29
130.82
130.34
129.17
128.47
127.09
126.41
124.83
124.74
124.37
123.77
117.44
18.68
f1 (ppm)

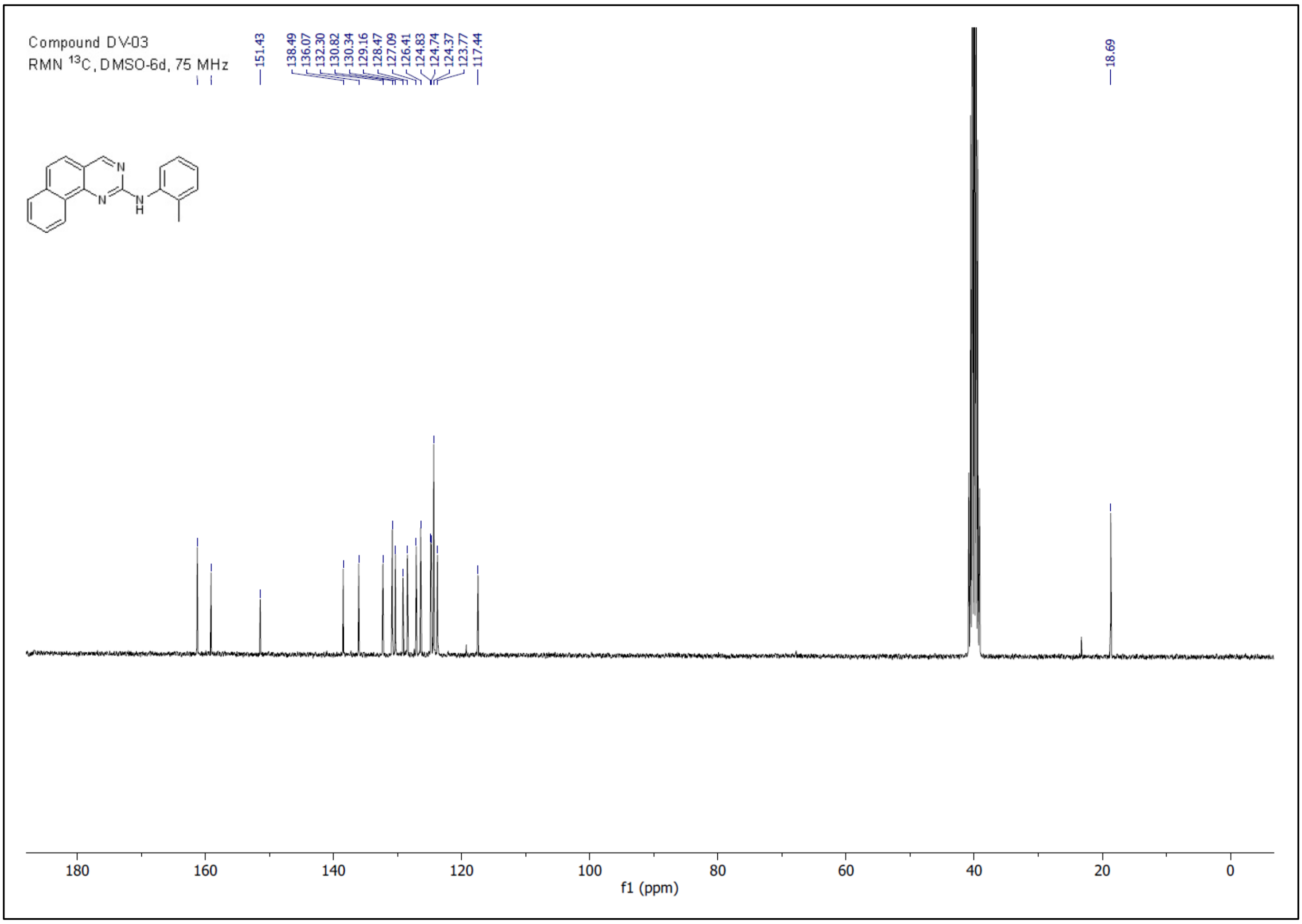
Compound DV-03
RMN 13C, DMSO-6d, 75 MHz
151.43
138.49
136.07
132.30
130.82
130.34
129.16
128.47
127.09
126.41
124.83
124.74
124.37
123.77
117.44
18.69
f1 (ppm)

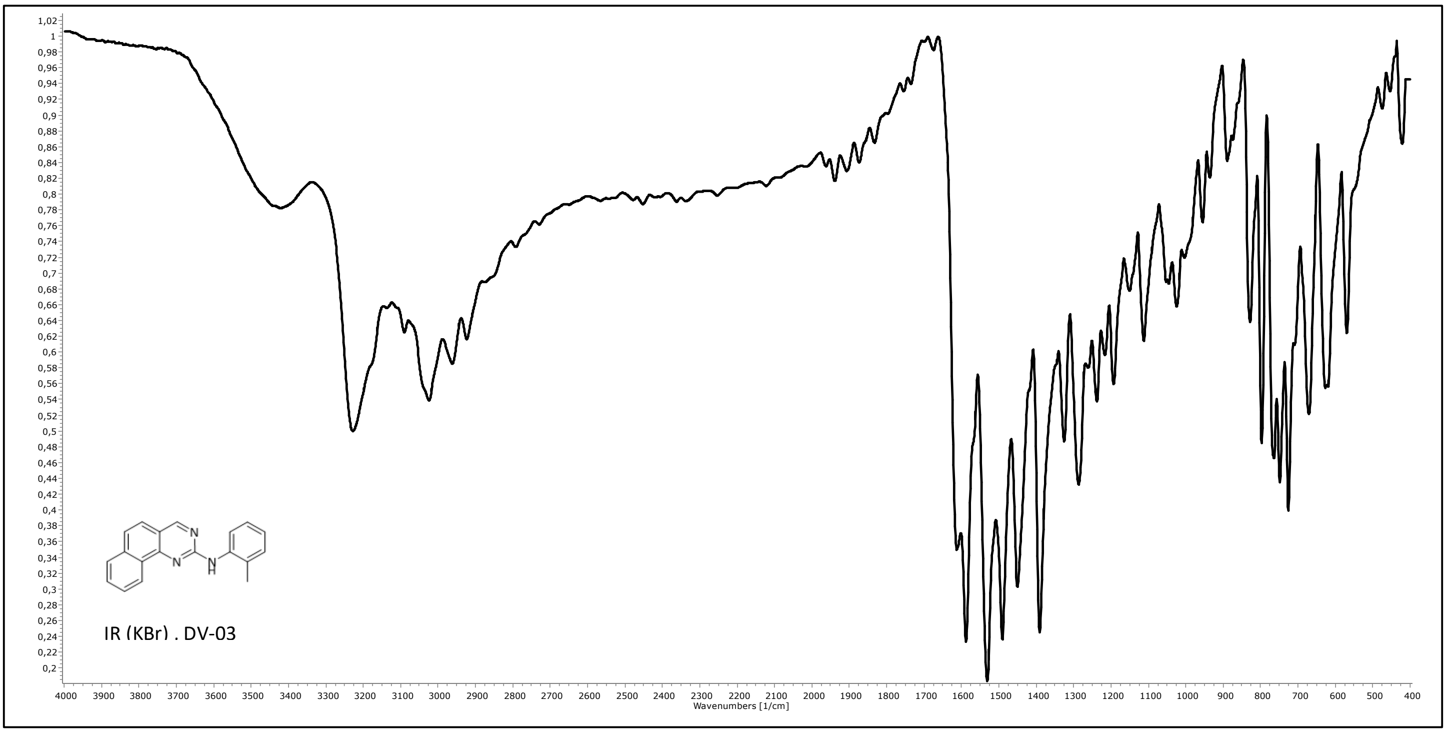
IR (KBr) . DV-03
Wavenumbers [1/cm]

Spectra: ^{1}H, ^{13}C, ^{13}C Jmod NMR and FT-IR data of compound 4c (DV-04)

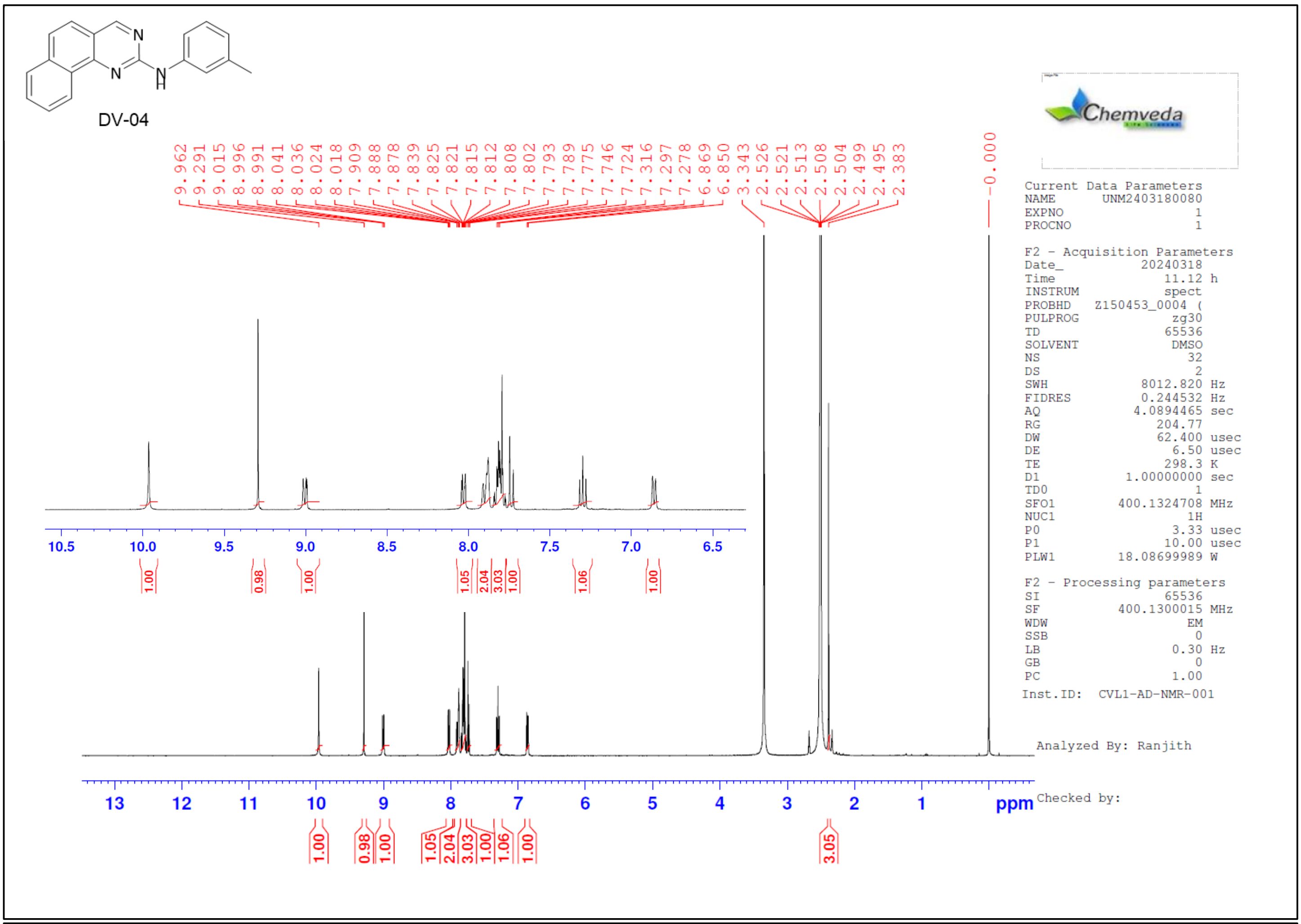

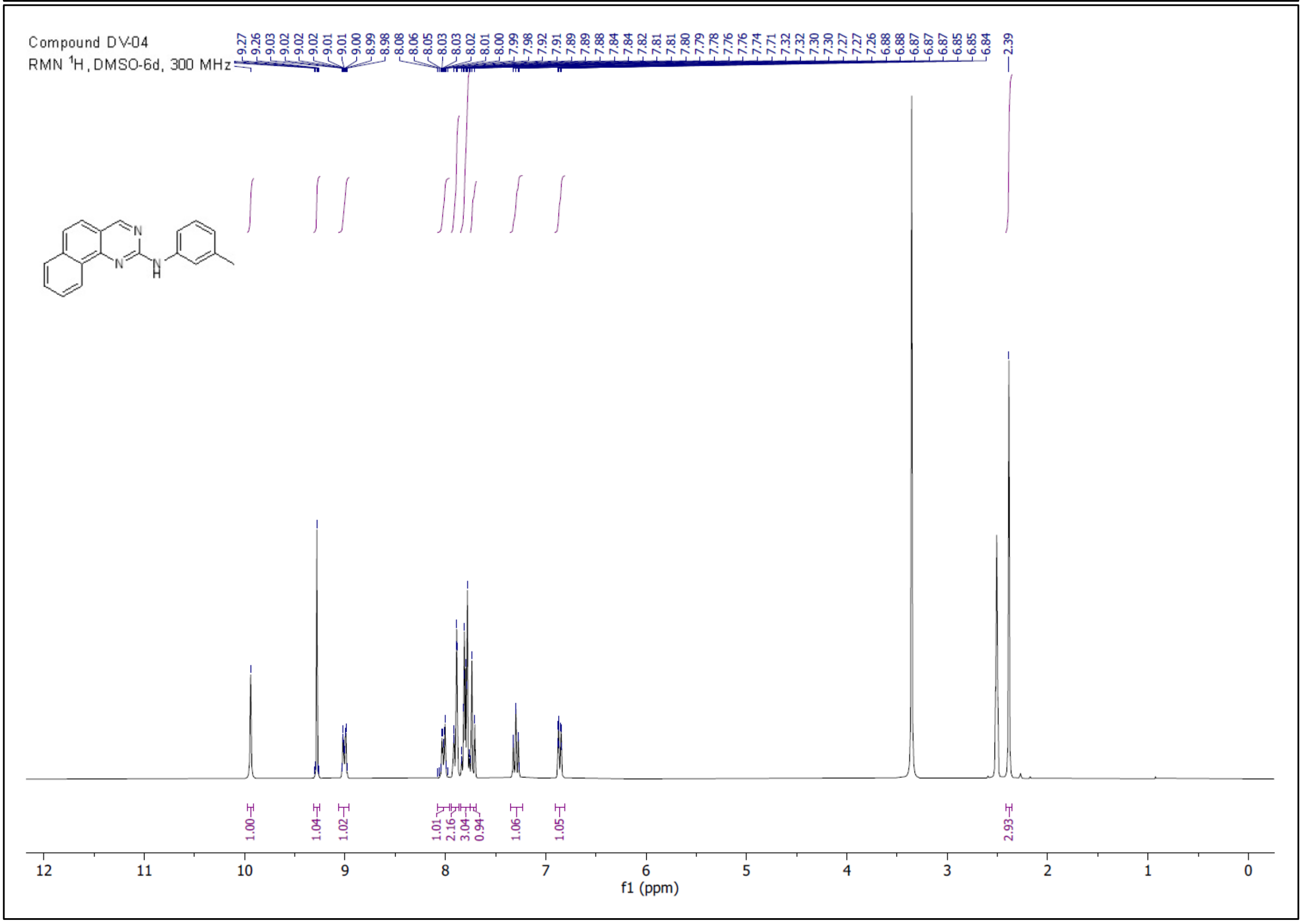

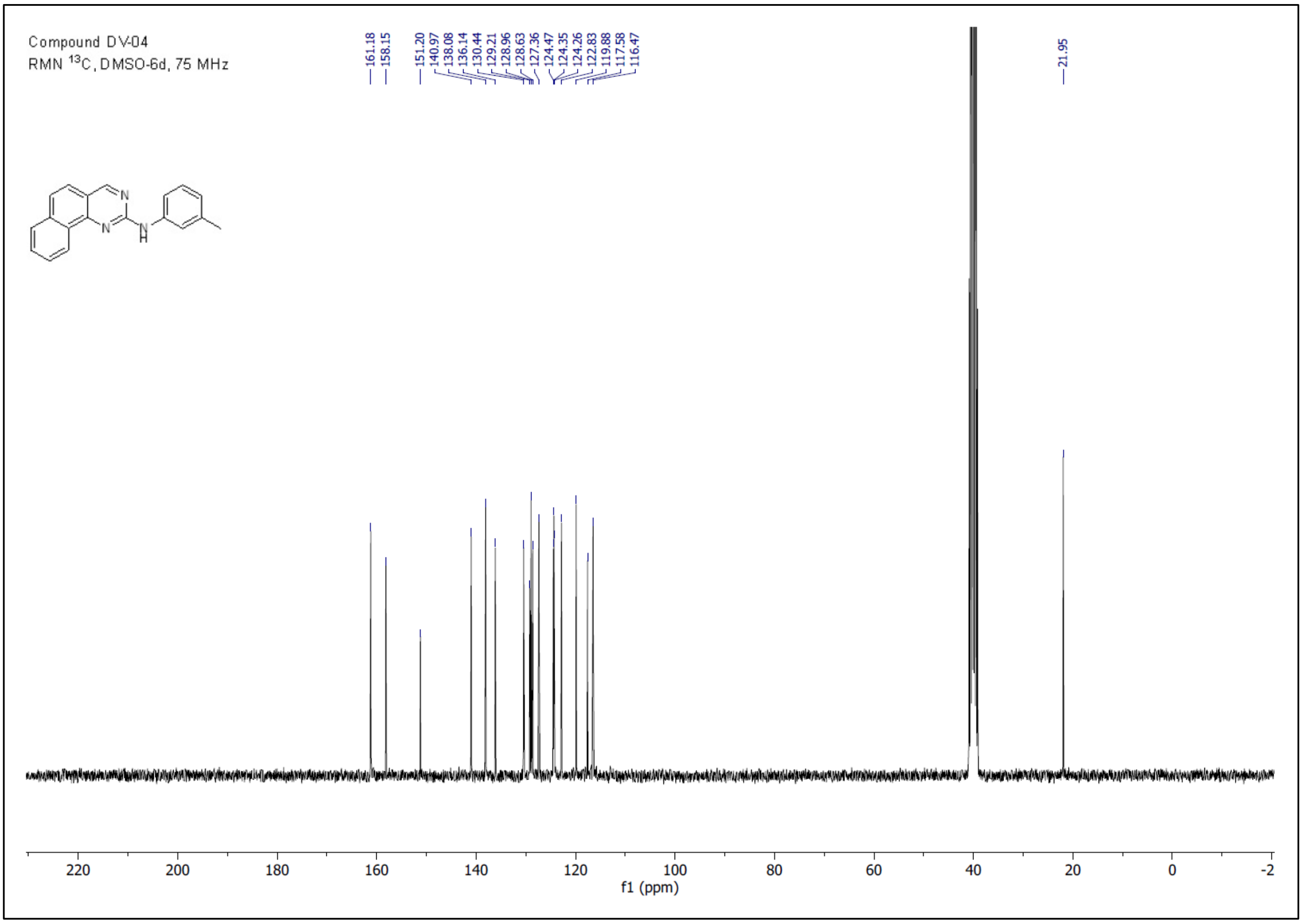
Compound DV-04
RMN ¹³C, DMSO-6d, 75 MHz
161.18
158.15
151.20
140.97
138.08
136.14
130.44
129.21
128.96
128.63
127.36
124.47
124.35
124.26
122.83
119.88
117.58
116.47
21.95
N
N
N
H
220
200
180
160
140
120
100
80
60
40
20
0
-2
f1 (ppm)

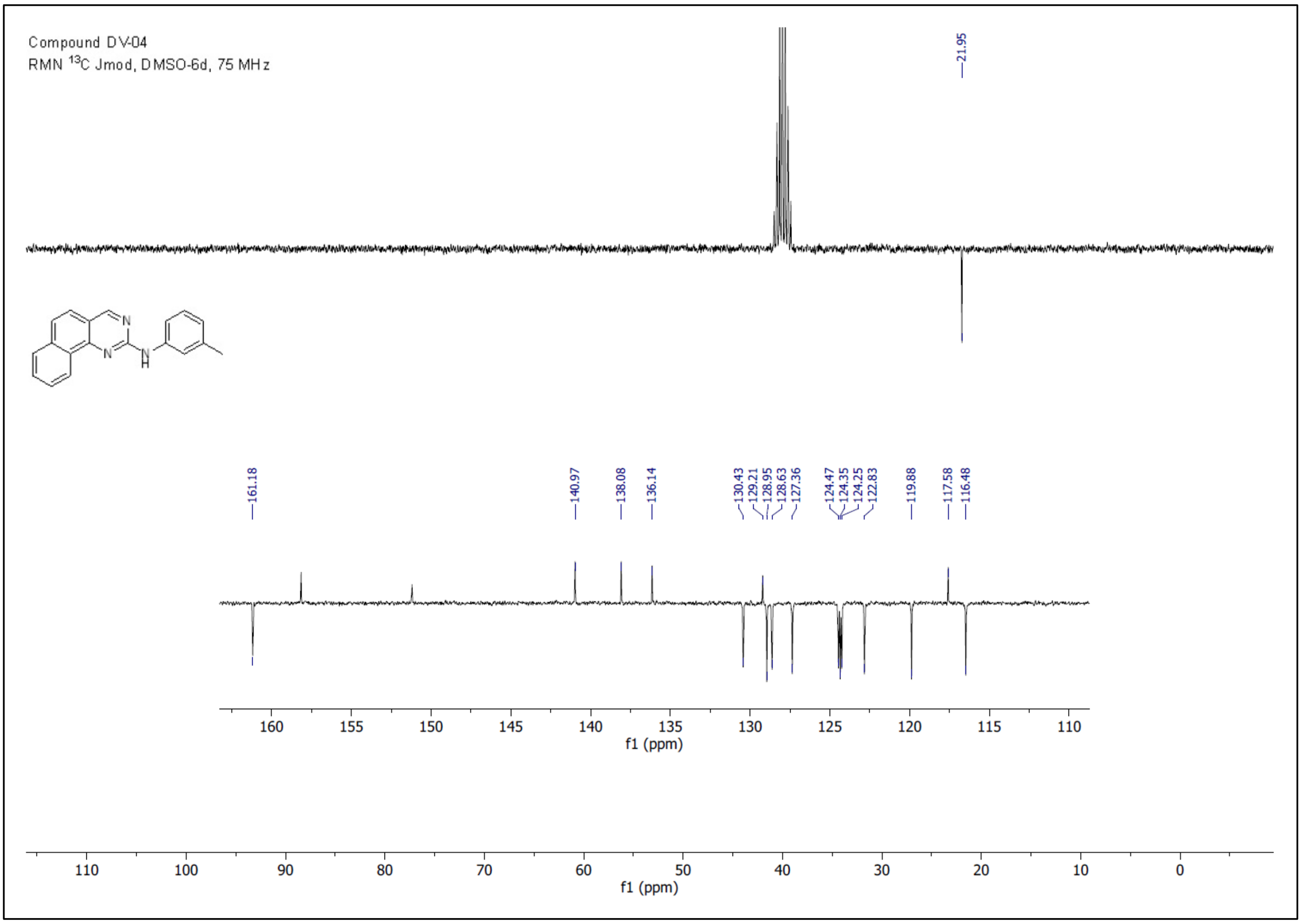
Compound DV-04
RMN ¹³C Jmod, DMSO-6d, 75 MHz
21.95
N
N
N
H
161.18
140.97
138.08
136.14
130.43
129.21
128.95
128.63
127.36
124.47
124.35
124.25
122.83
119.88
117.58
116.48
160
155
150
145
140
135
130
125
120
115
110
f1 (ppm)
110
100
90
80
70
60
50
40
30
20
10
0
f1 (ppm)

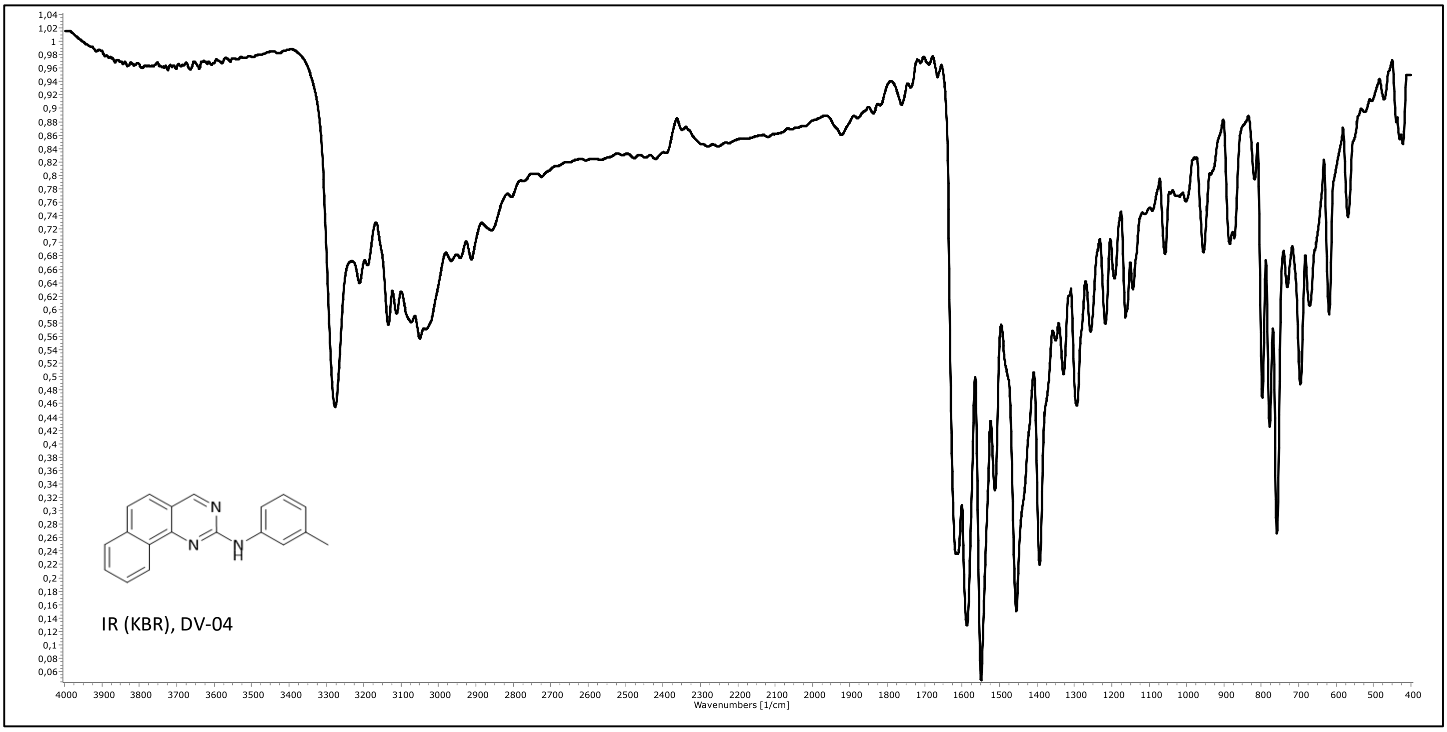
IR (KBR), DV-04
Wavenumbers [1/cm]

Spectra: ^{1}H, ^{13}C, ^{13}C Jmod NMR and FT-IR data of compound 4d (DV-05)

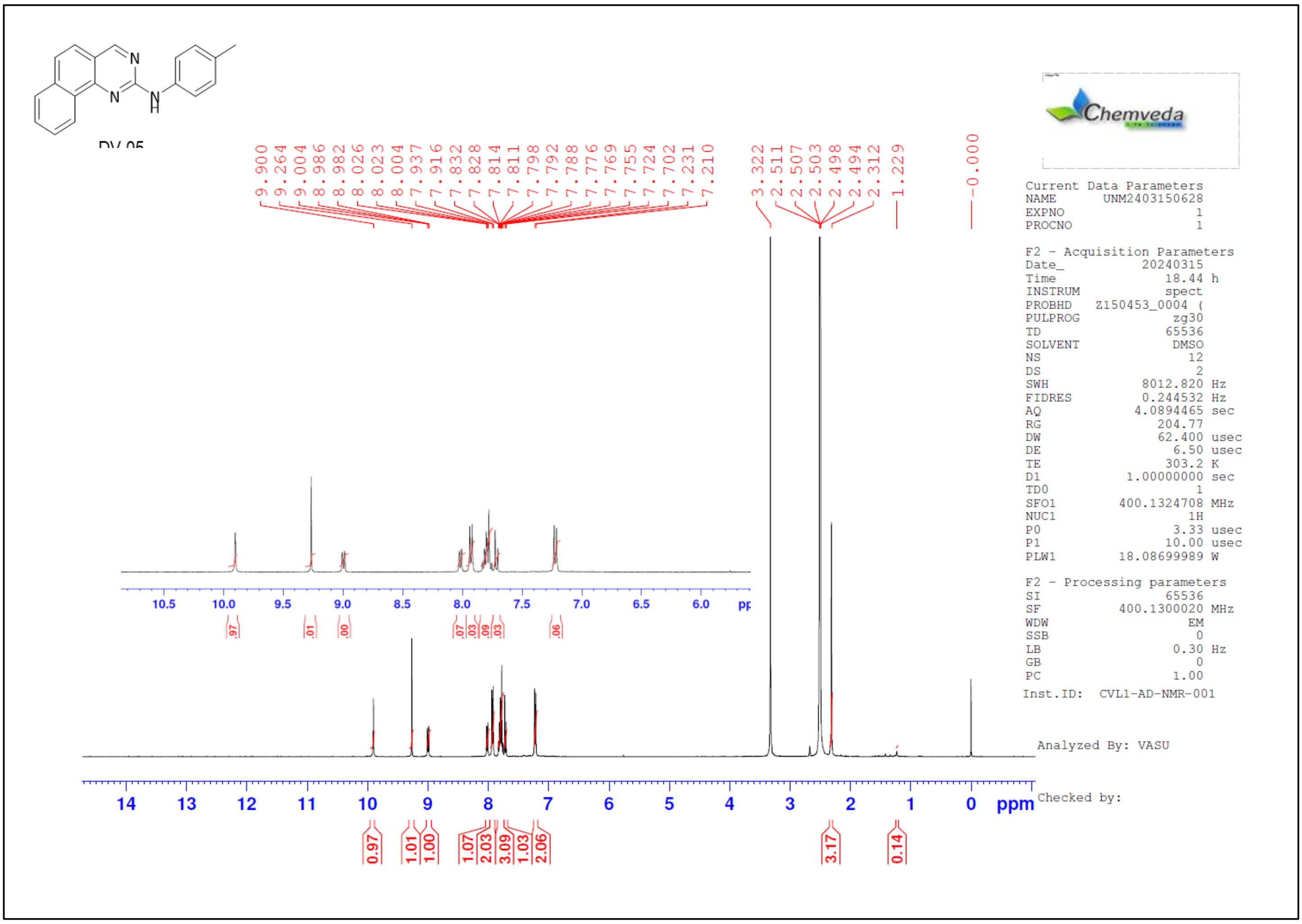

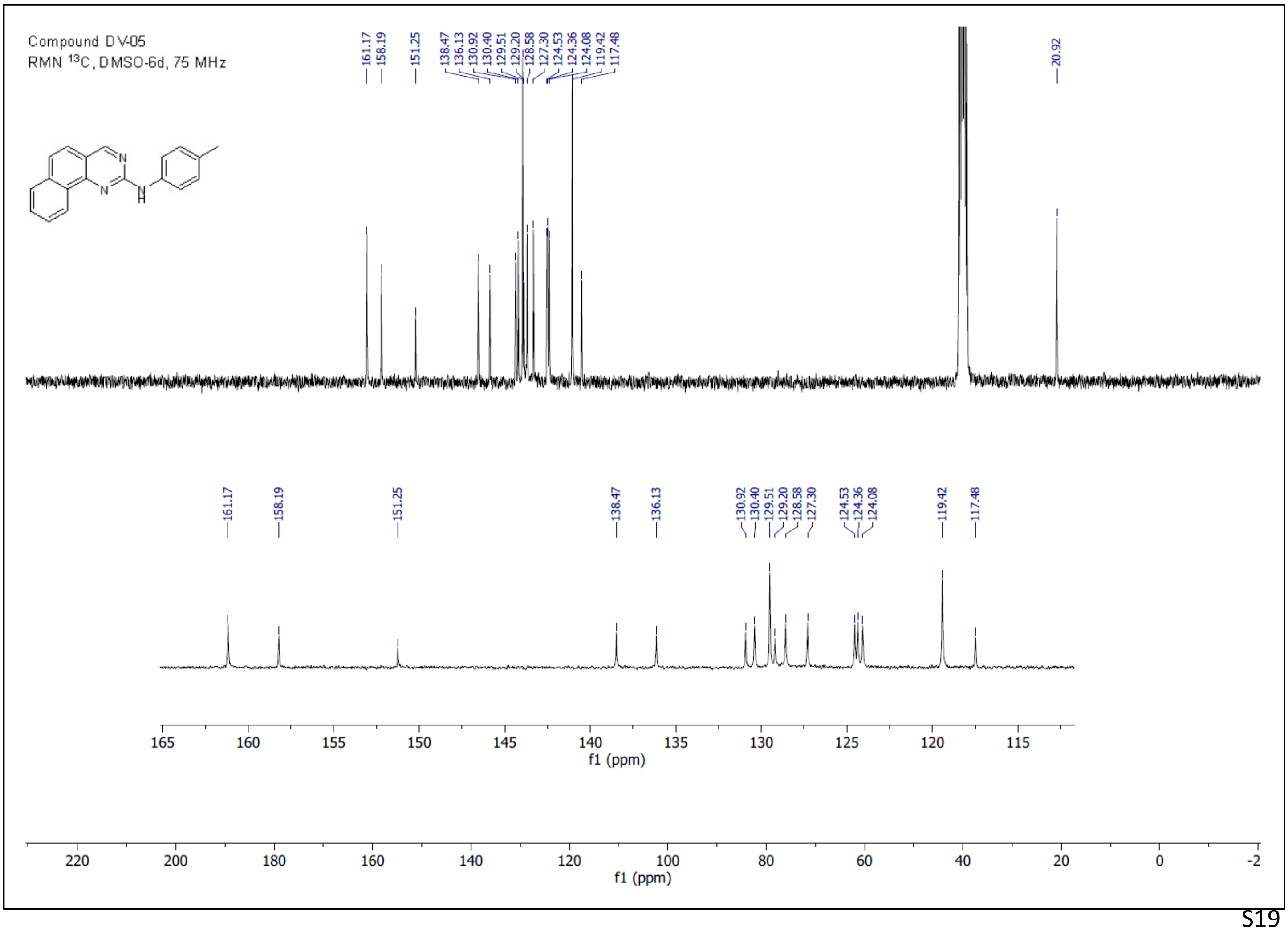

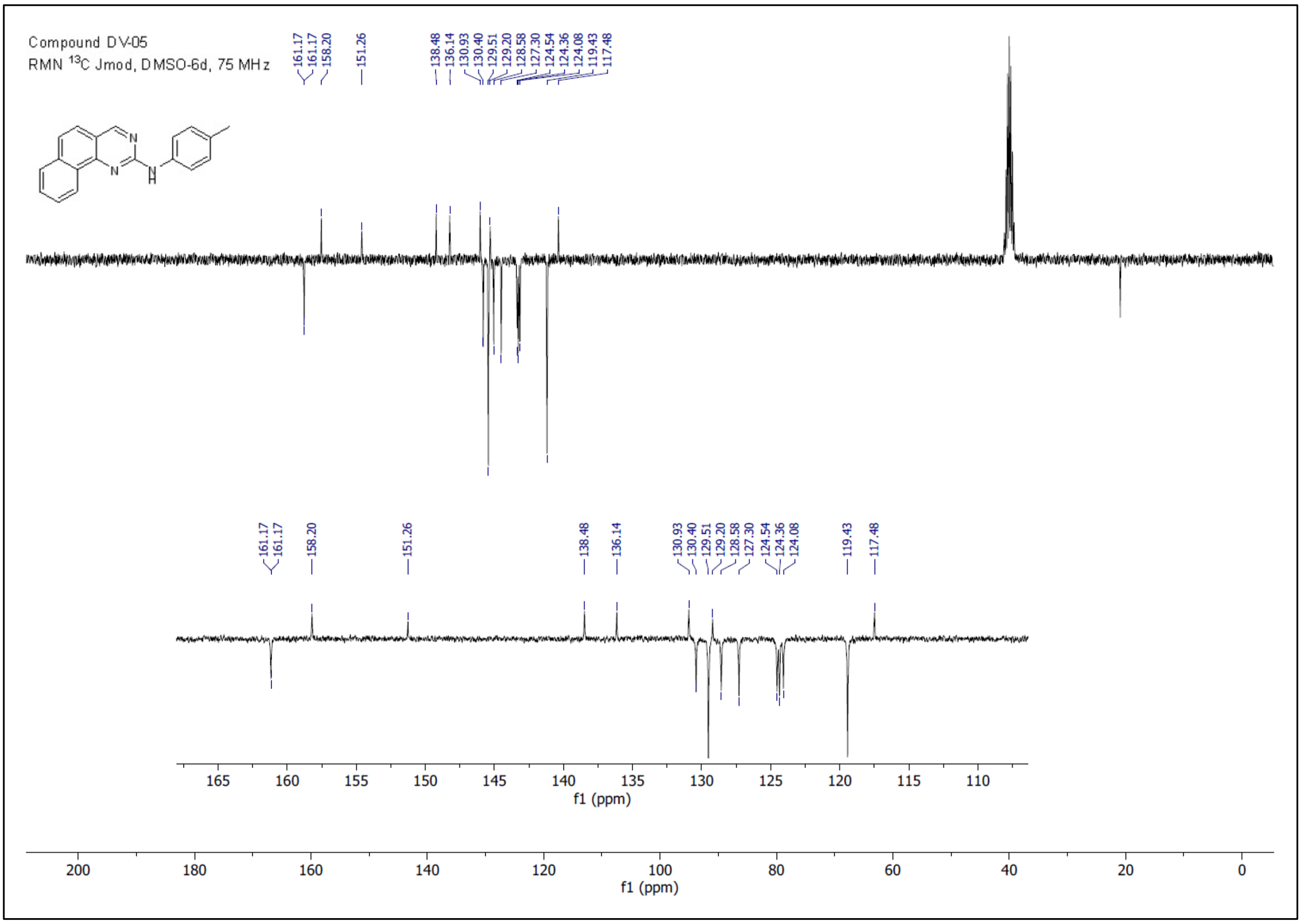
Compound DV-05
RMN ^{13}C Jmod, DMSO-6d, 75 MHz
161.17
161.17
158.20
151.26
138.48
136.14
130.93
130.40
129.51
129.20
128.58
127.30
124.54
124.36
124.08
119.43
117.48
f1 (ppm)

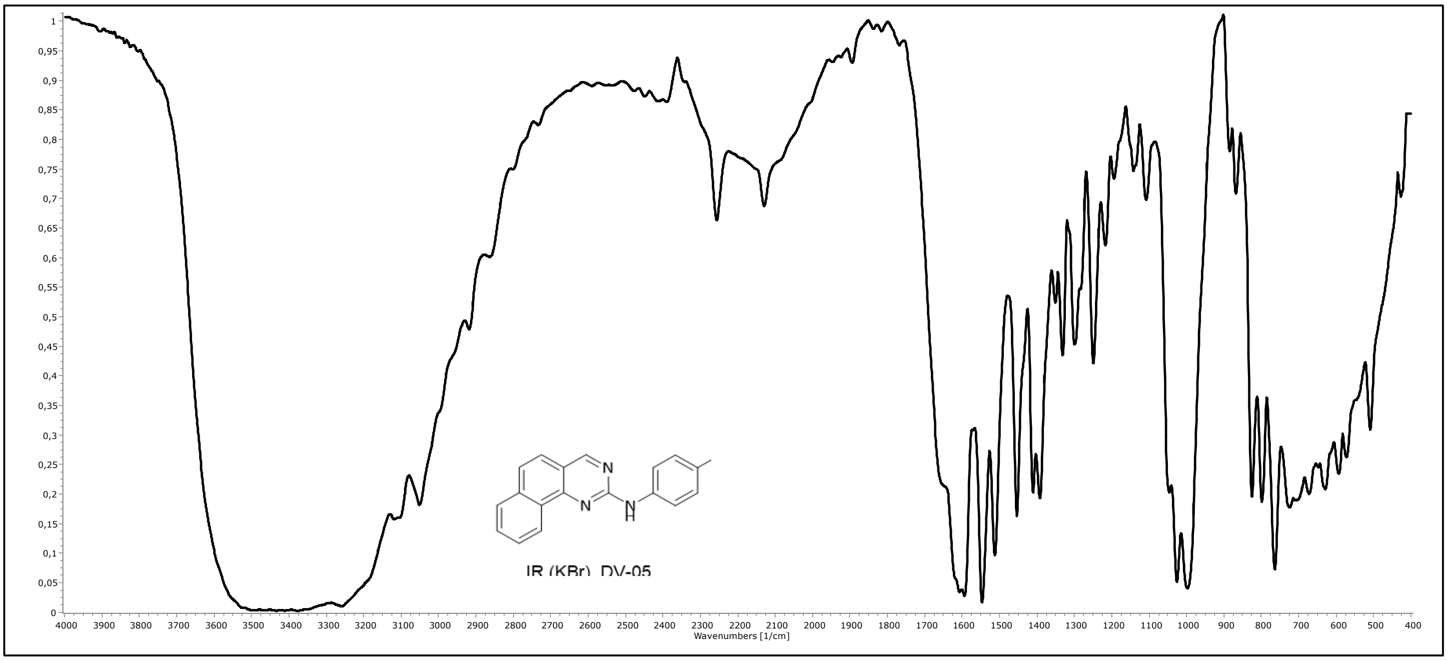
IR (KBr) DV-05
Wavenumbers [1/cm]

Spectra: ^{1}H, ^{13}C, ^{13}C Jmod NMR and FT-IR data of compound 4e (DV-06)

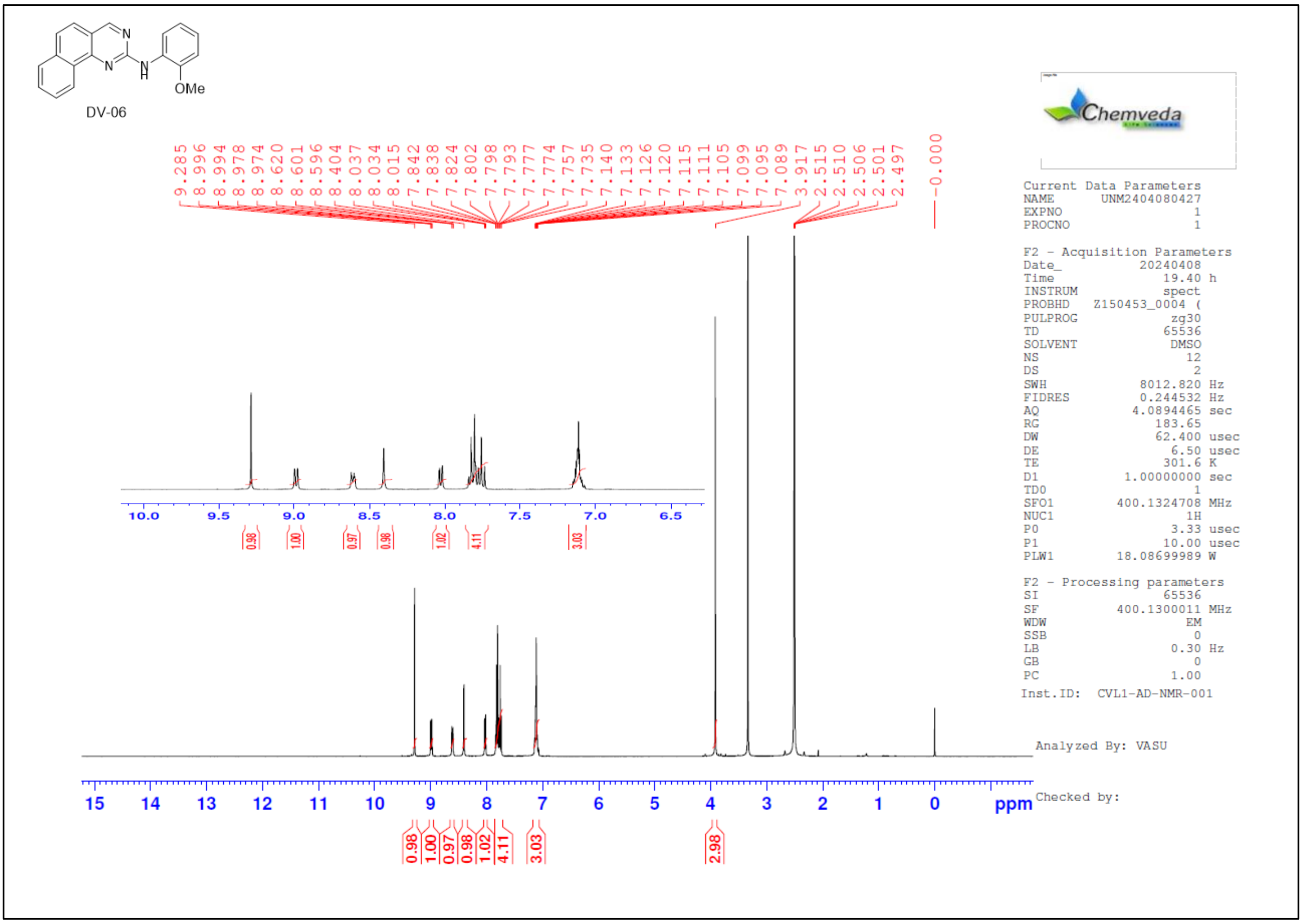

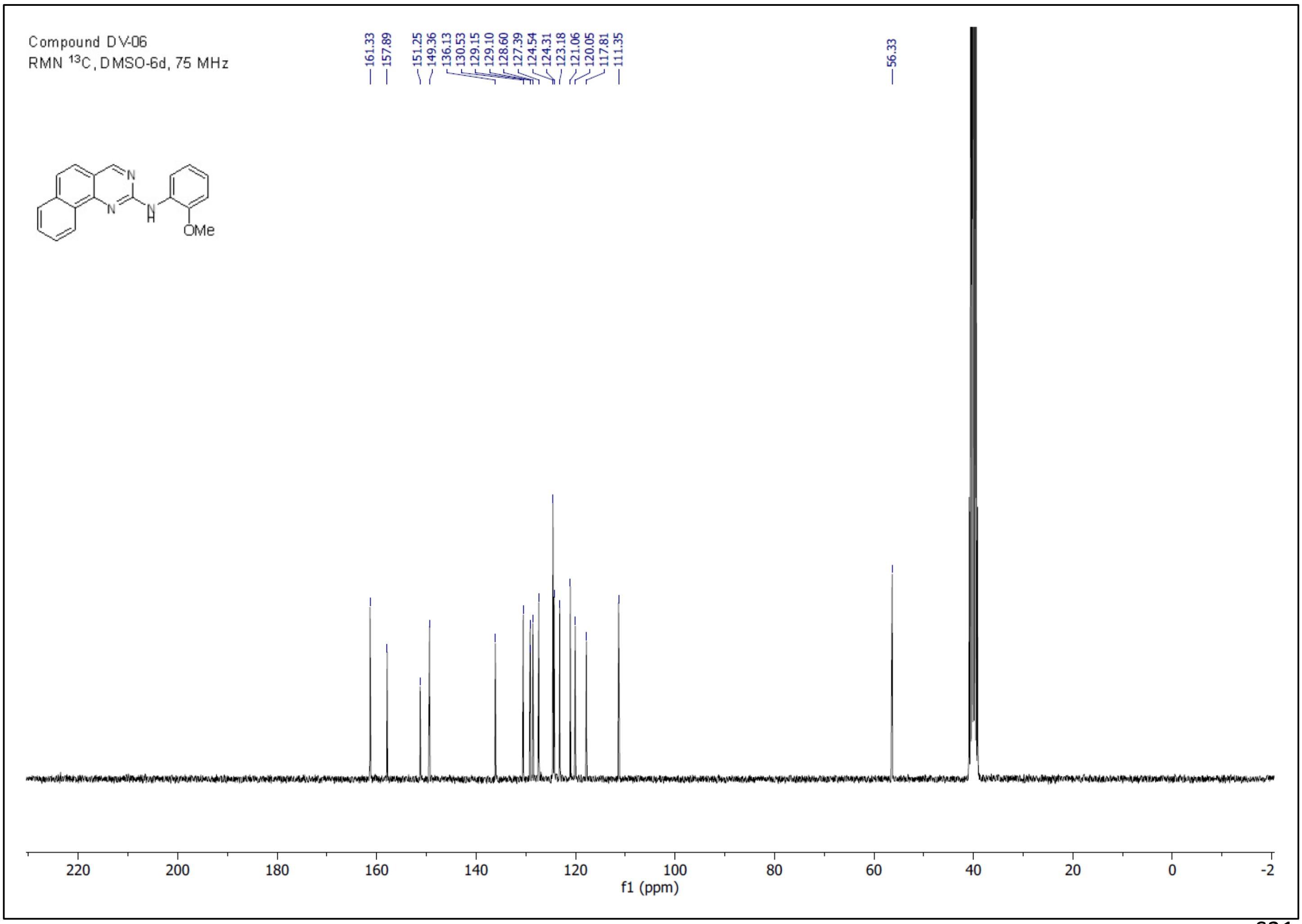

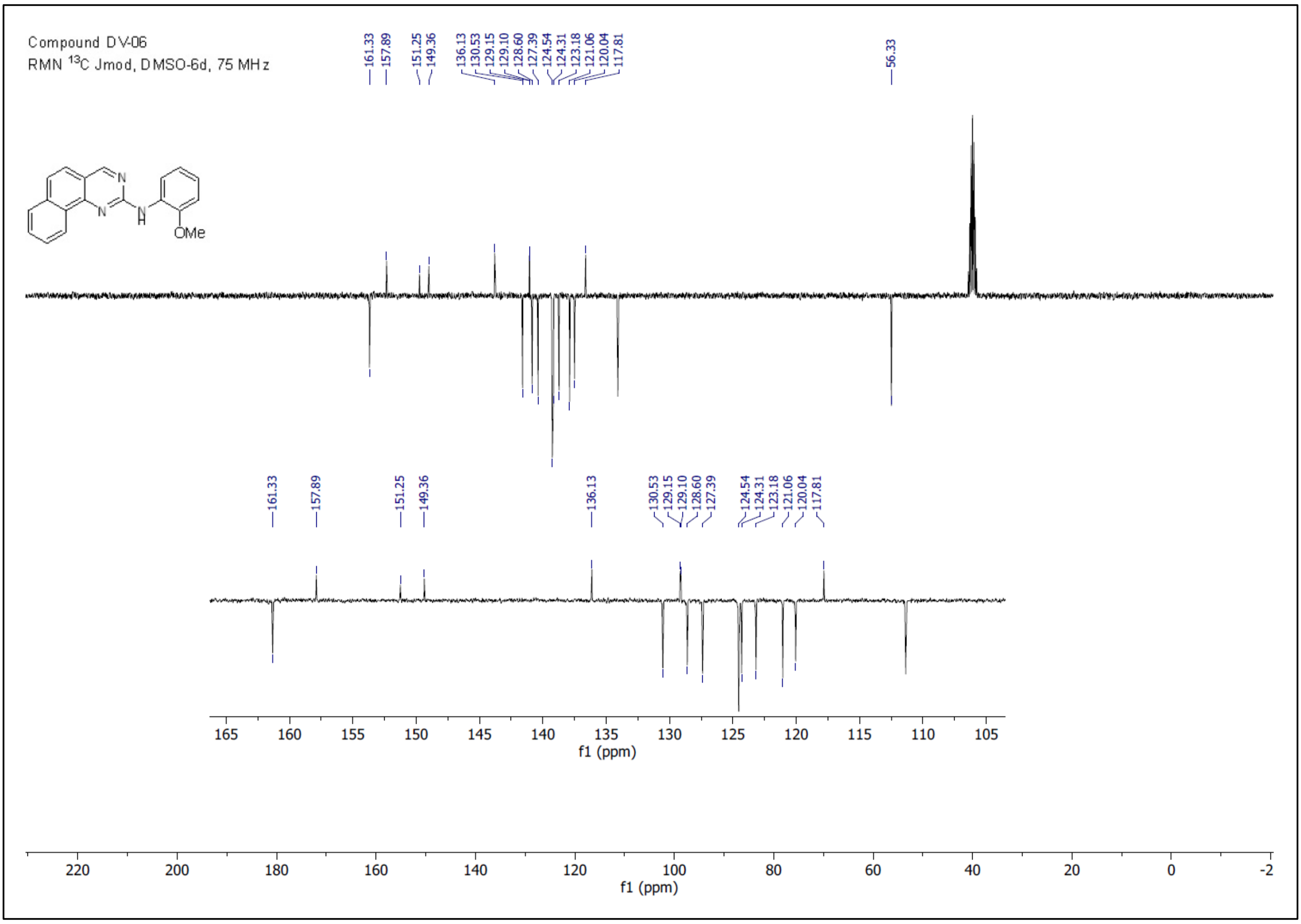

Compound DV-06
RMN 13C Jmod, DMSO-6d, 75 MHz
161.33
157.89
151.25
149.36
136.13
130.53
129.15
129.10
128.60
127.39
124.54
124.31
123.18
121.06
120.04
117.81
56.33
N
N
N
H
OMe
f1 (ppm)

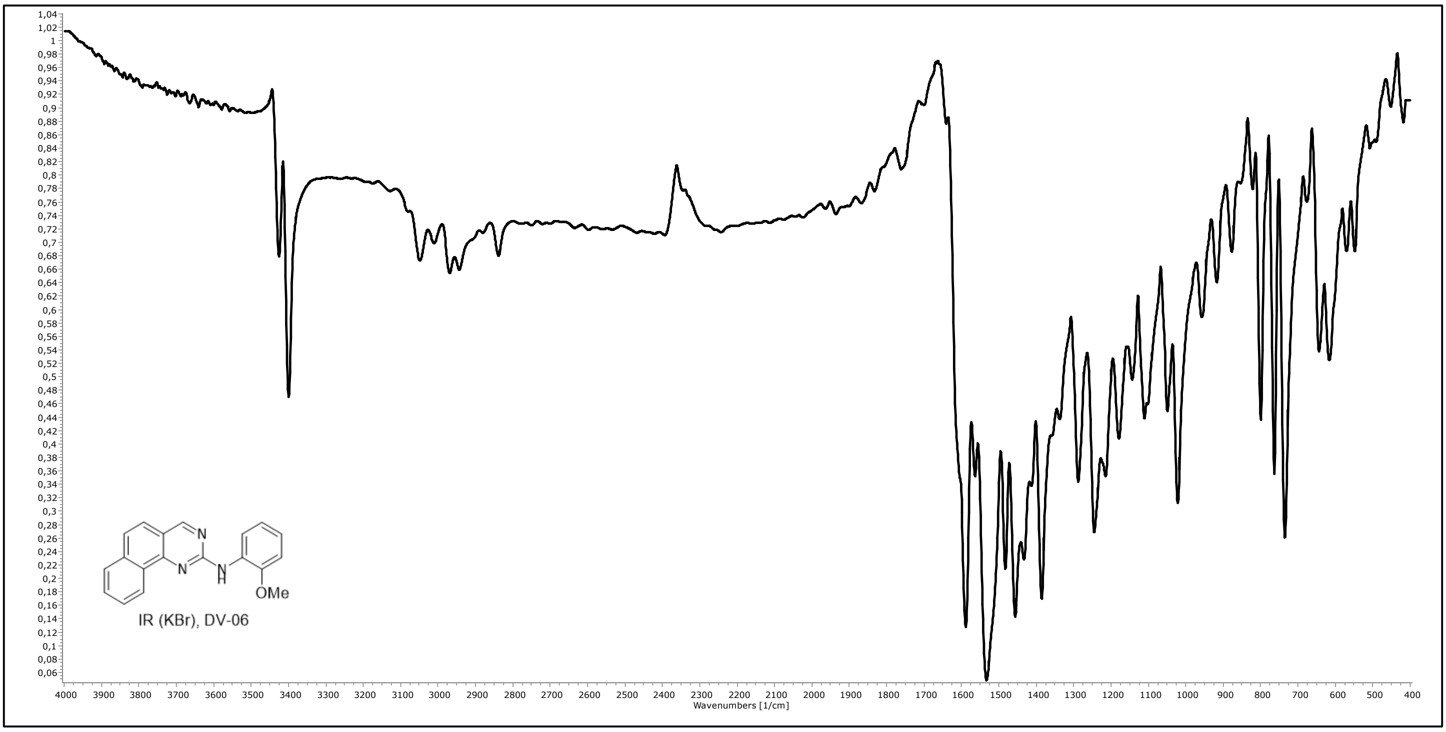

N
N
N
H
OMe
IR (KBr), DV-06
Wavenumbers [1/cm]

Spectra: ^{1}H, ^{13}C, ^{13}C Jmod NMR and FT-IR data of compound 4f (DV-07)

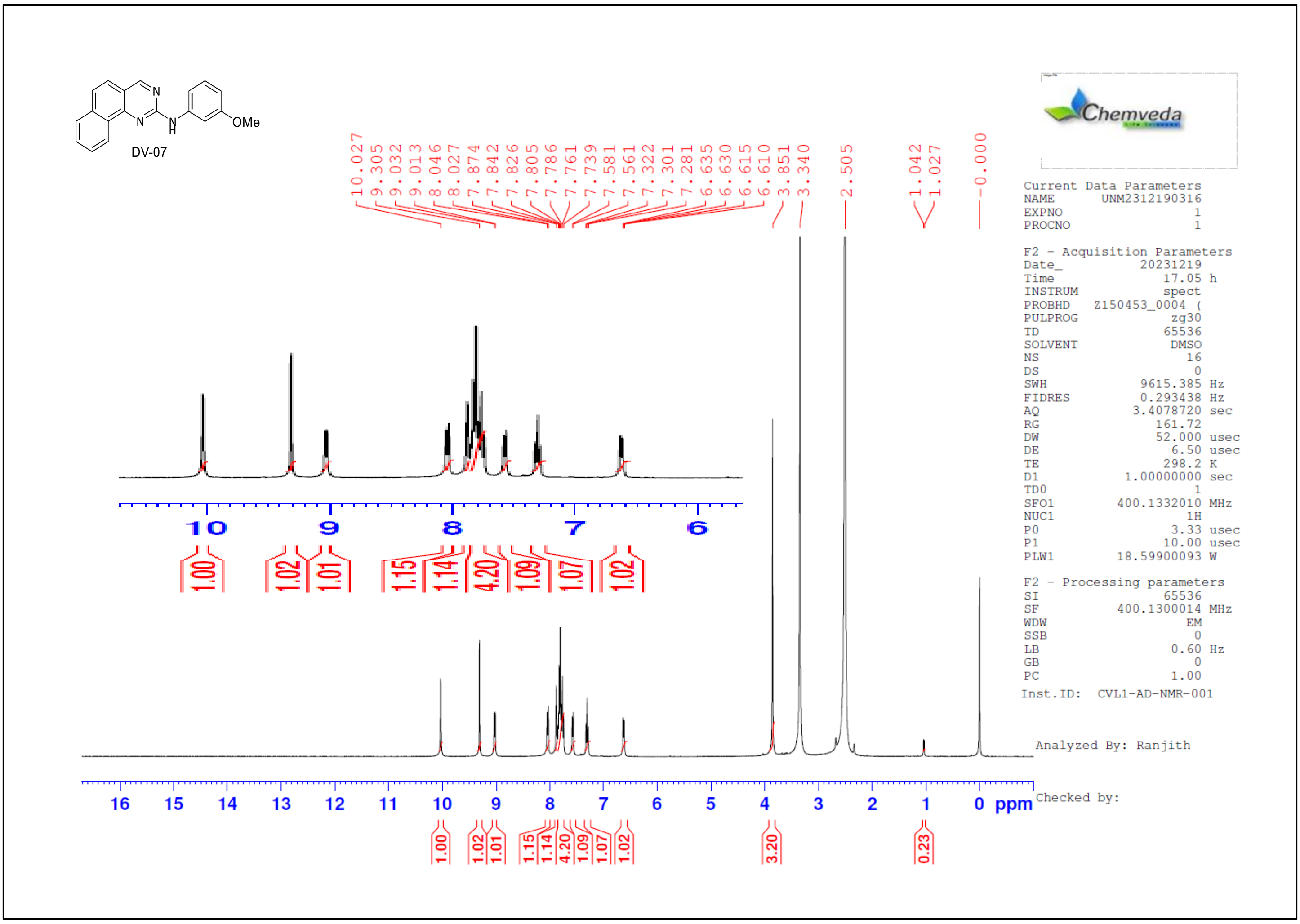

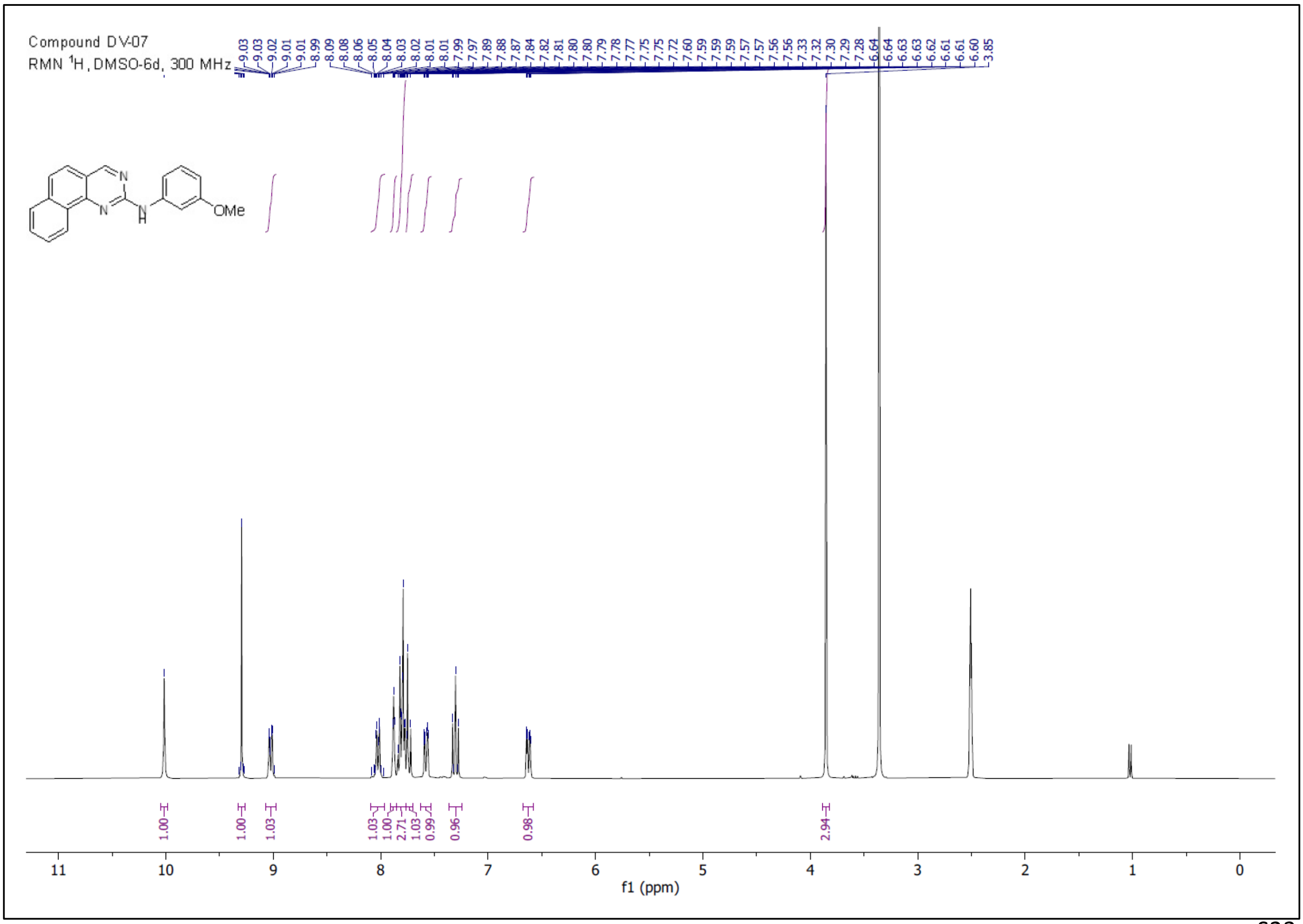

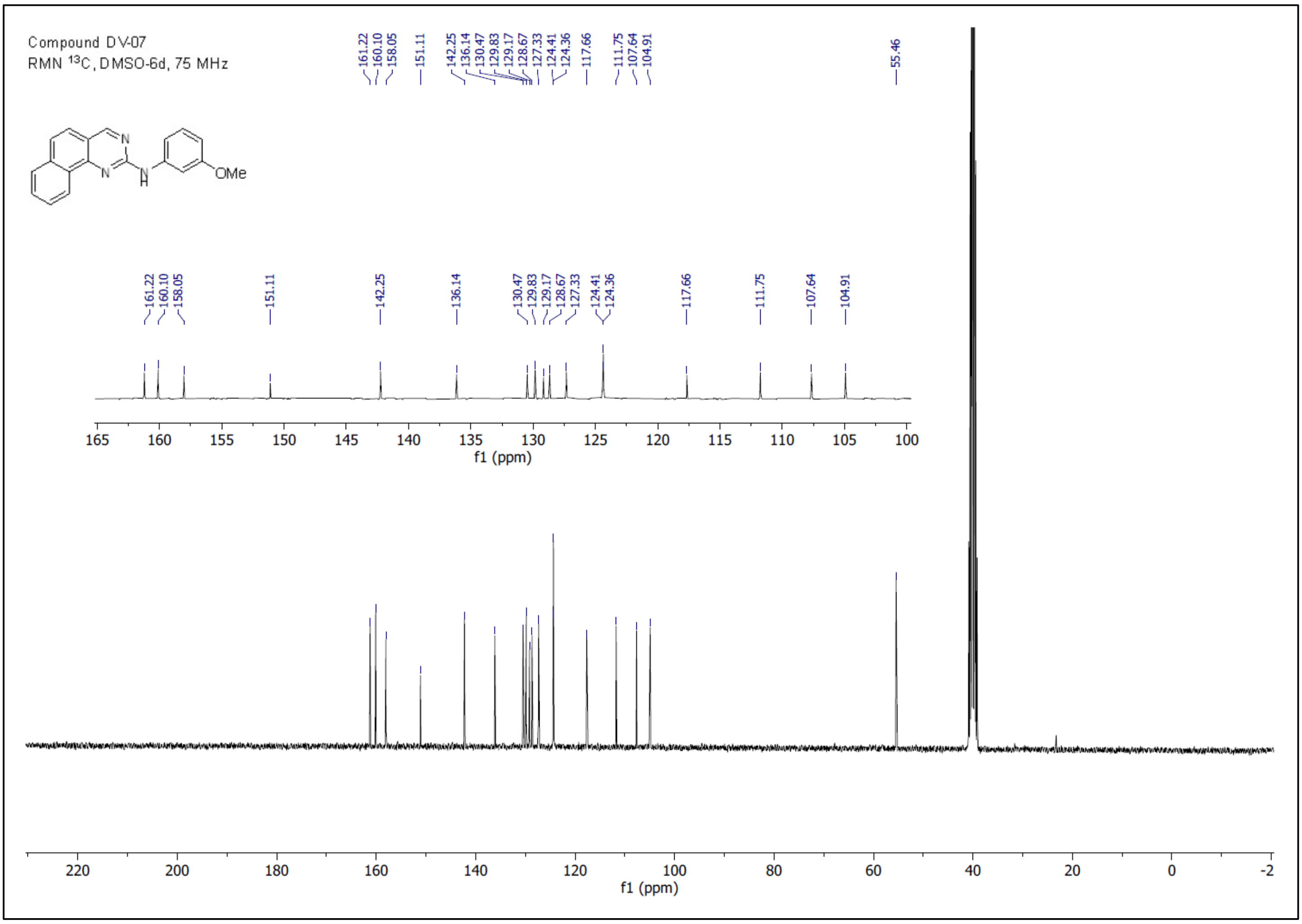
Compound DV-07
RMN 13C, DMSO-6d, 75 MHz
OMe
N
N
H
161.22
160.10
158.05
151.11
142.25
136.14
130.47
129.83
129.17
128.67
127.33
124.41
124.36
117.66
111.75
107.64
104.91
55.46
f1 (ppm)

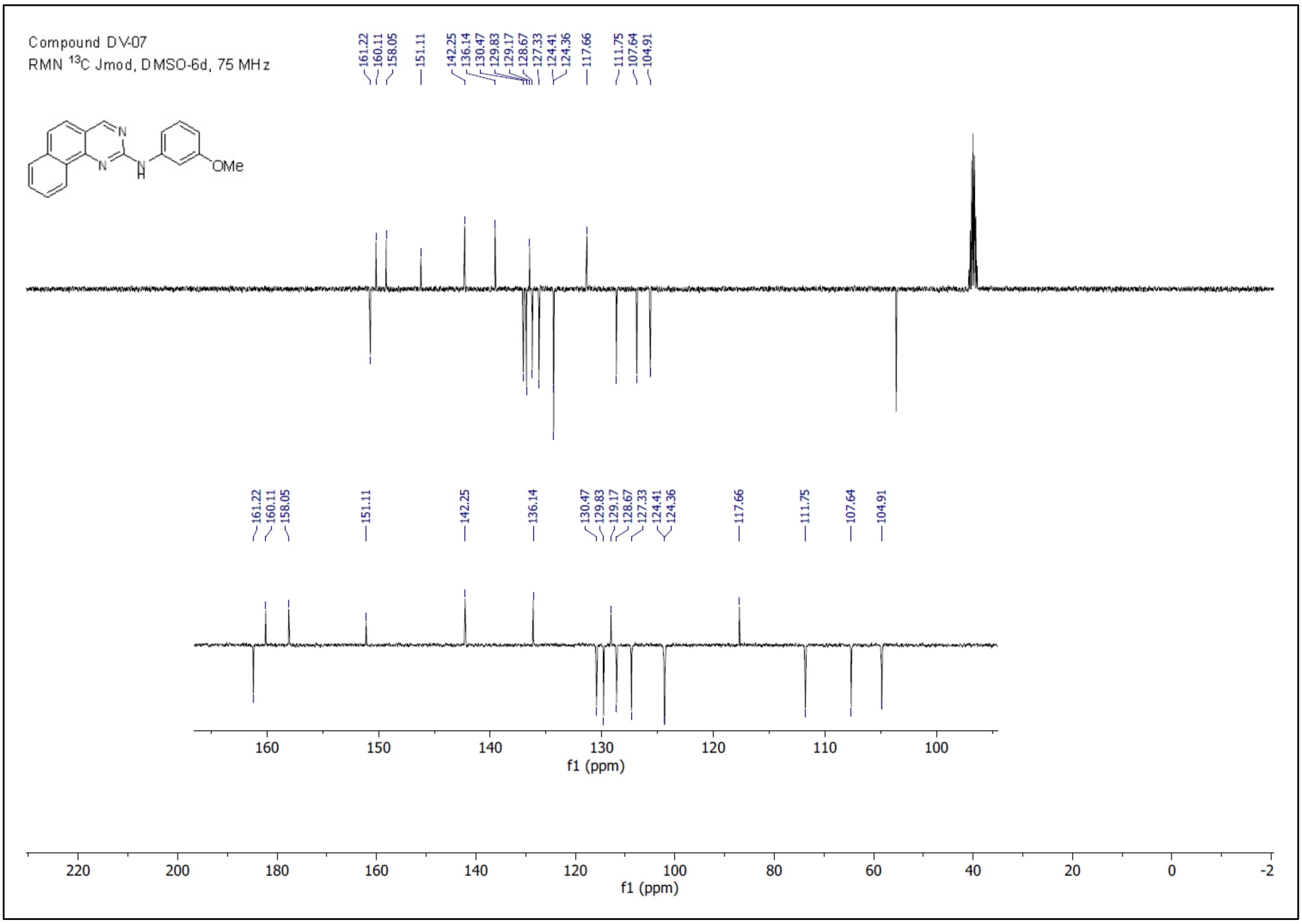
Compound DV-07
RMN 13C Jmod, DMSO-6d, 75 MHz
OMe
N
N
H
161.22
160.11
158.05
151.11
142.25
136.14
130.47
129.83
129.17
128.67
127.33
124.41
124.36
117.66
111.75
107.64
104.91
f1 (ppm)

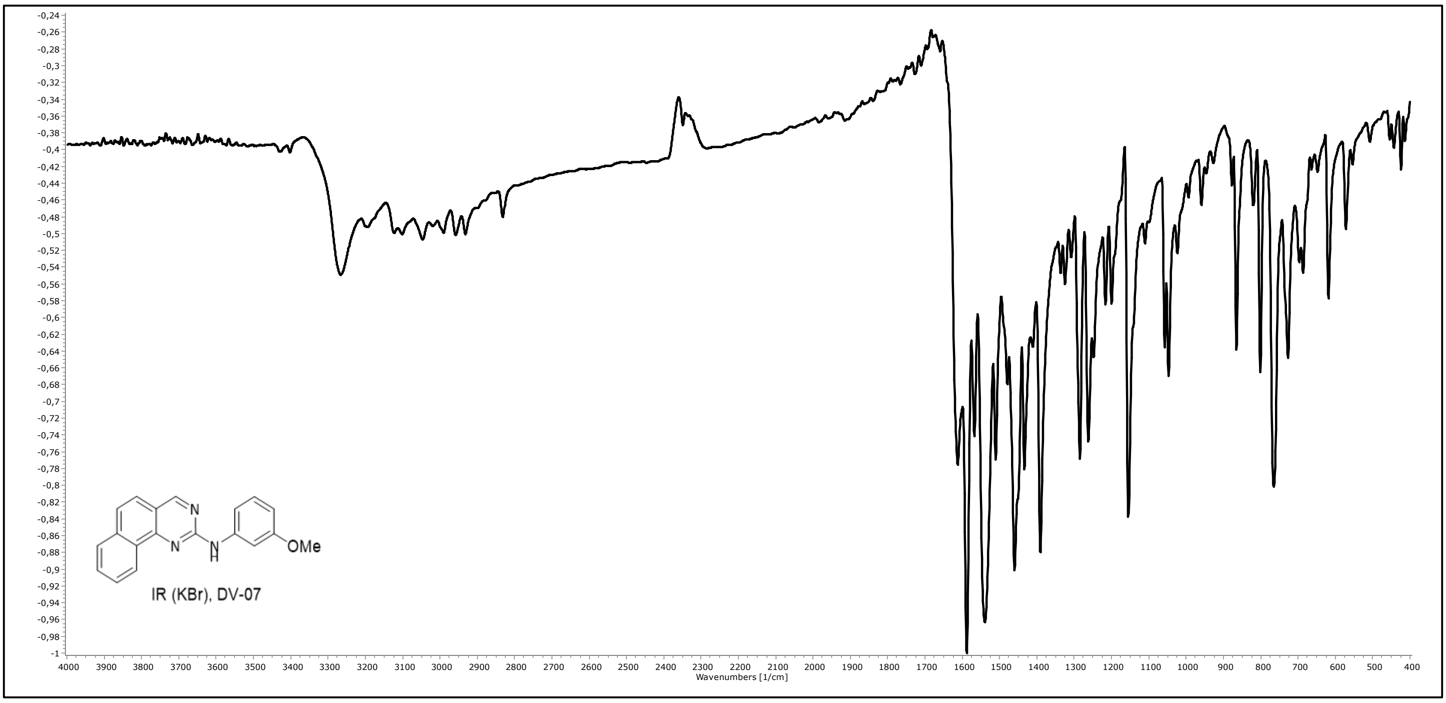

N
N
N
H
OMe
IR (KBr), DV-07
Wavenumbers [1/cm]

Spectra: ^{1}H, ^{13}C, ^{13}C Jmod NMR and FT-IR data of compound 4g (DV-08)

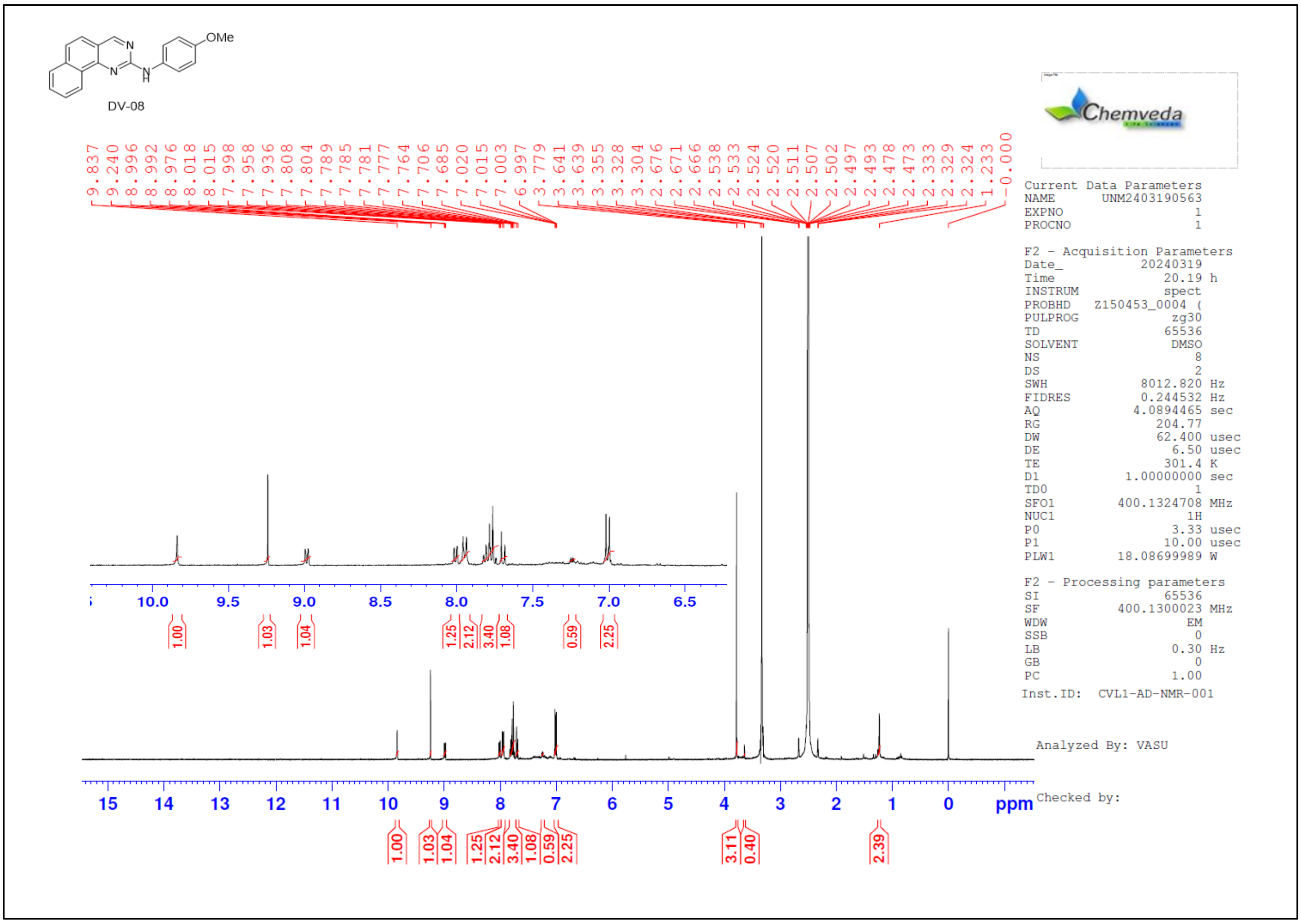

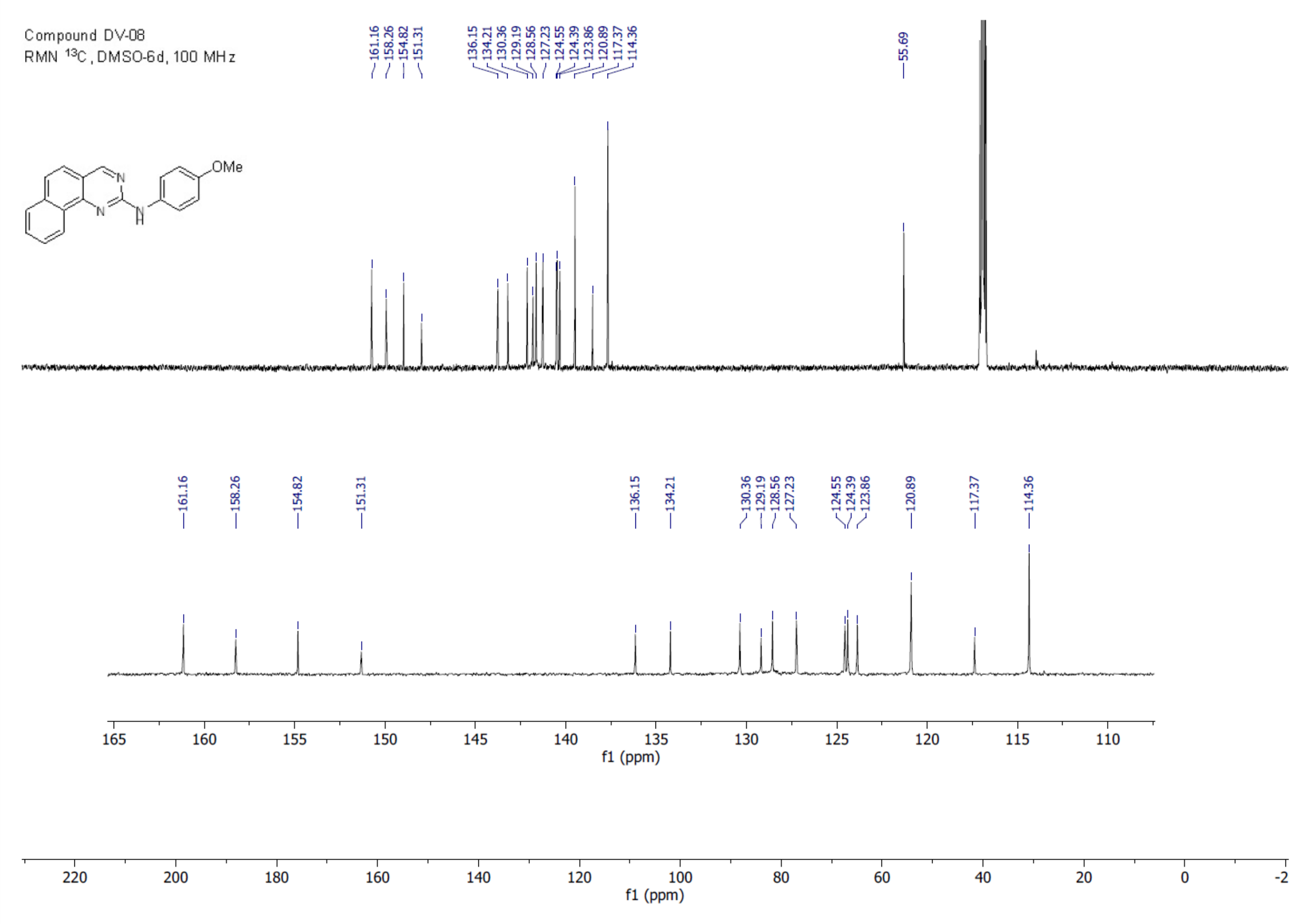

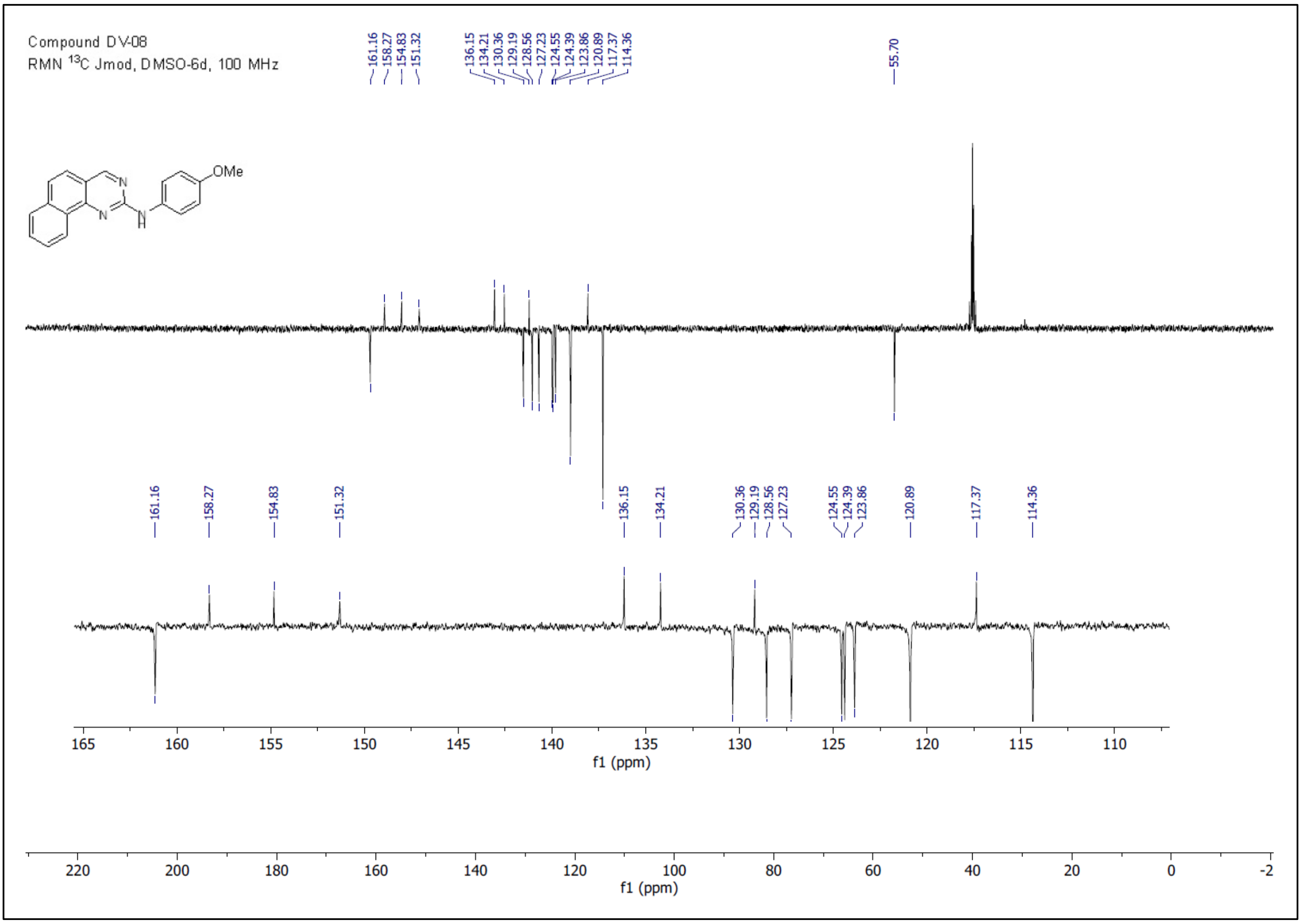
Compound DV-08
RMN 13C Jmod, DMSO-6d, 100 MHz
OMe
N
N
H
161.16
158.27
154.83
151.32
136.15
134.21
130.36
129.19
128.56
127.23
124.55
124.39
123.86
120.89
117.37
114.36
55.70
f1 (ppm)

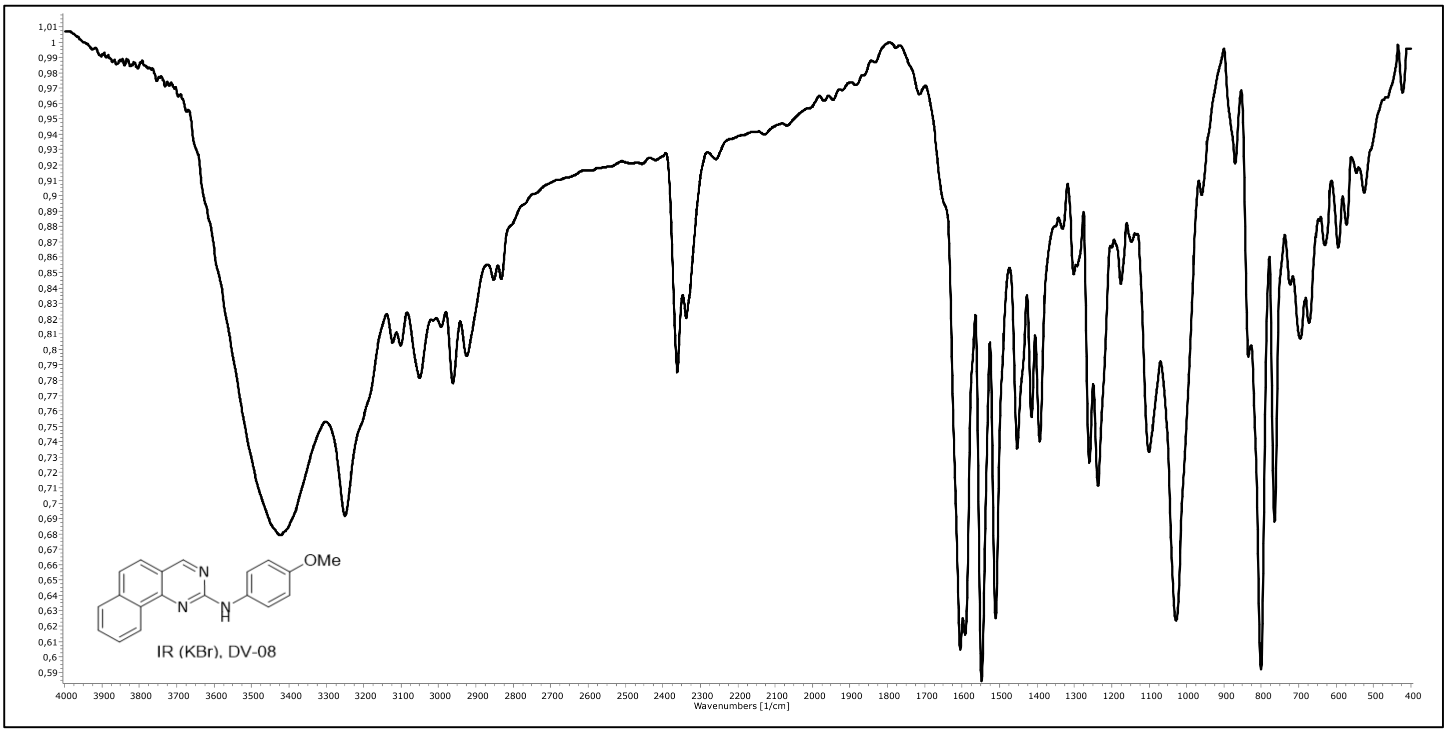
OMe
IR (KBr), DV-08
Wavenumbers [1/cm]

Spectra: ^{1}H, ^{13}C, ^{13}C Jmod NMR and FT-IR data of compound 4h (DV-09)

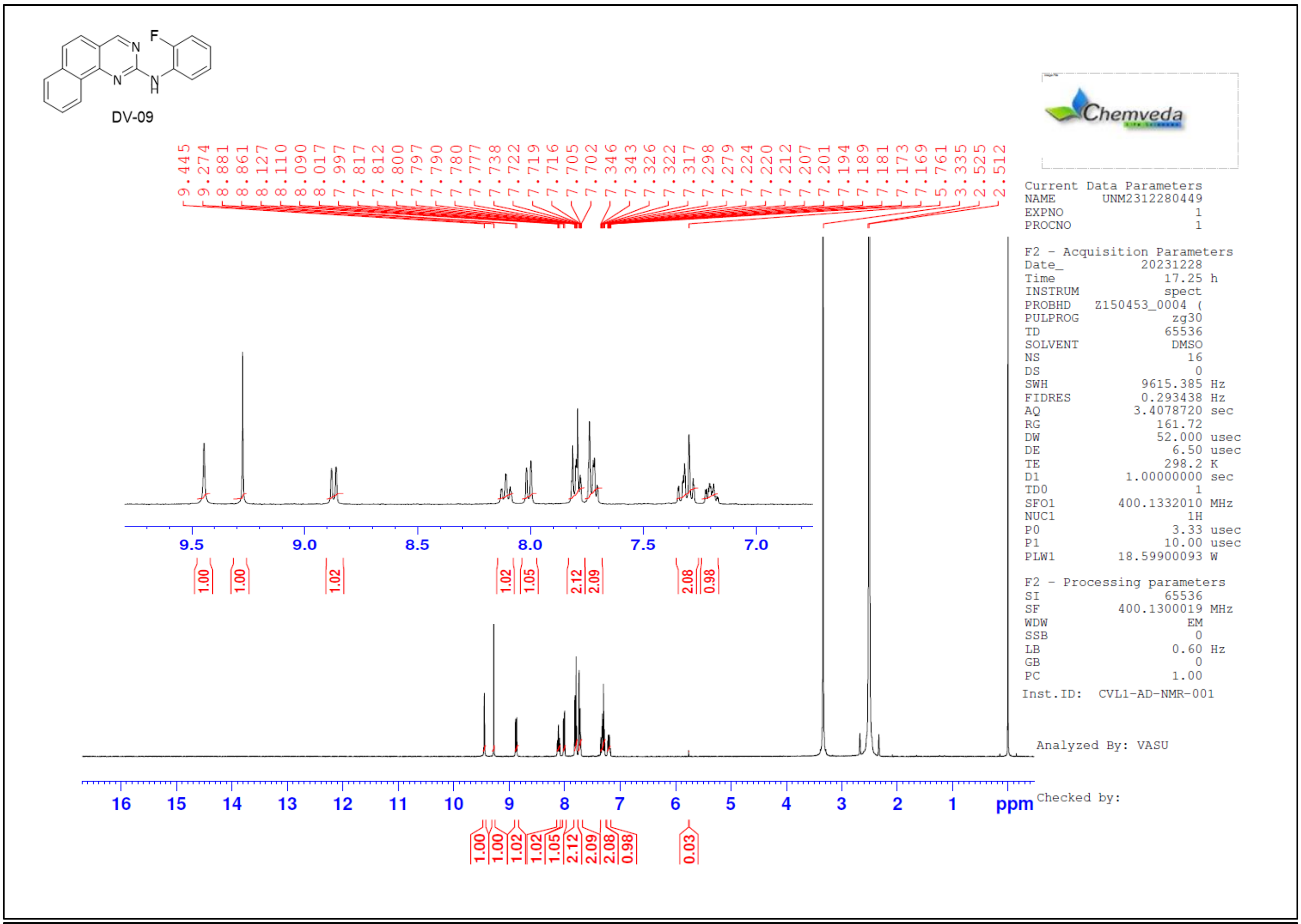

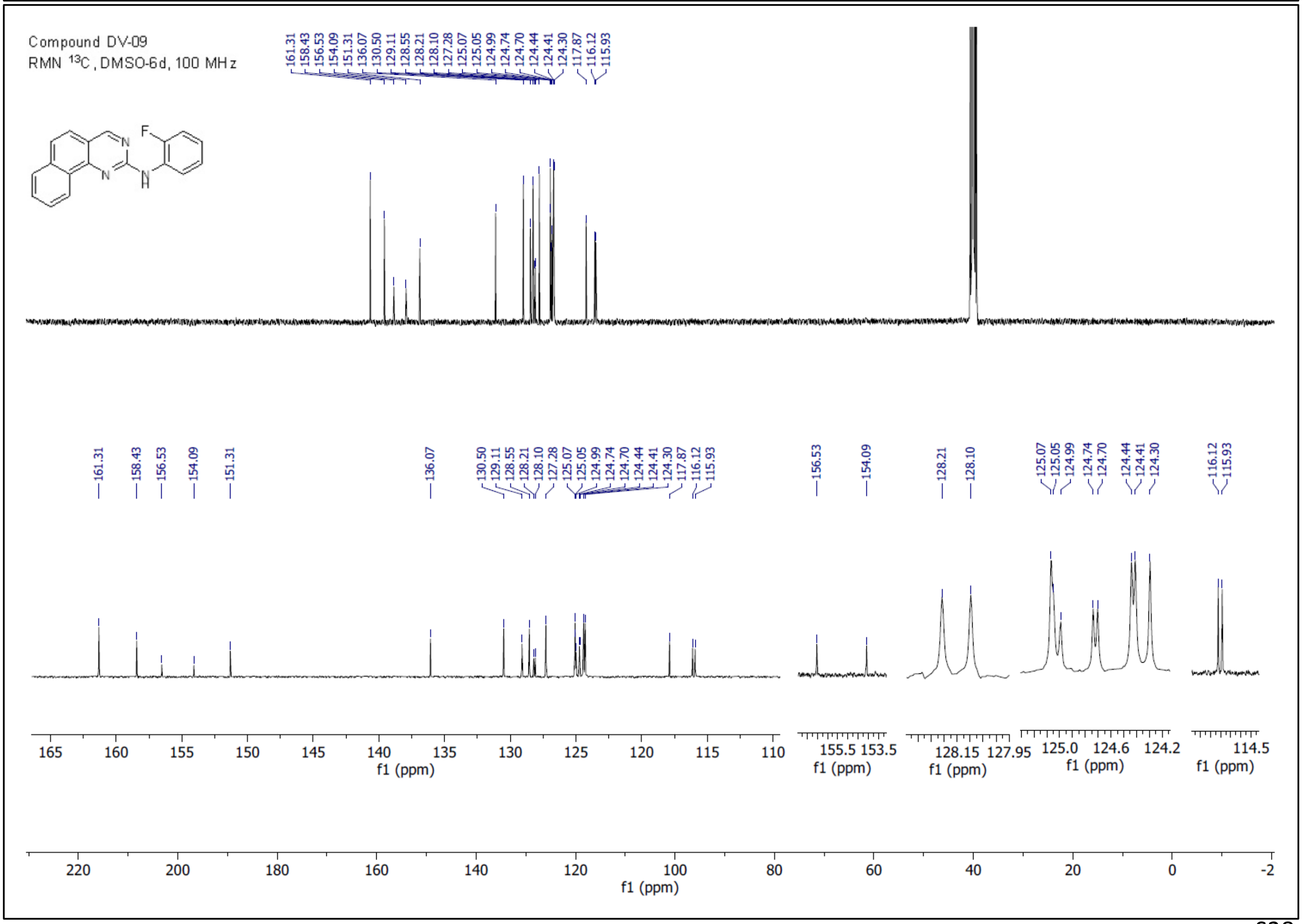

Compound DV-09
RMN ^{13}C Jmod, DMSO-6d, 100 MHz

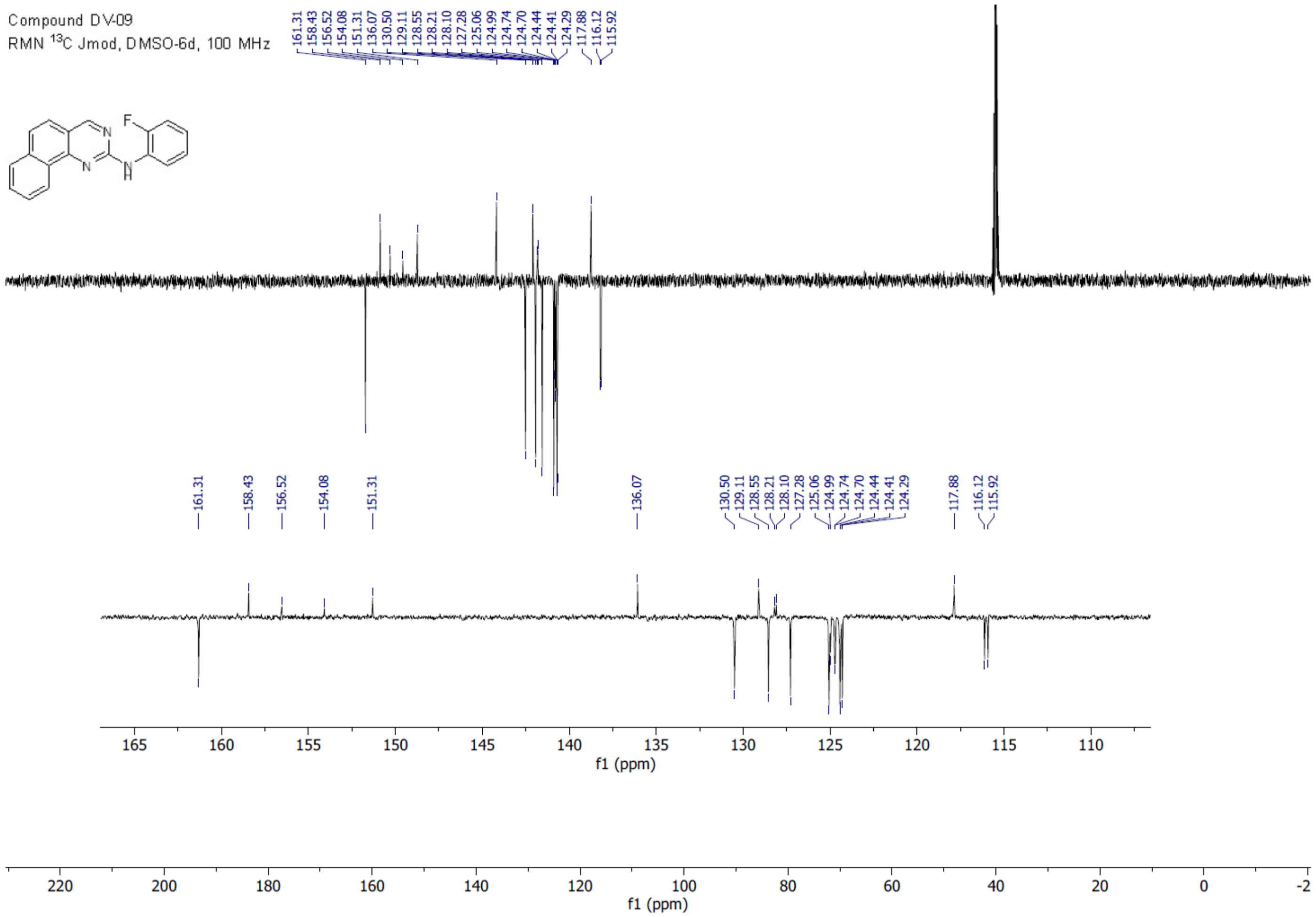

Compound DV-09
RMN ^{19}F, DMSO-6d, 282 MHz

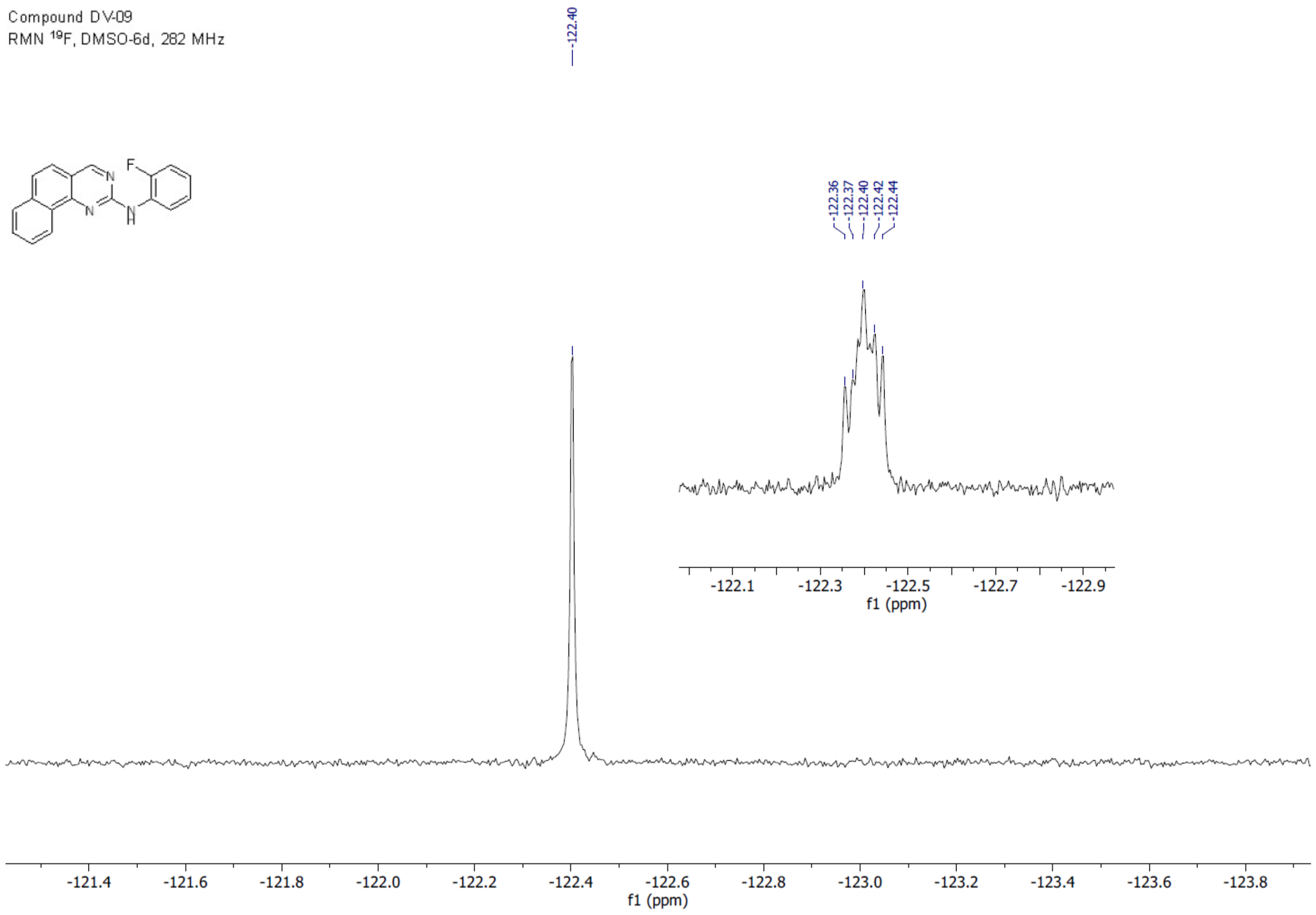

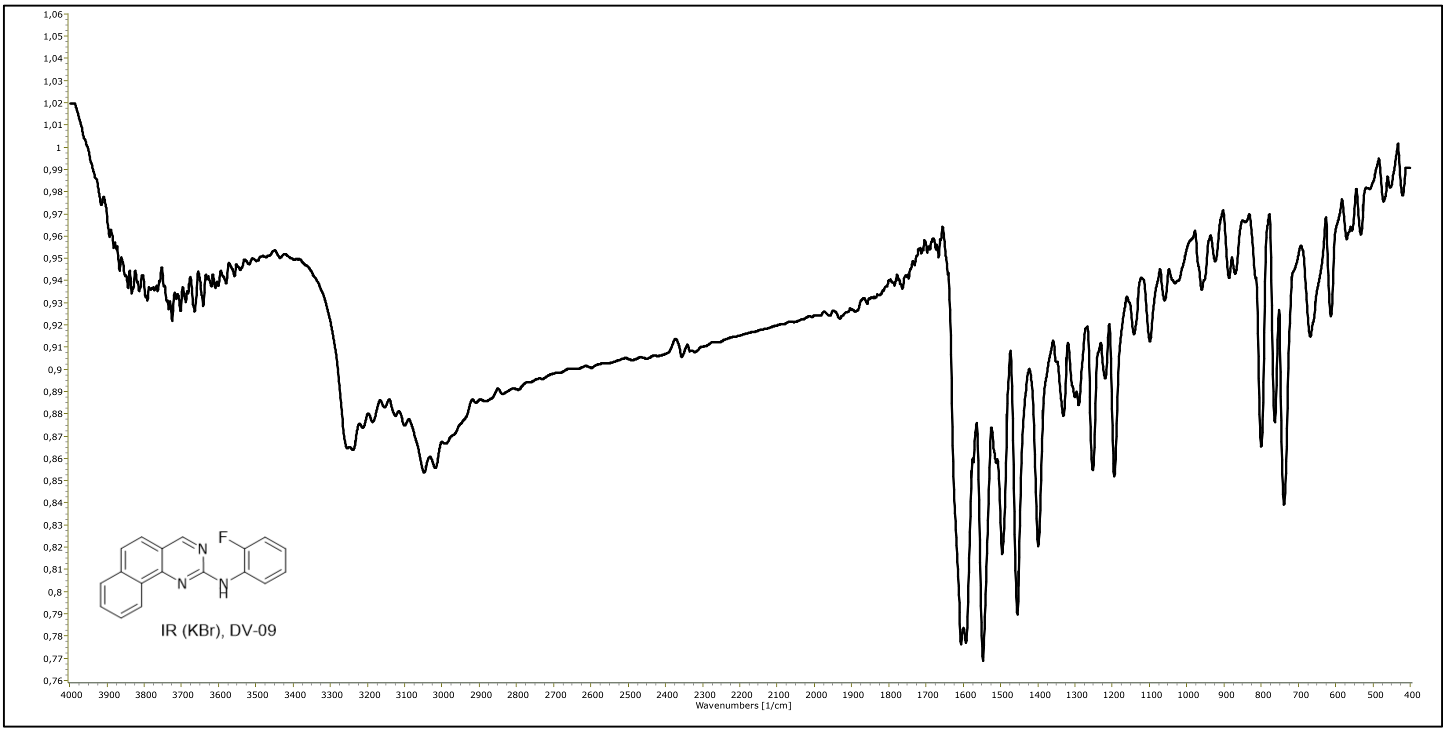
IR (KBr), DV-09
Wavenumbers [1/cm]

Spectra: ^{1}H, ^{13}C, ^{13}C Jmod NMR and FT-IR data of compound 4i (DV-10)

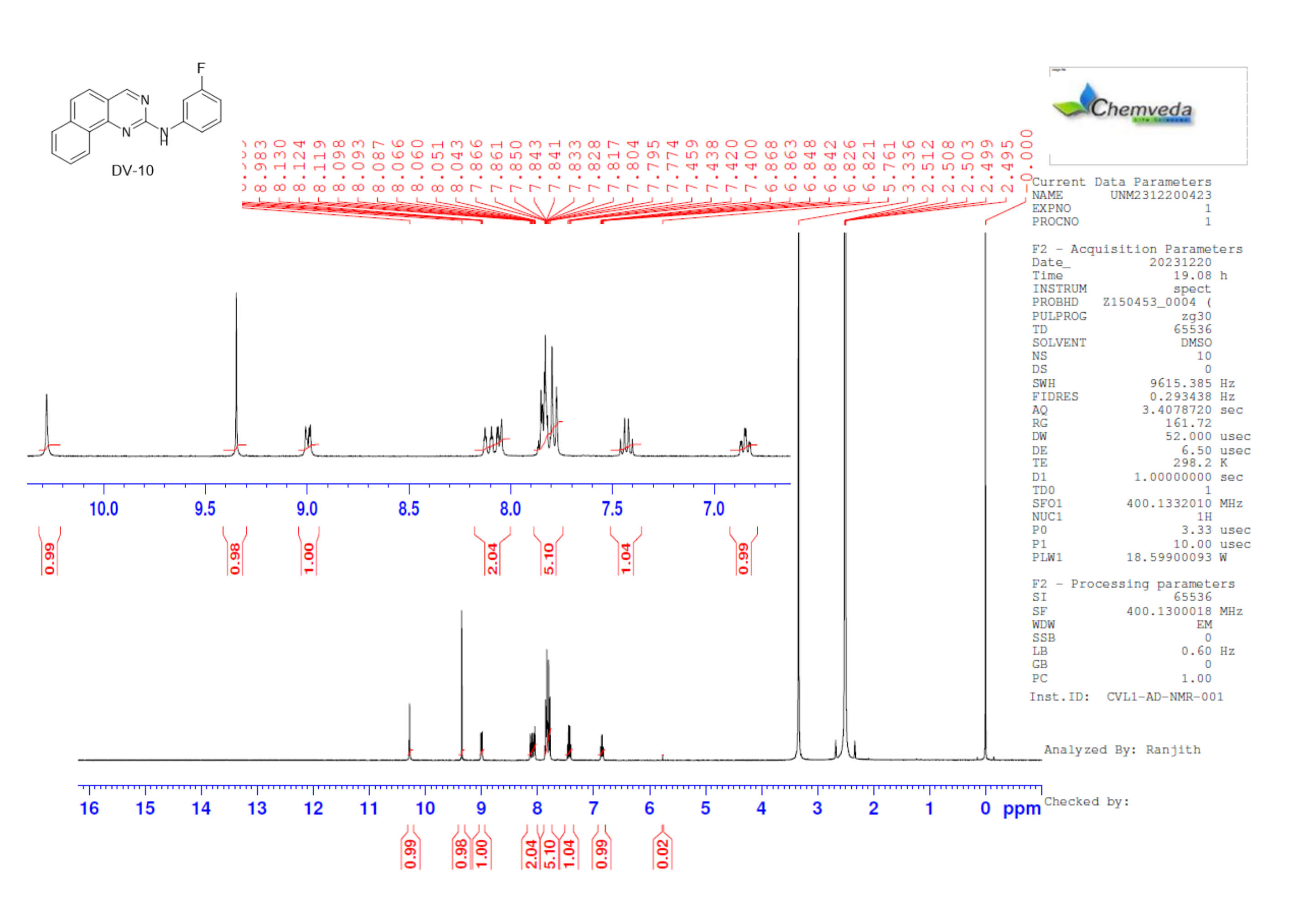

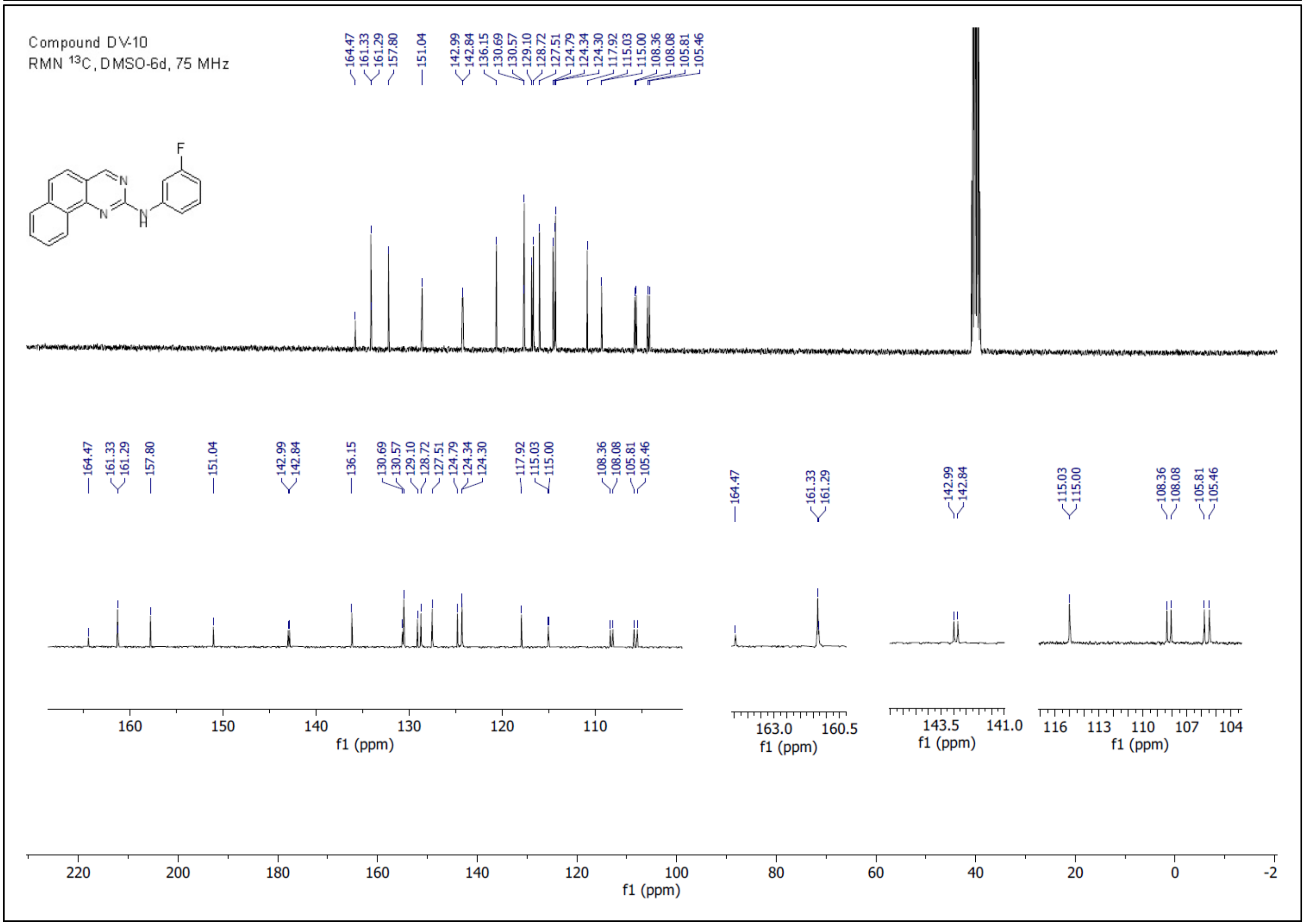

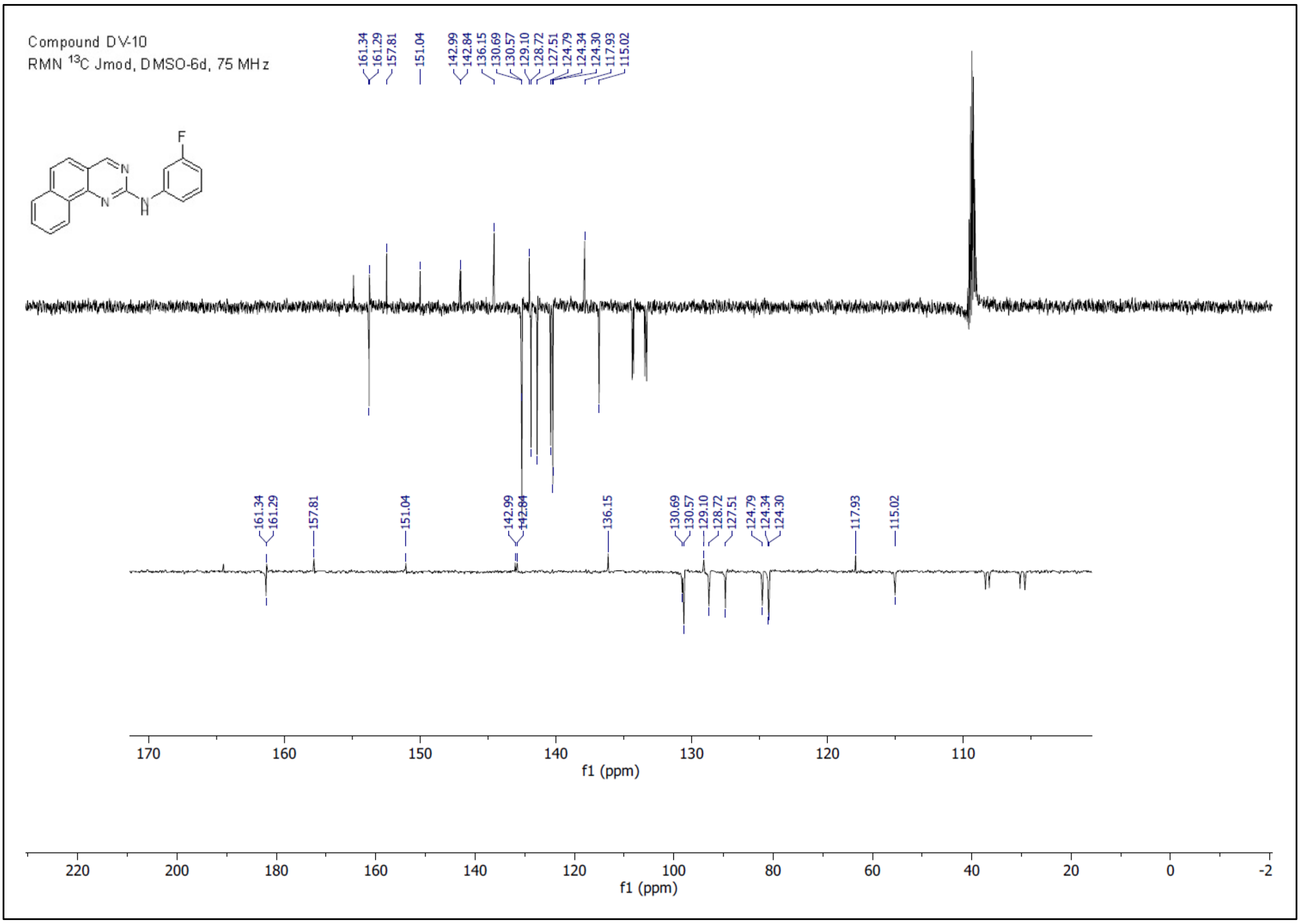
Compound DV-10
RMN 13C Jmod, DMSO-6d, 75 MHz
161.34
161.29
157.81
151.04
142.99
142.84
136.15
130.69
130.57
129.10
128.72
127.51
124.79
124.34
124.30
117.93
115.02
F
N
N
N
H
f1 (ppm)

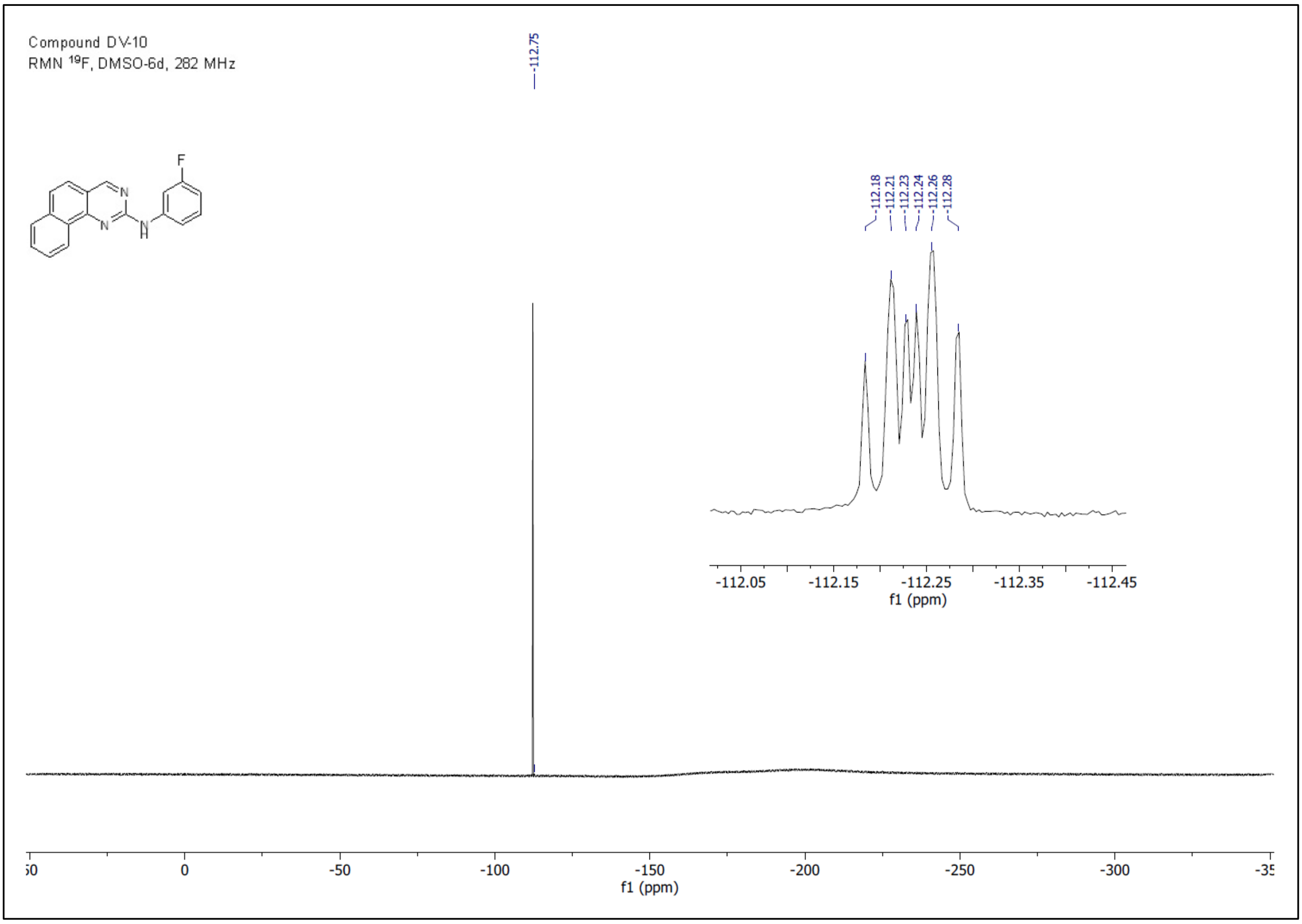
Compound DV-10
RMN 19F, DMSO-6d, 282 MHz
-112.75
-112.18
-112.21
-112.23
-112.24
-112.26
-112.28
F
N
N
N
H
f1 (ppm)

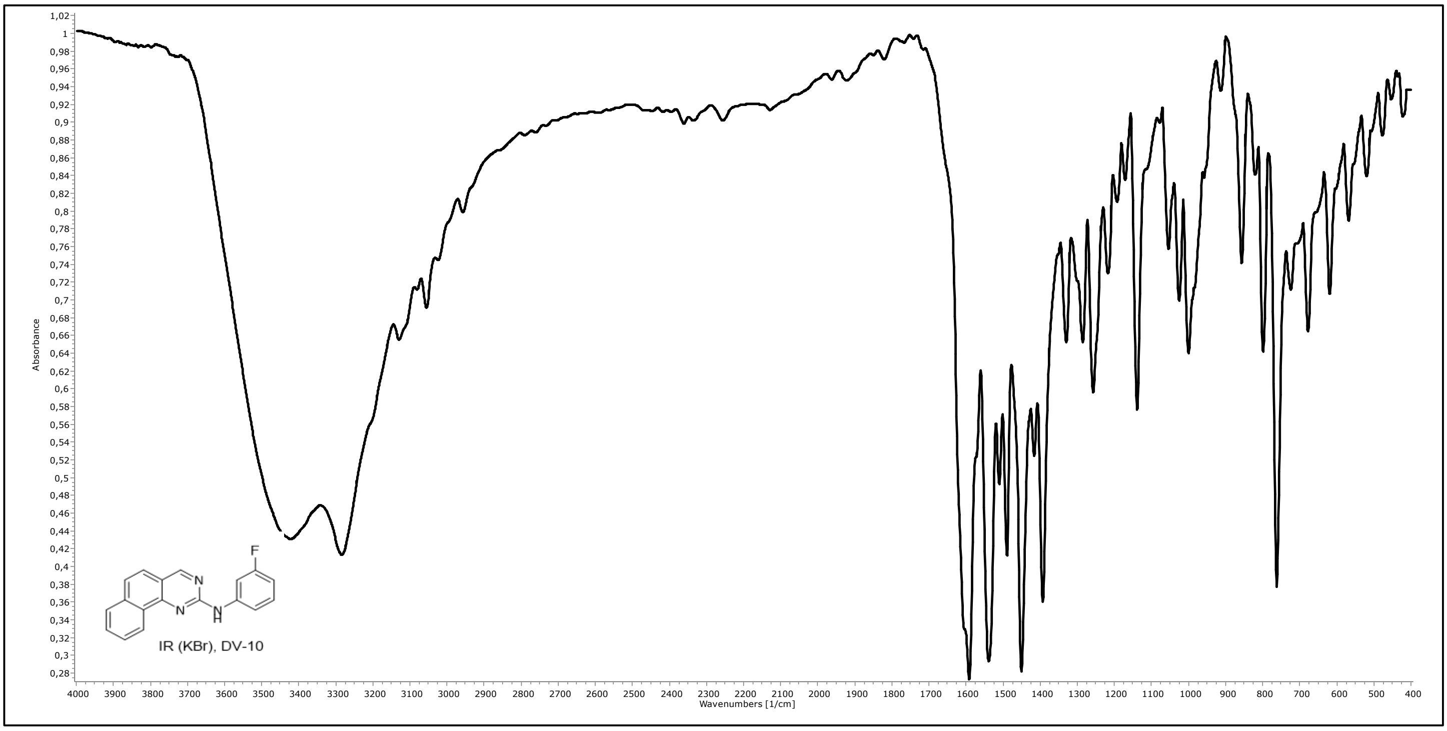
Absorbance
Wavenumbers [1/cm]
IR (KBr), DV-10
F
N
N
H

Spectra: ^{1}H, ^{13}C, ^{13}C Jmod NMR and FT-IR data of compound 4j (DV-11)

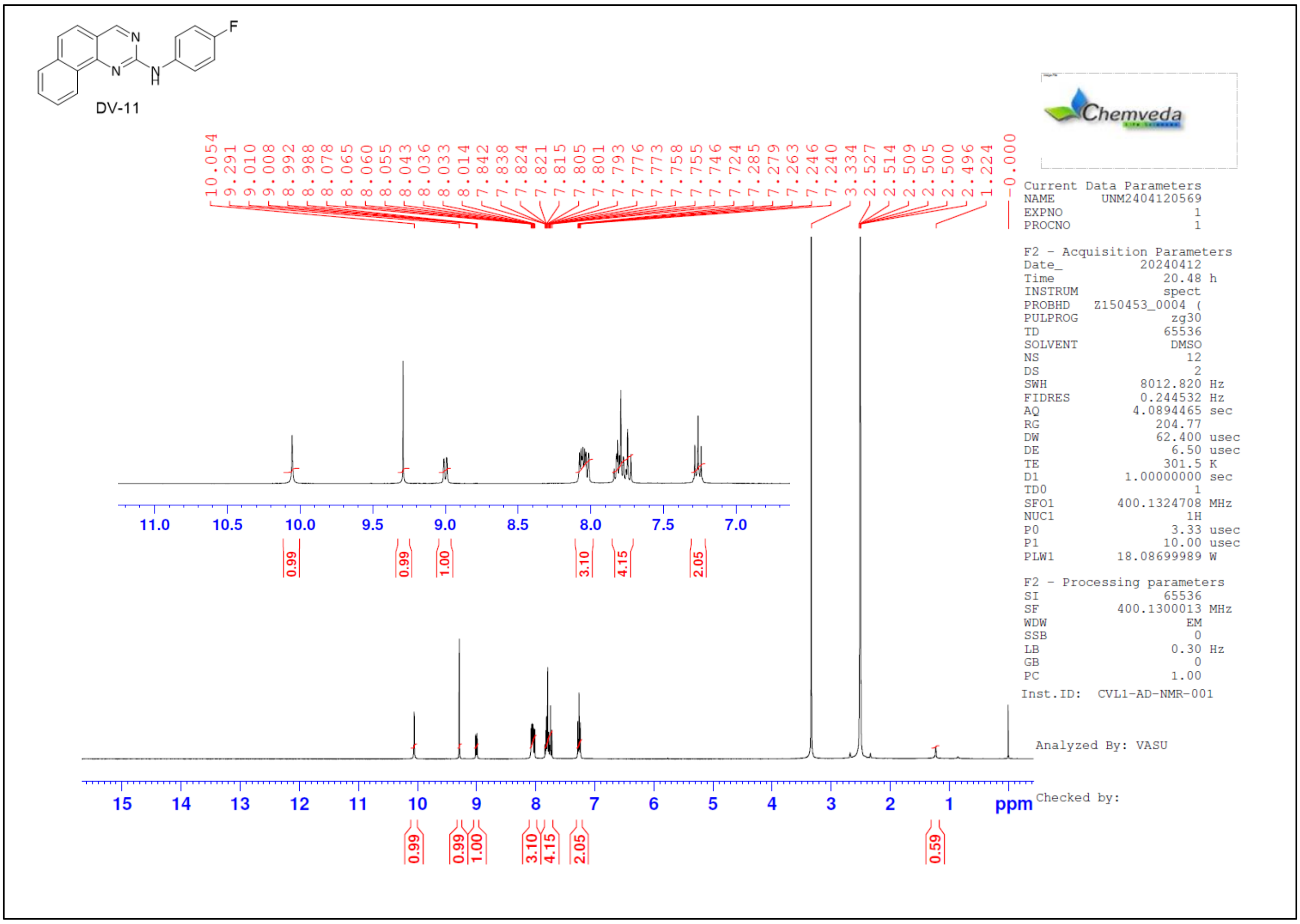

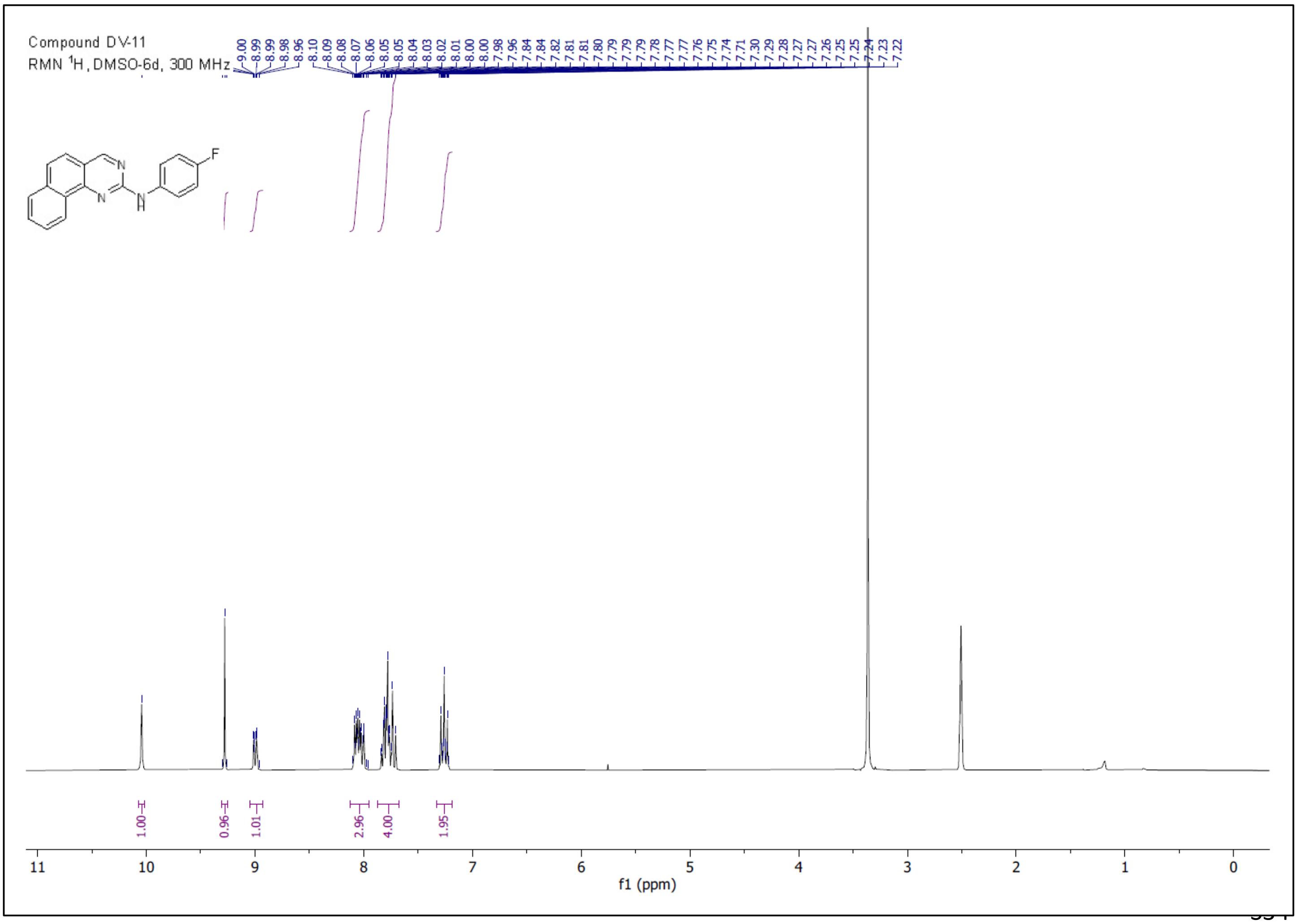

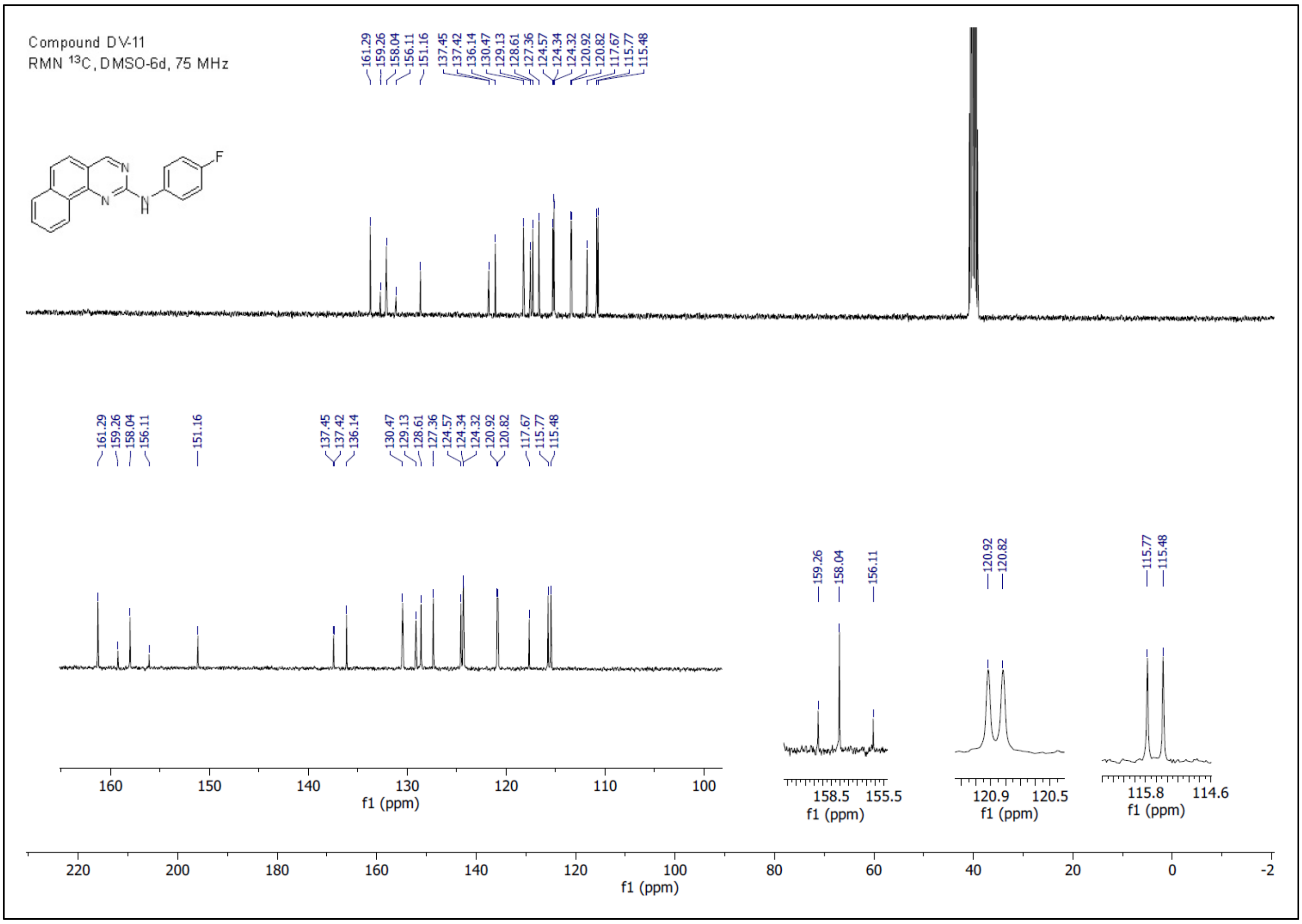
Compound DV-11
RMN 13C, DMSO-6d, 75 MHz
161.29 159.26 158.04 156.11 151.16 137.45 137.42 136.14 130.47 129.13 128.61 127.36 124.57 124.34 124.32 120.92 120.82 117.67 115.77 115.48
F
N
N
H
f1 (ppm)
158.5 155.5
120.9 120.5
115.8 114.6

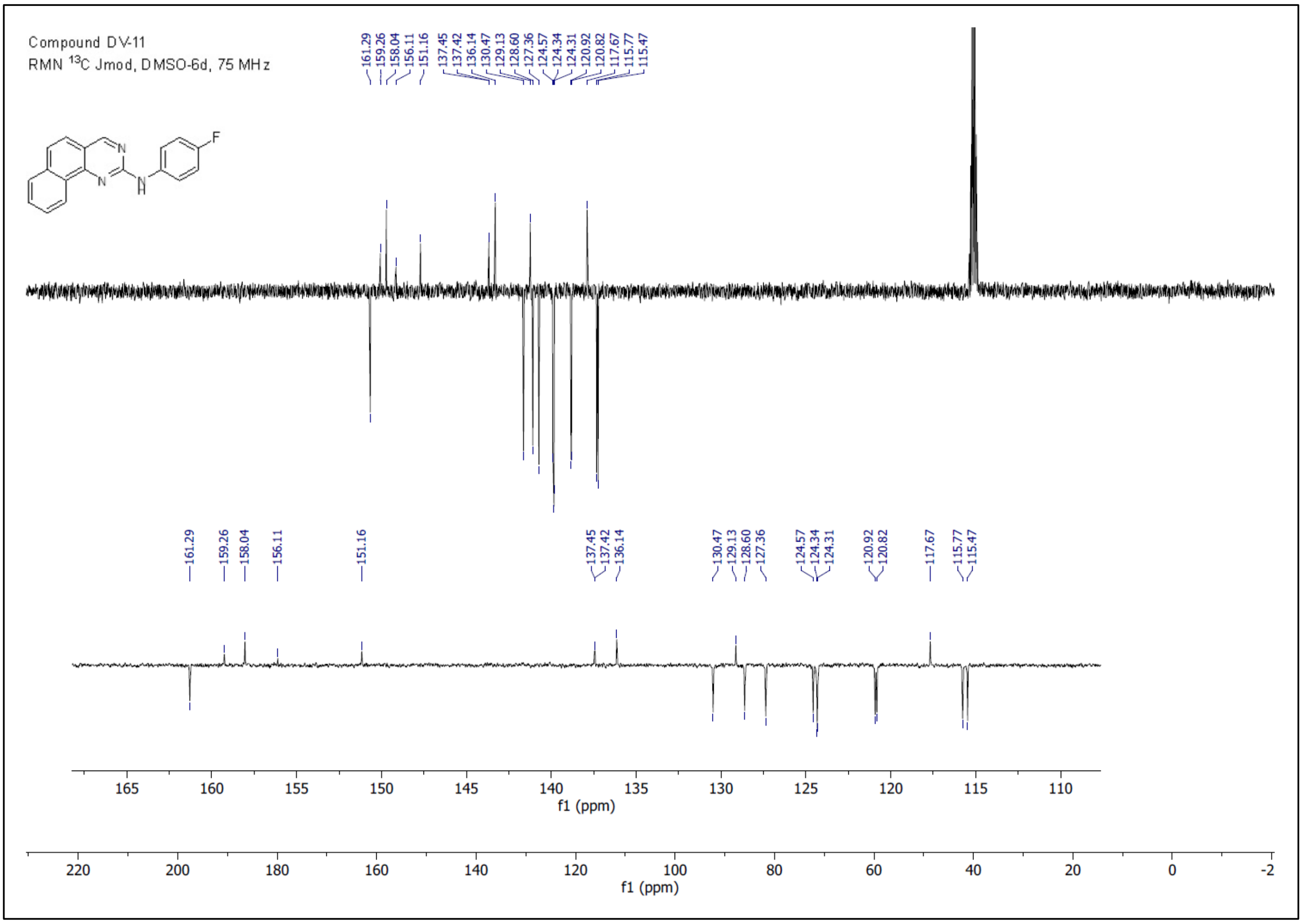
Compound DV-11
RMN 13C Jmod, DMSO-6d, 75 MHz
161.29 159.26 158.04 156.11 151.16 137.45 137.42 136.14 130.47 129.13 128.60 127.36 124.57 124.34 124.31 120.92 120.82 117.67 115.77 115.47
F
N
N
H
f1 (ppm)

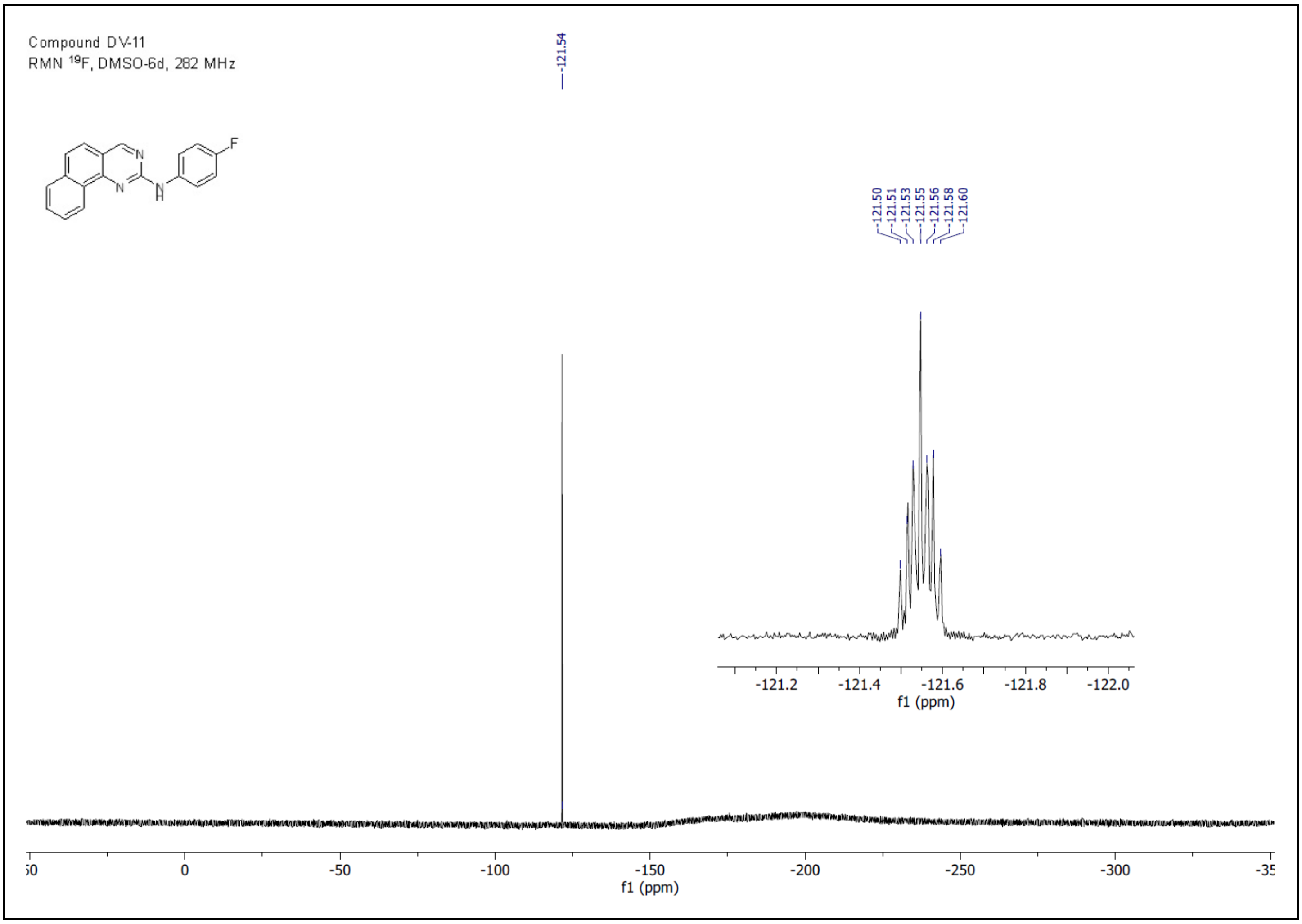
Compound DV-11
RMN 19F, DMSO-6d, 282 MHz
-121.54
-121.50
-121.51
-121.53
-121.55
-121.56
-121.58
-121.60
-121.2
-121.4
-121.6
-121.8
-122.0
f1 (ppm)
0
-50
-100
-150
-200
-250
-300
f1 (ppm)

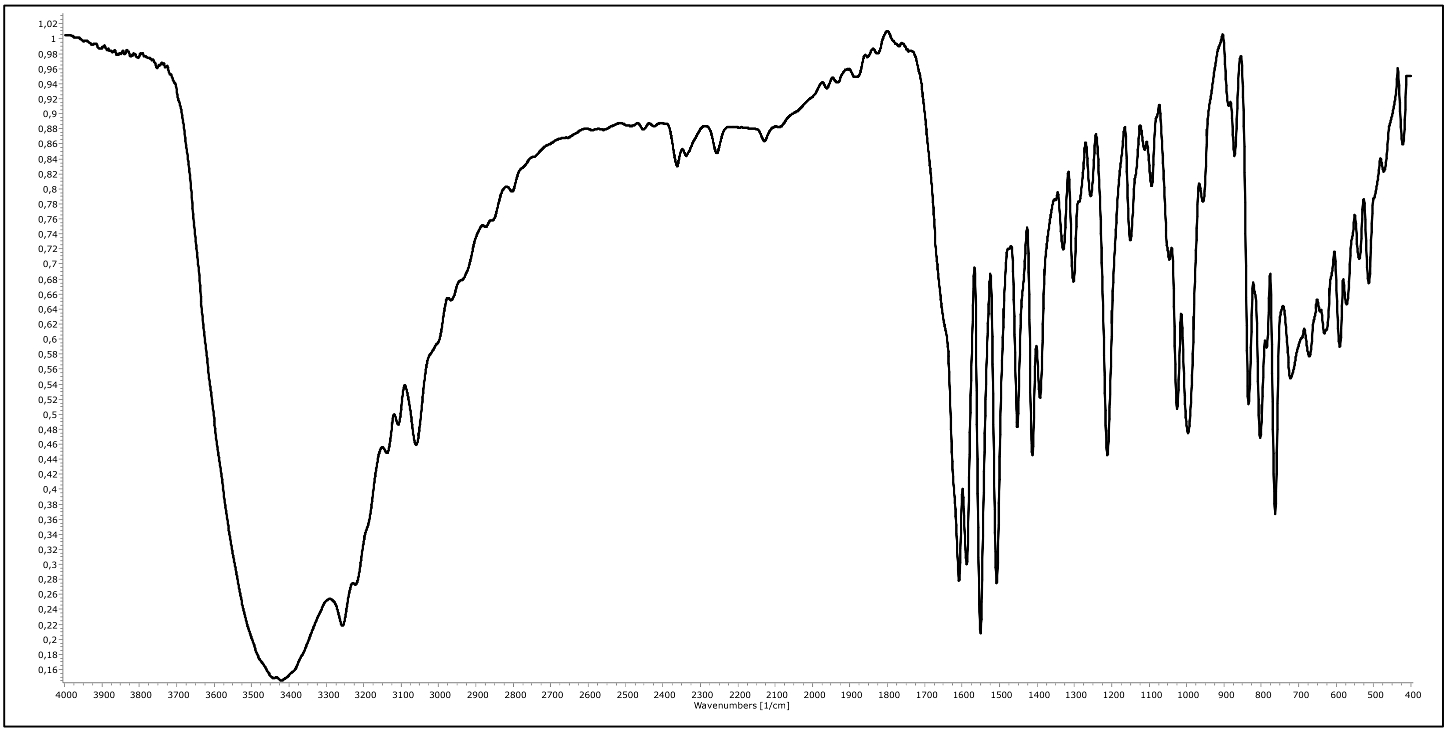
Wavenumbers [1/cm]

Spectra: ^{1}H, ^{13}C, ^{13}C Jmod NMR and FT-IR data of compound 4k (DV-12)

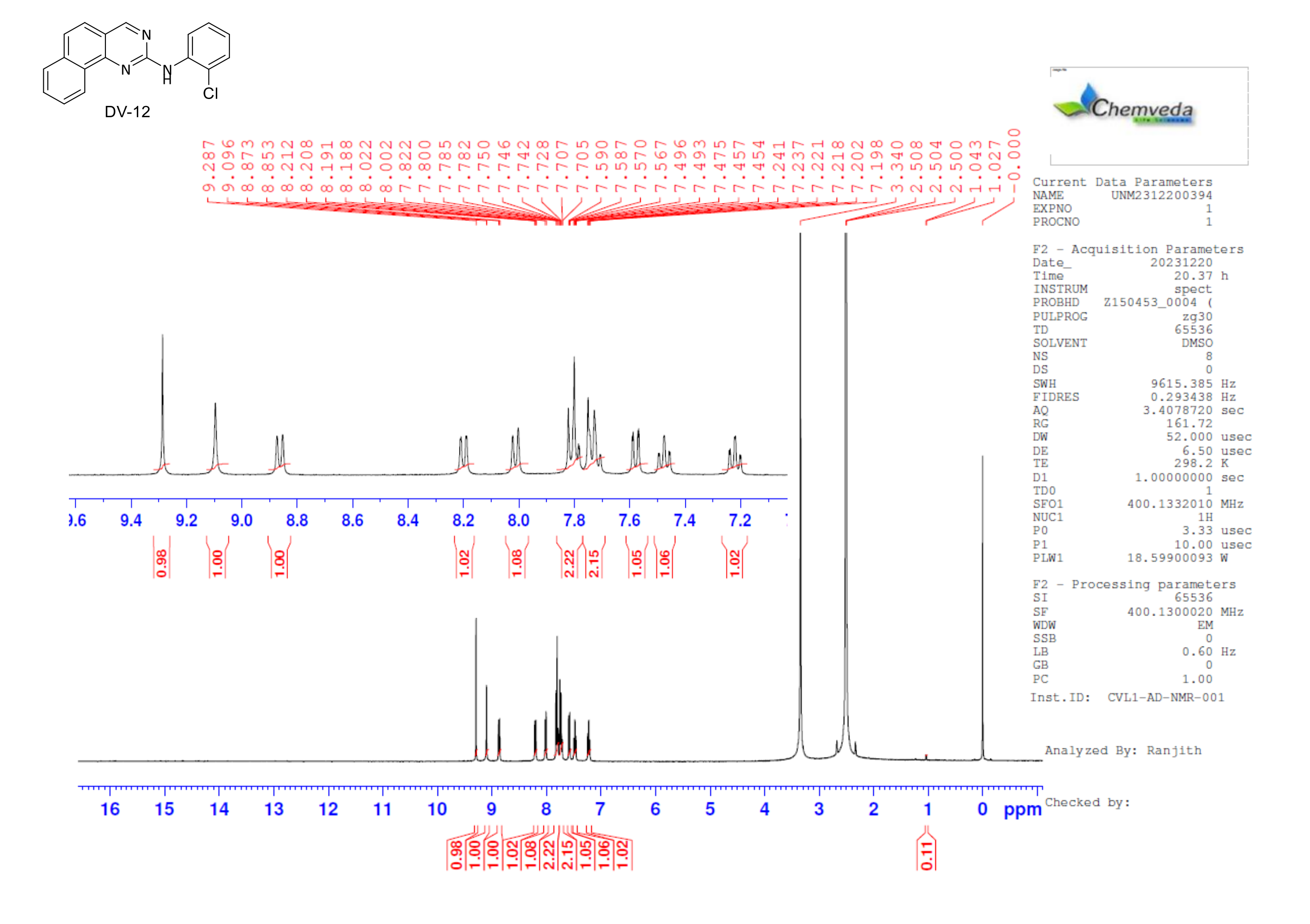

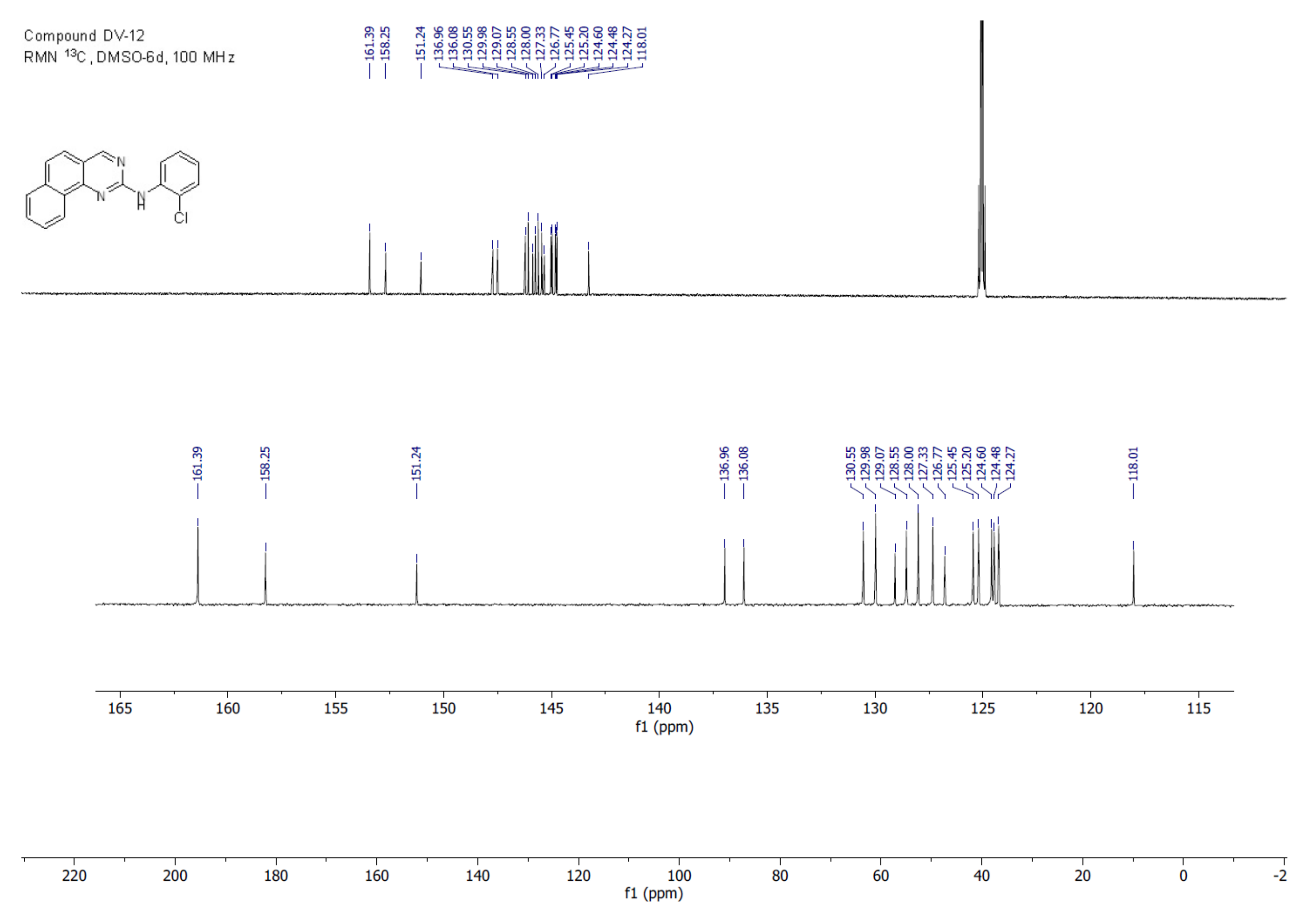

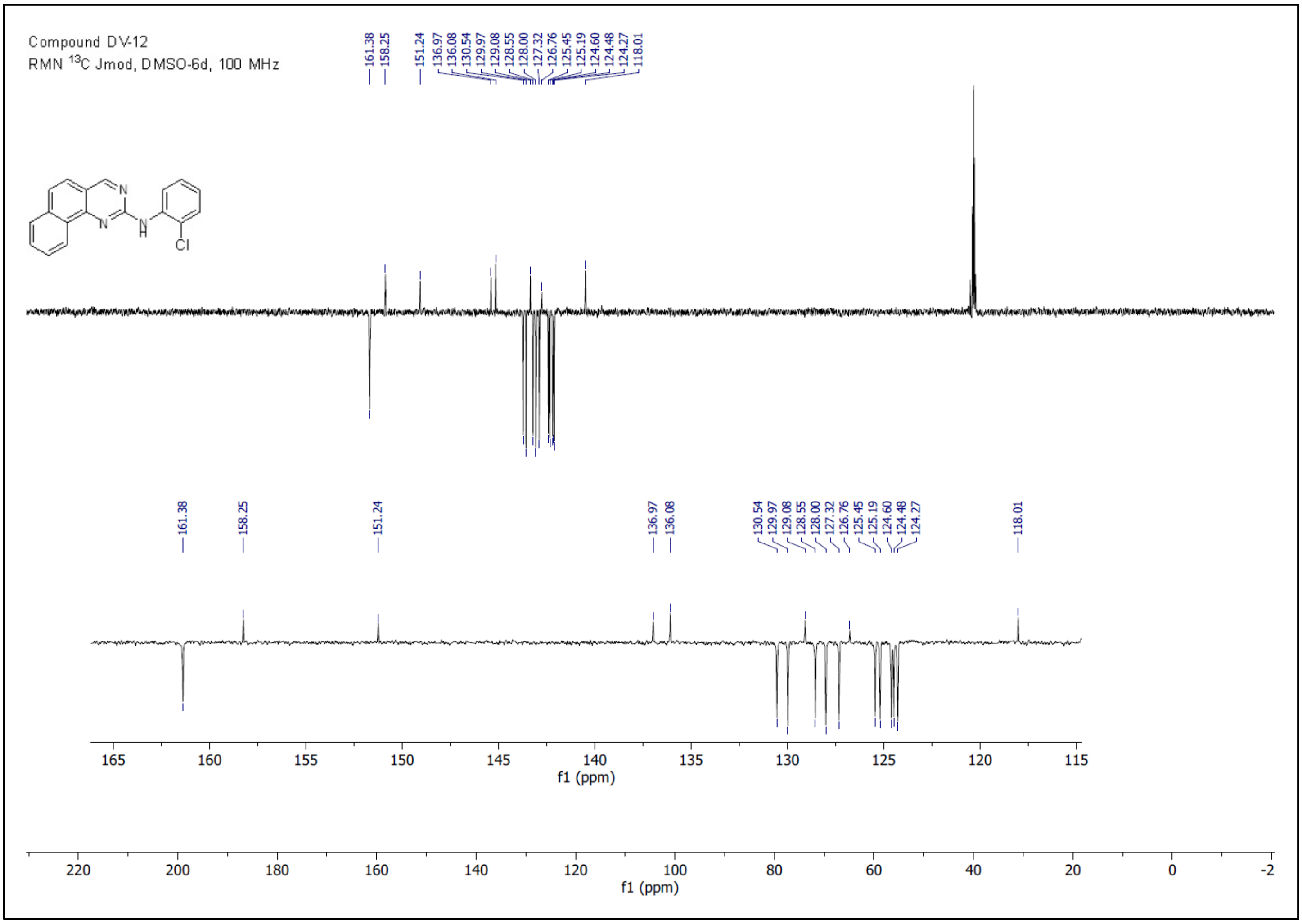
Compound DV-12
RMN 13C Jmod, DMSO-6d, 100 MHz
161.38
158.25
151.24
136.97
136.08
130.54
129.97
129.08
128.55
128.00
127.32
126.76
125.45
125.19
124.60
124.48
124.27
118.01
N
N
N
H
Cl
f1 (ppm)
f1 (ppm)

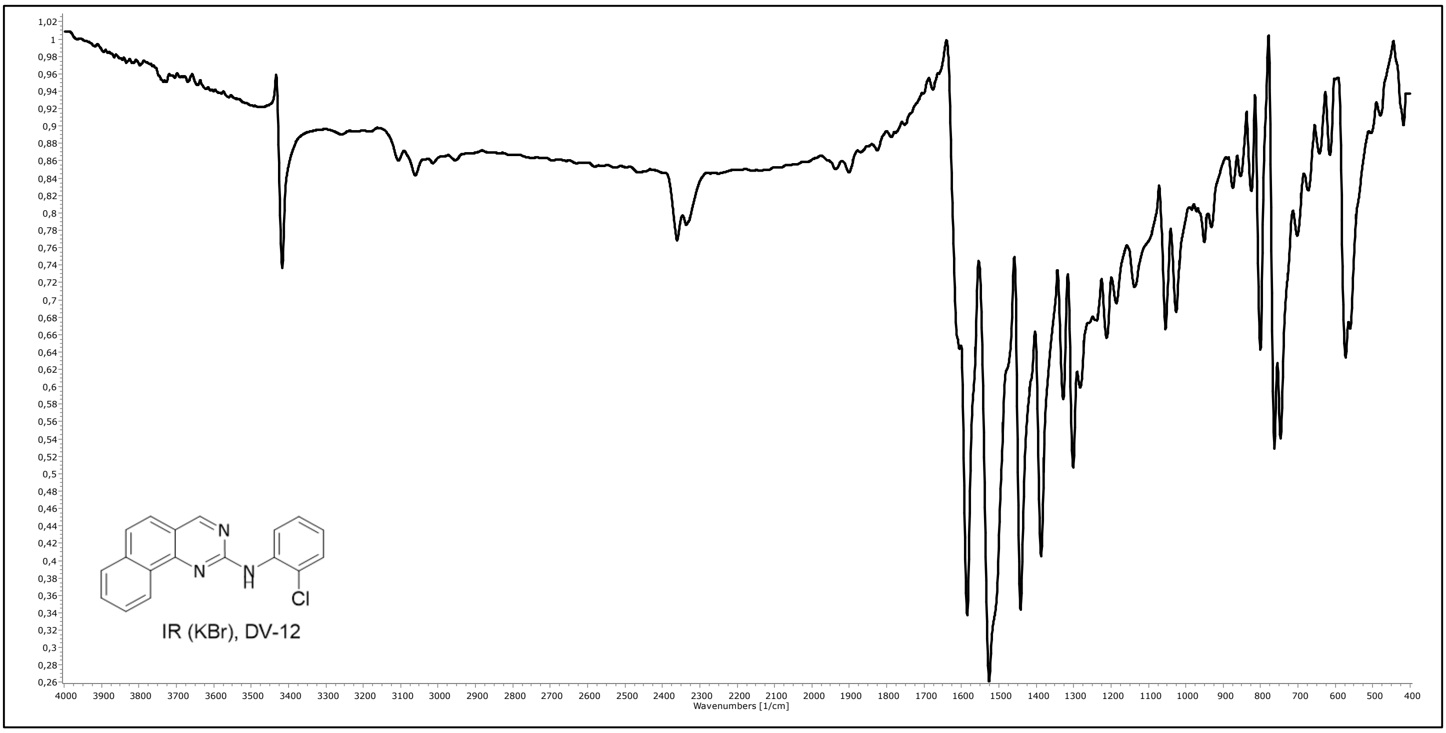
IR (KBr), DV-12
N
N
N
H
Cl
Wavenumbers [1/cm]

Spectra: ^{1}H, ^{13}C, ^{13}C Jmod NMR and FT-IR data of compound 4l (DV-13)

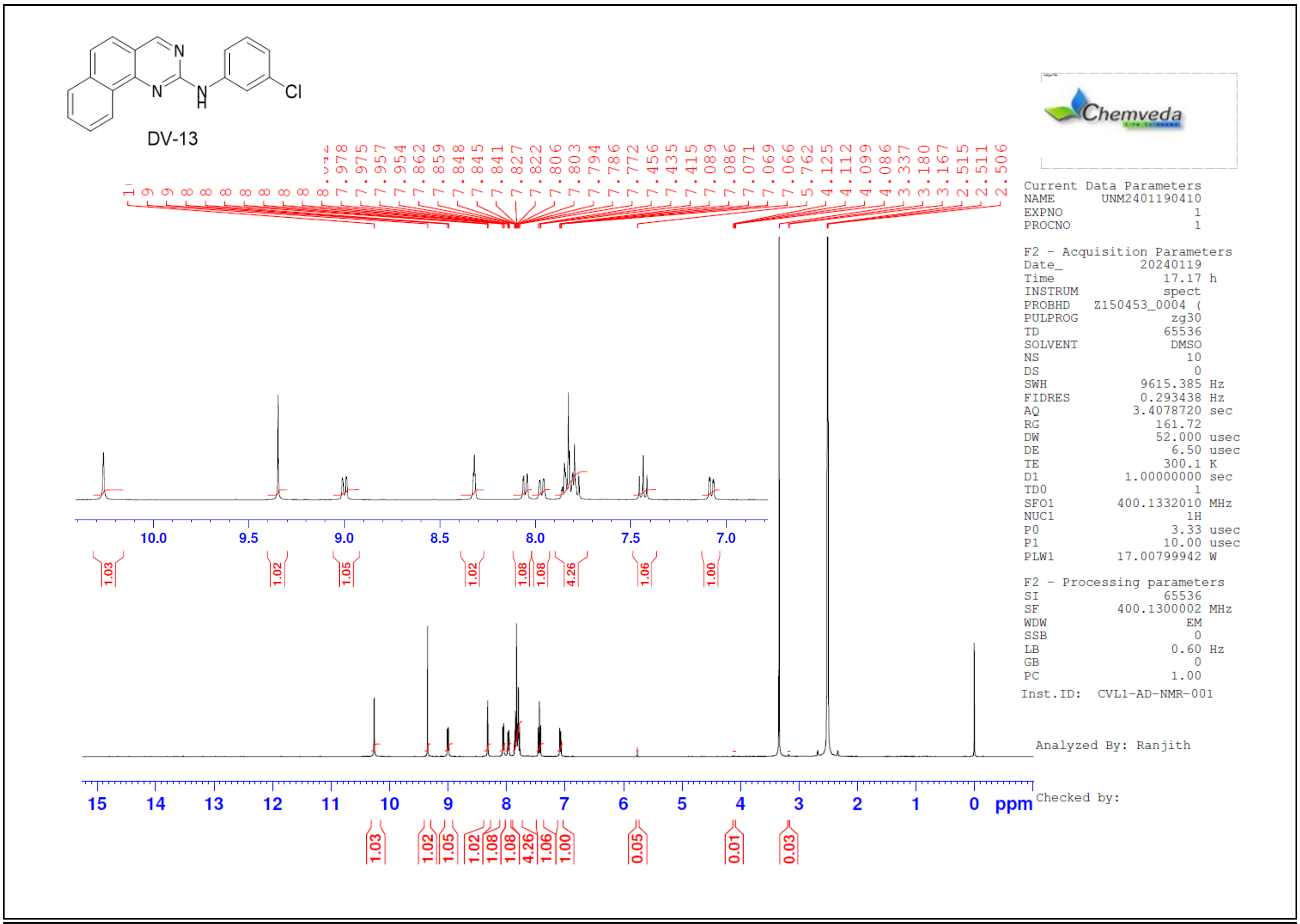

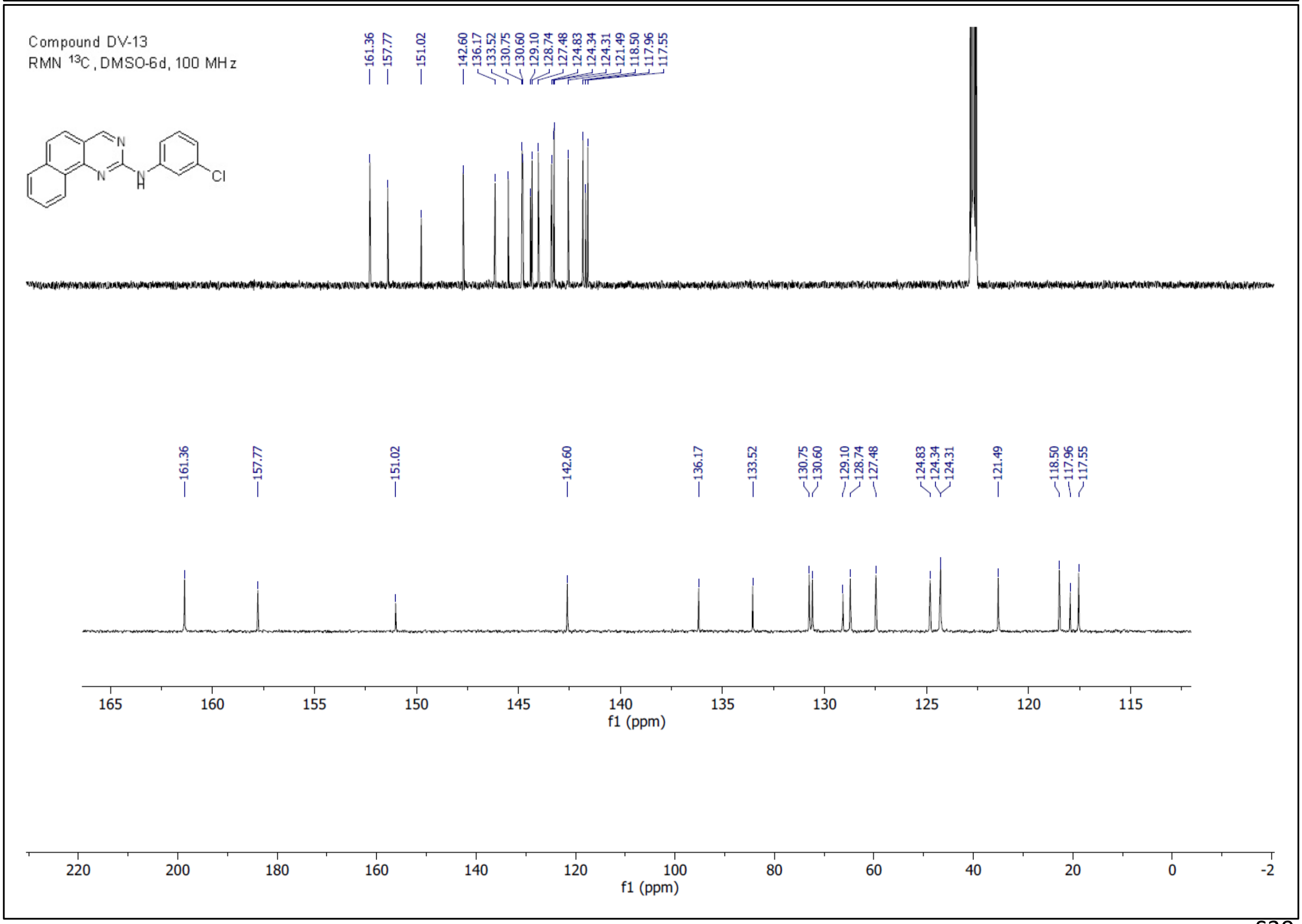

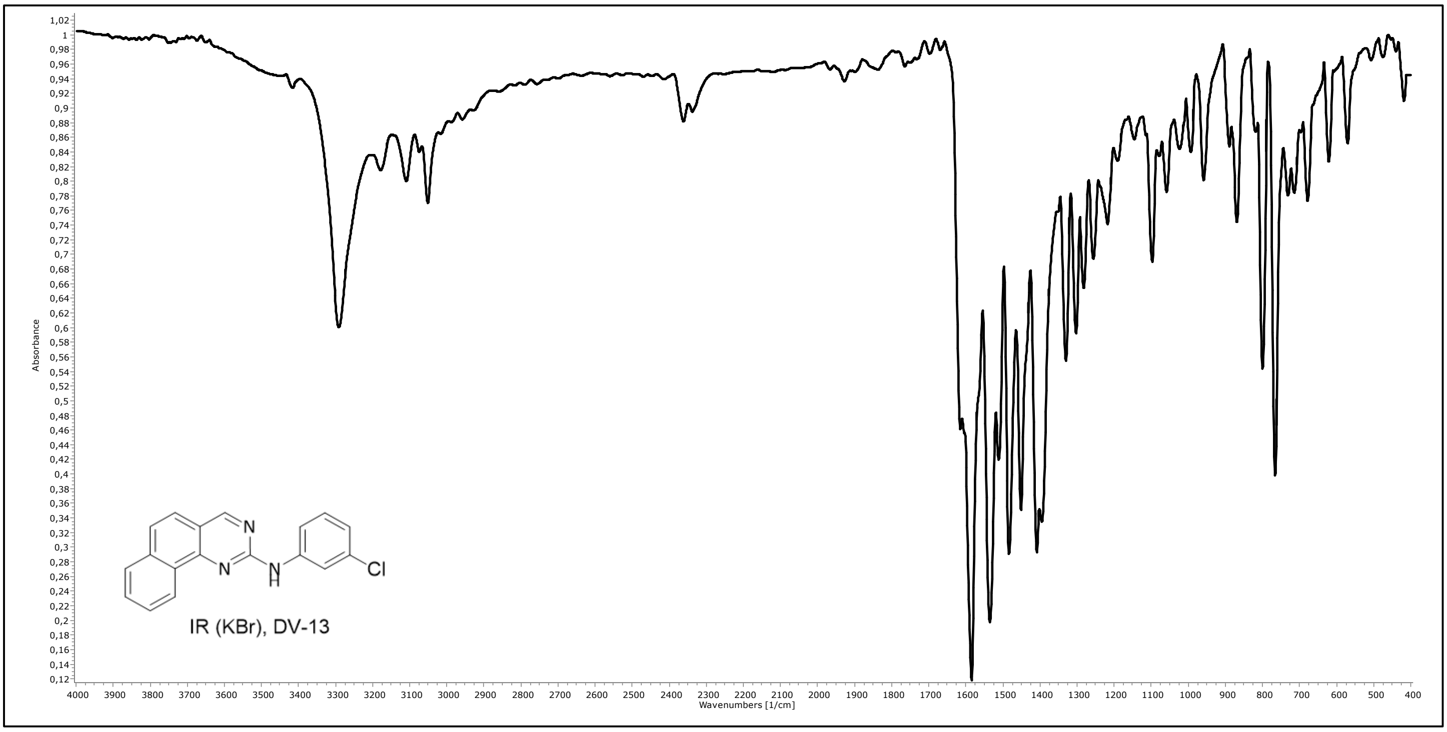
Absorbance
Wavenumbers [1/cm]
N
N
N
H
Cl
IR (KBr), DV-13

Spectra: ^{1}H, ^{13}C, ^{13}C Jmod NMR and FT-IR data of compound 4m (DV-14)

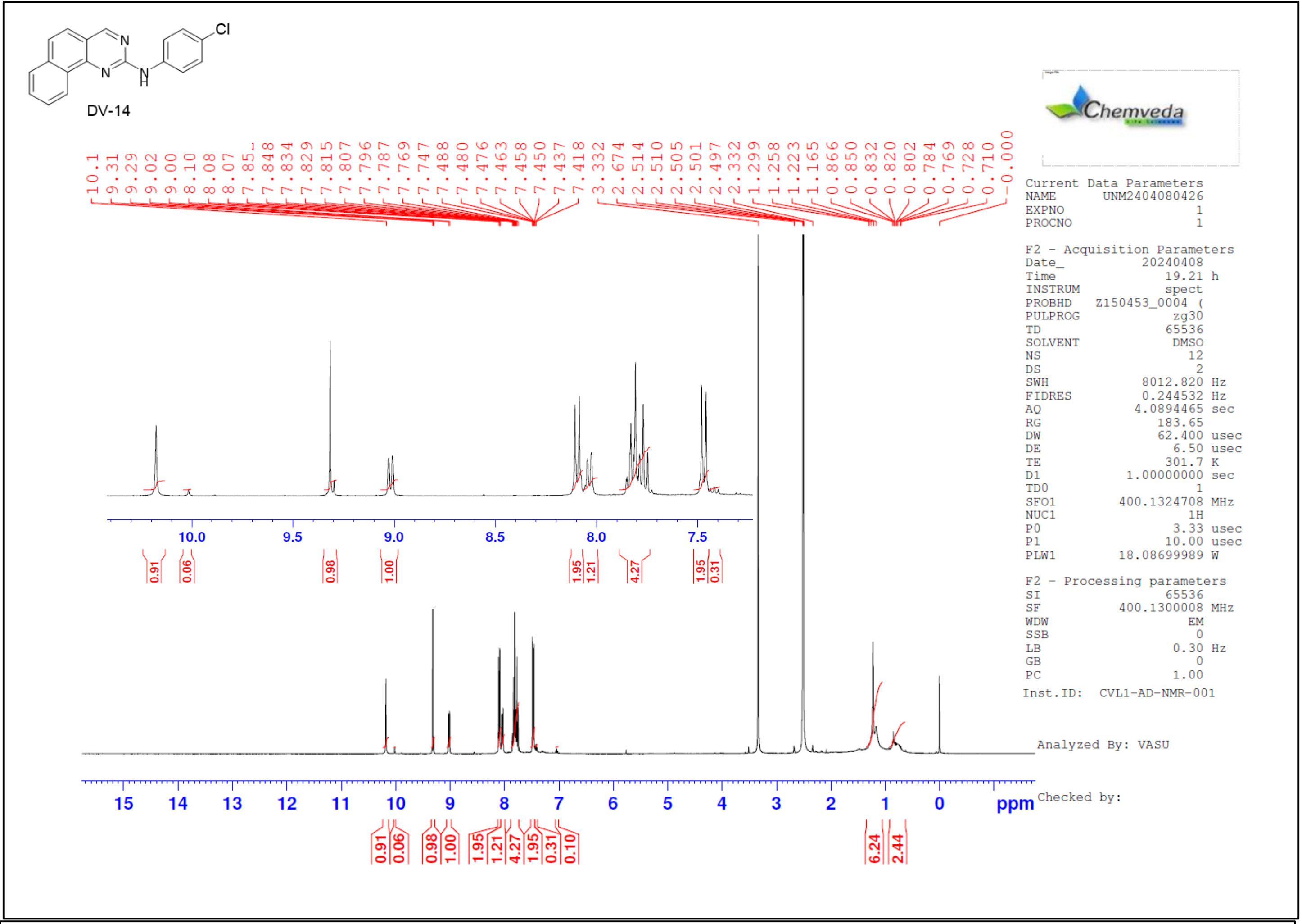

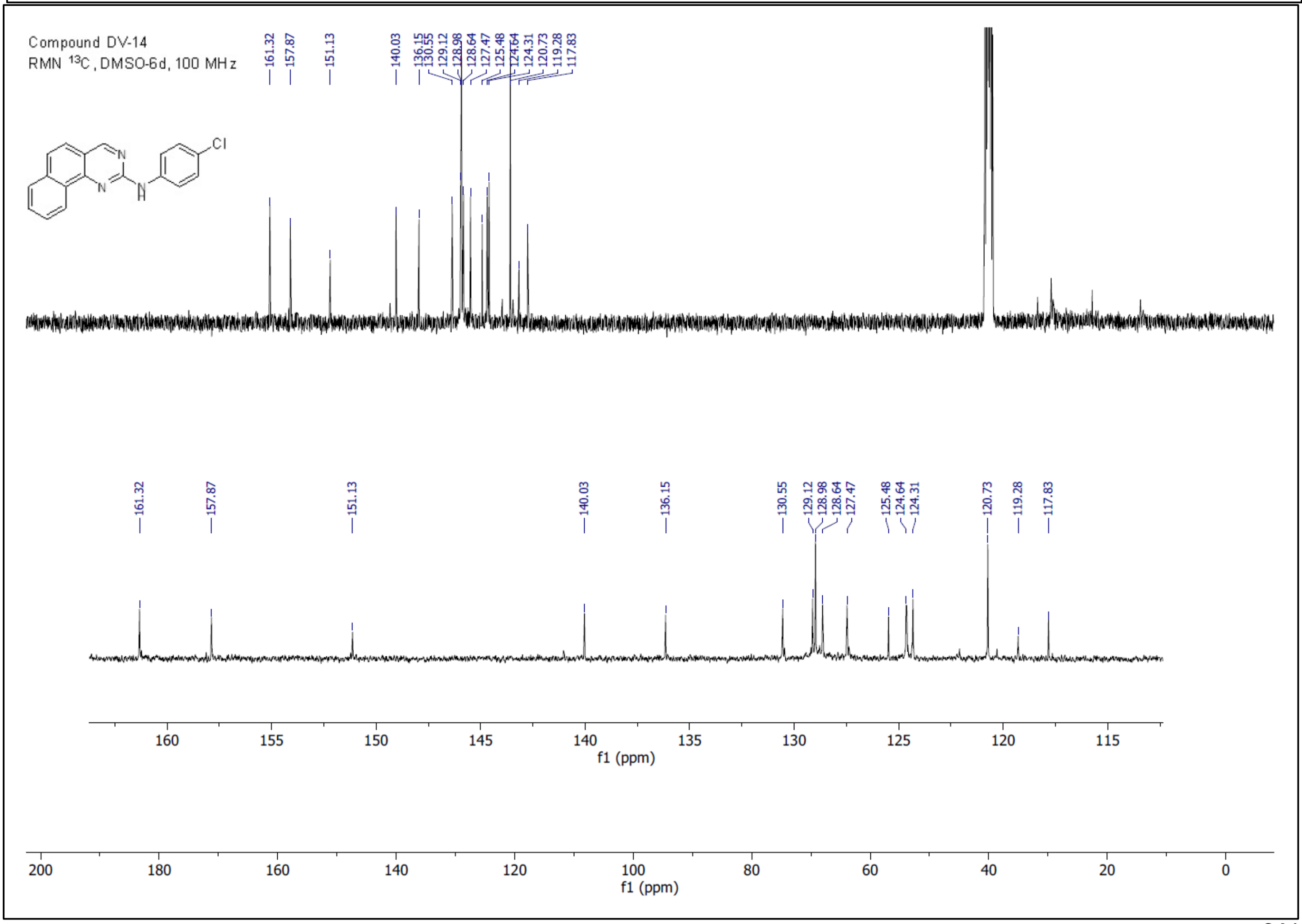

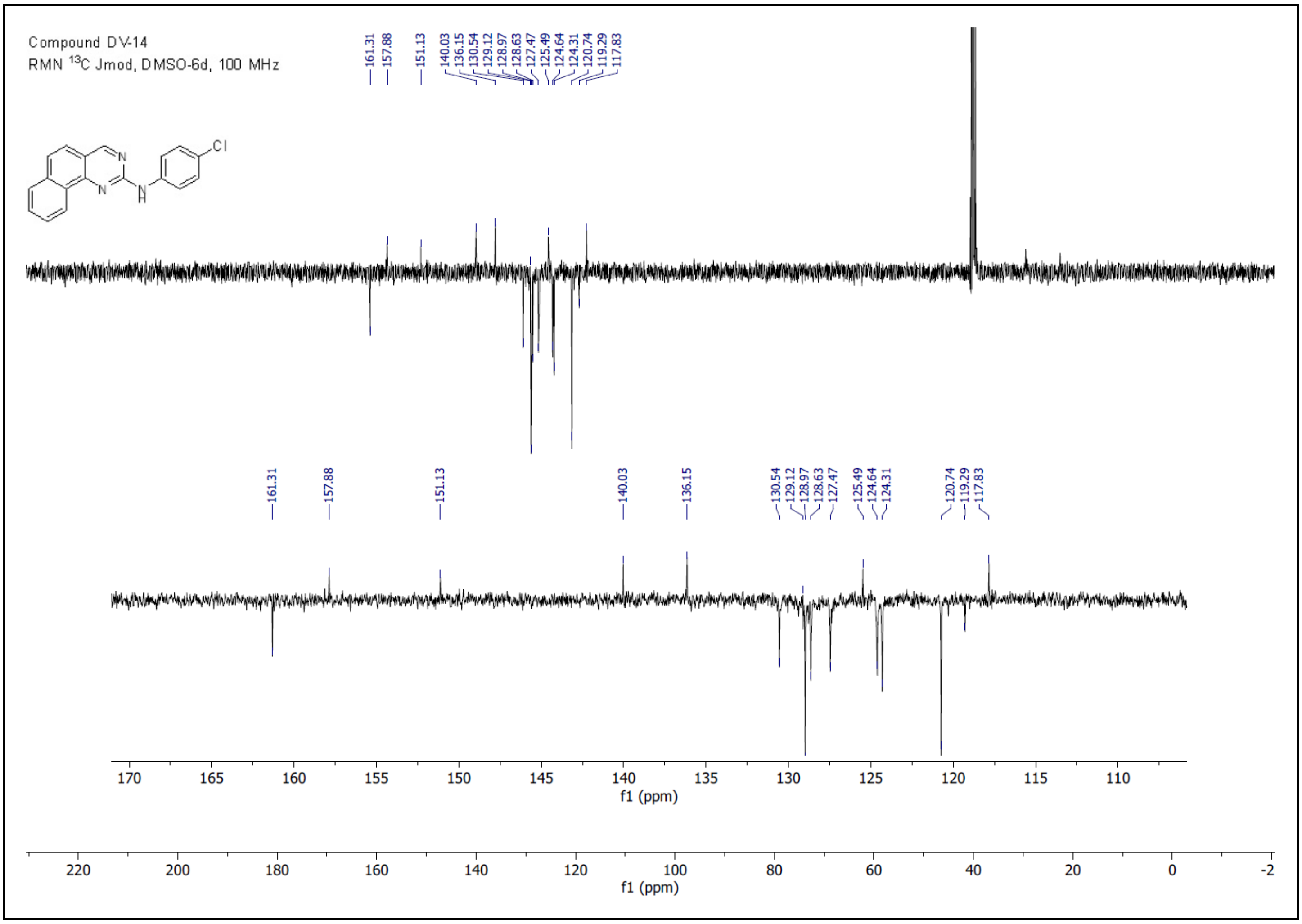
Compound DV-14
RMN ¹³C Jmod, DMSO-6d, 100 MHz
Cl
N
N
H
161.31
157.88
151.13
140.03
136.15
130.54
129.12
128.97
128.63
127.47
125.49
124.64
124.31
120.74
119.29
117.83
f1 (ppm)

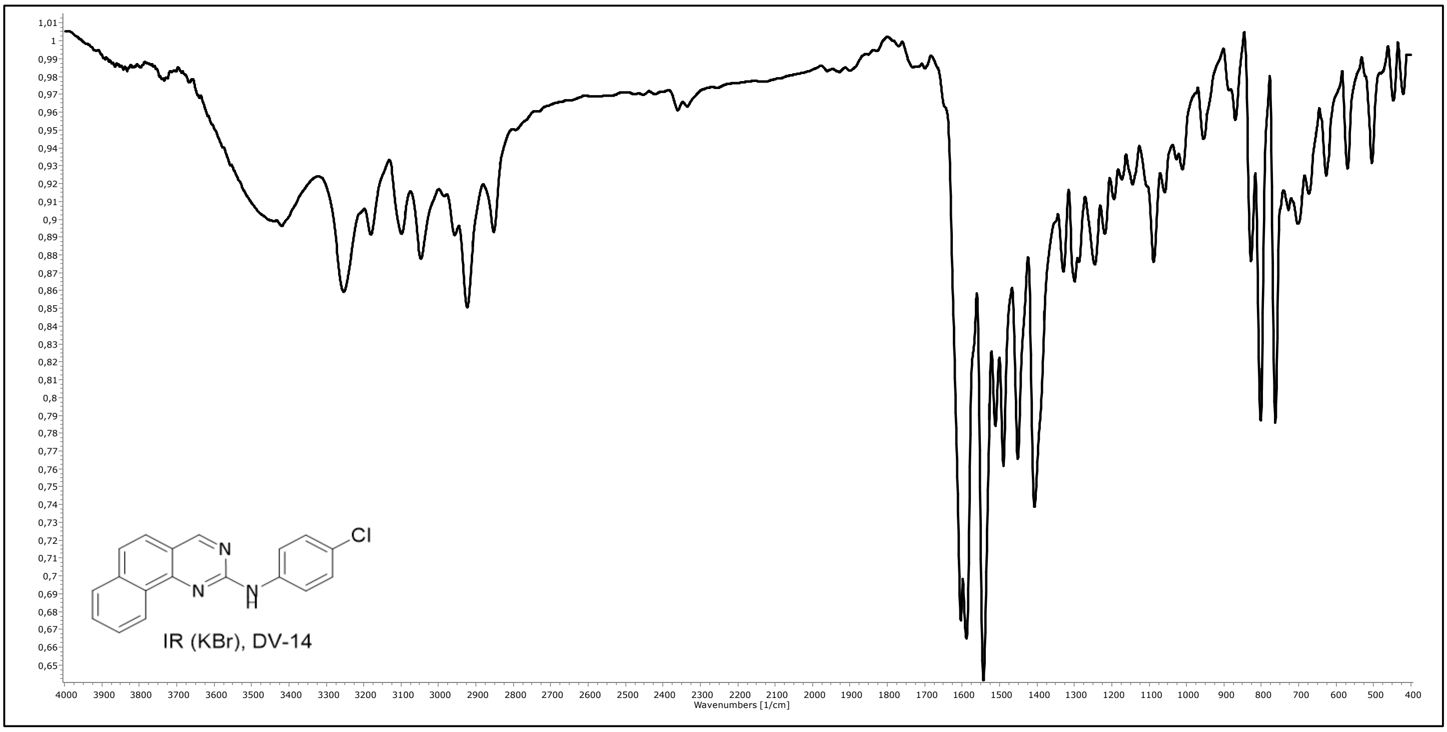
Cl
N
N
H
IR (KBr), DV-14
Wavenumbers [1/cm]

Spectra: ^{1}H, ^{13}C, ^{13}C Jmod NMR and FT-IR data of compound 4n (DV-15)

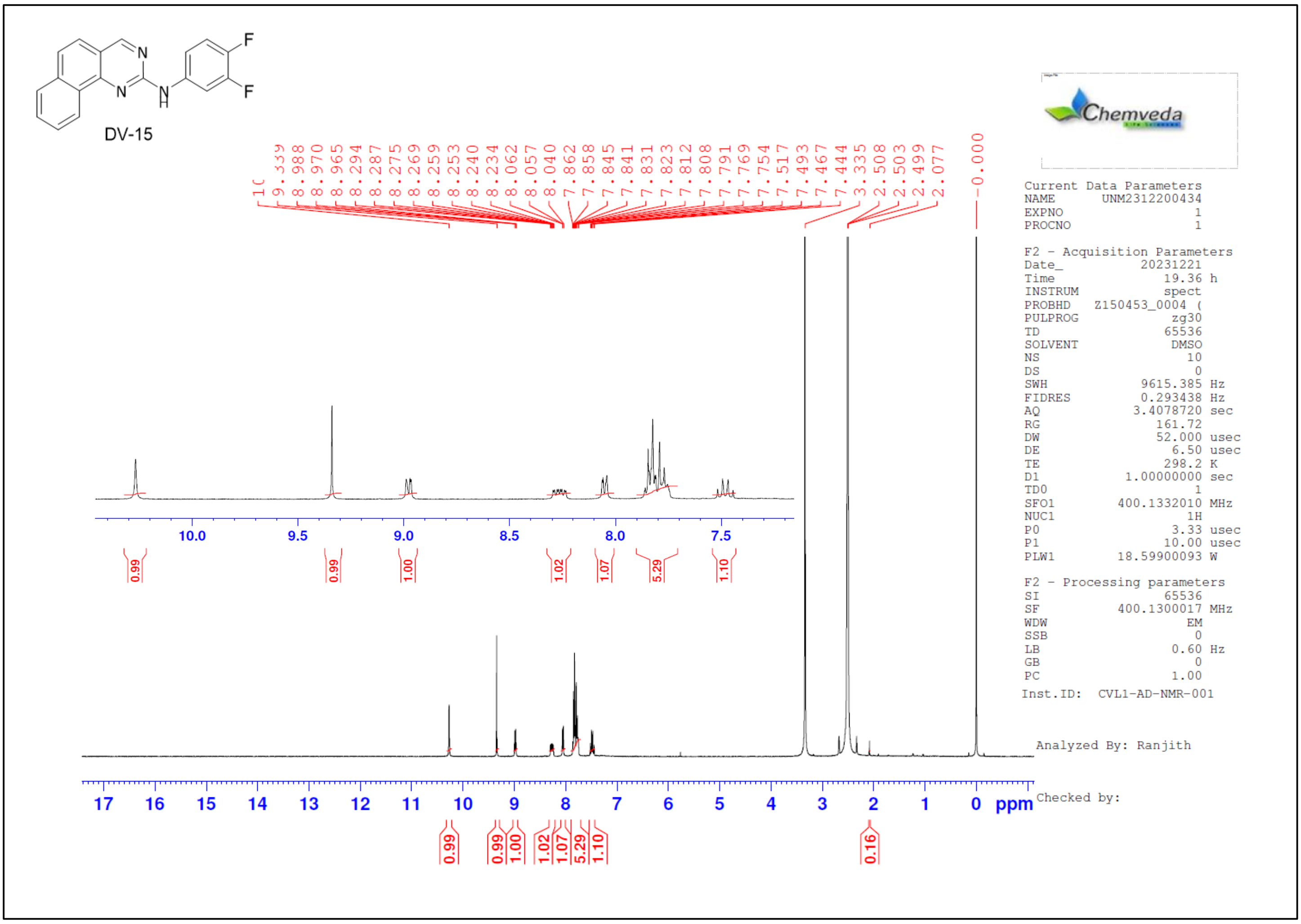

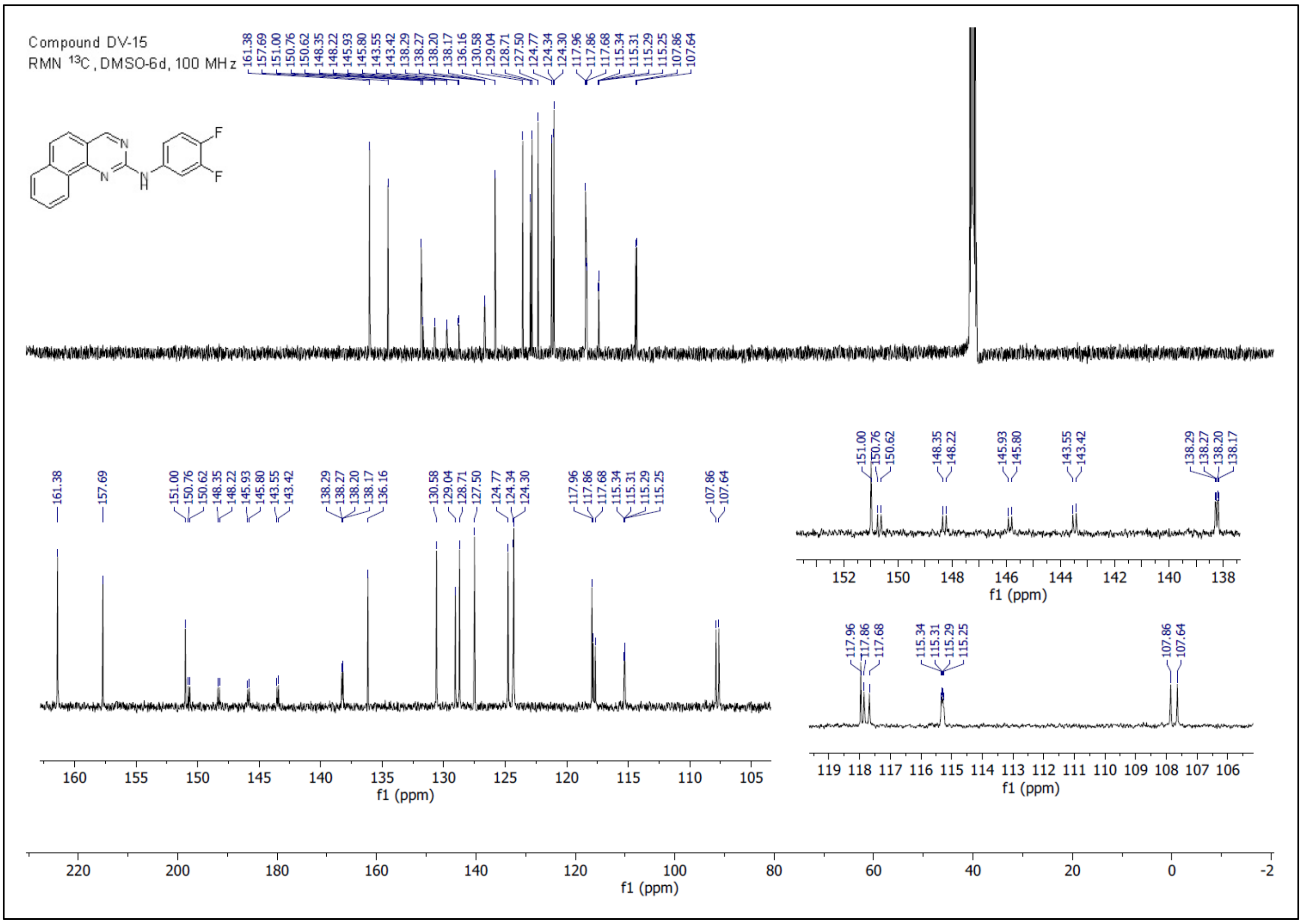

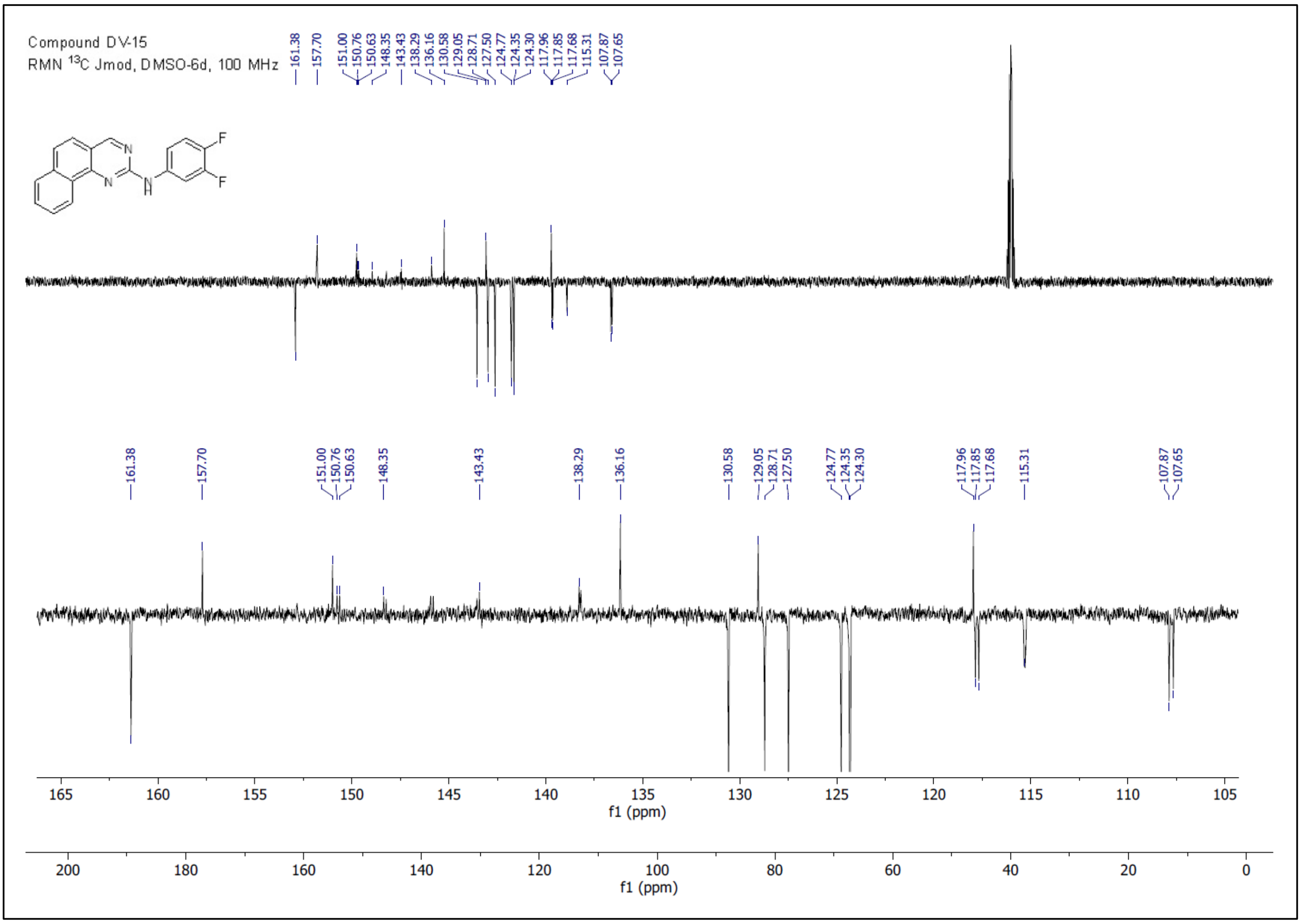
Compound DV-15
RMN ^{13}C Jmod, DMSO-6d, 100 MHz
161.38
157.70
151.00
150.76
150.63
148.35
143.43
138.29
136.16
130.58
129.05
128.71
127.50
124.77
124.35
124.30
117.96
117.85
117.68
115.31
107.87
107.65
F
F
N
N
N
H
f1 (ppm)
f1 (ppm)

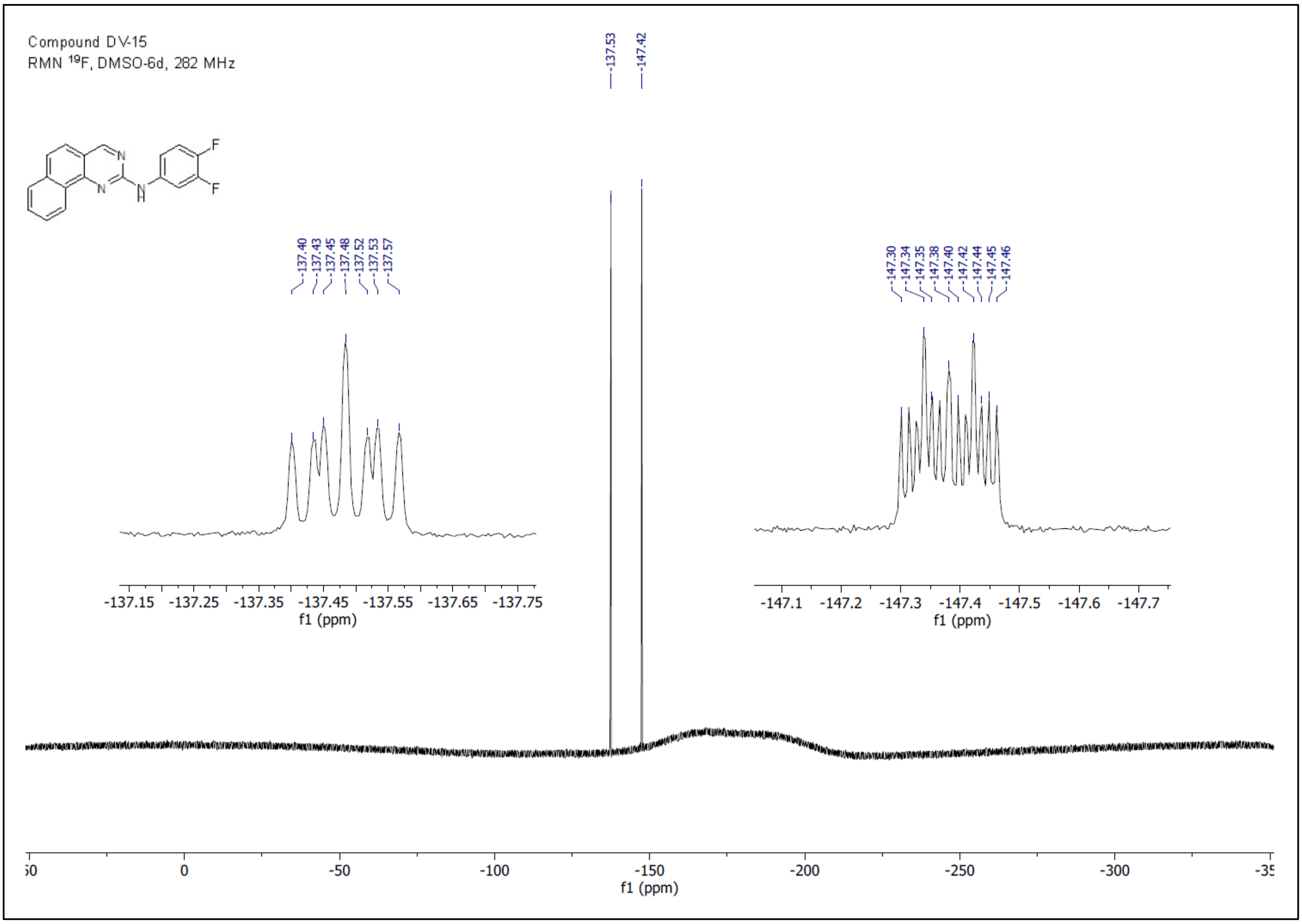
Compound DV-15
RMN ^{19}F, DMSO-6d, 282 MHz
-137.53
-147.42
-137.40
-137.43
-137.45
-137.48
-137.52
-137.53
-137.57
-147.30
-147.34
-147.35
-147.38
-147.40
-147.42
-147.44
-147.45
-147.46
F
F
N
N
N
H
f1 (ppm)
f1 (ppm)
f1 (ppm)

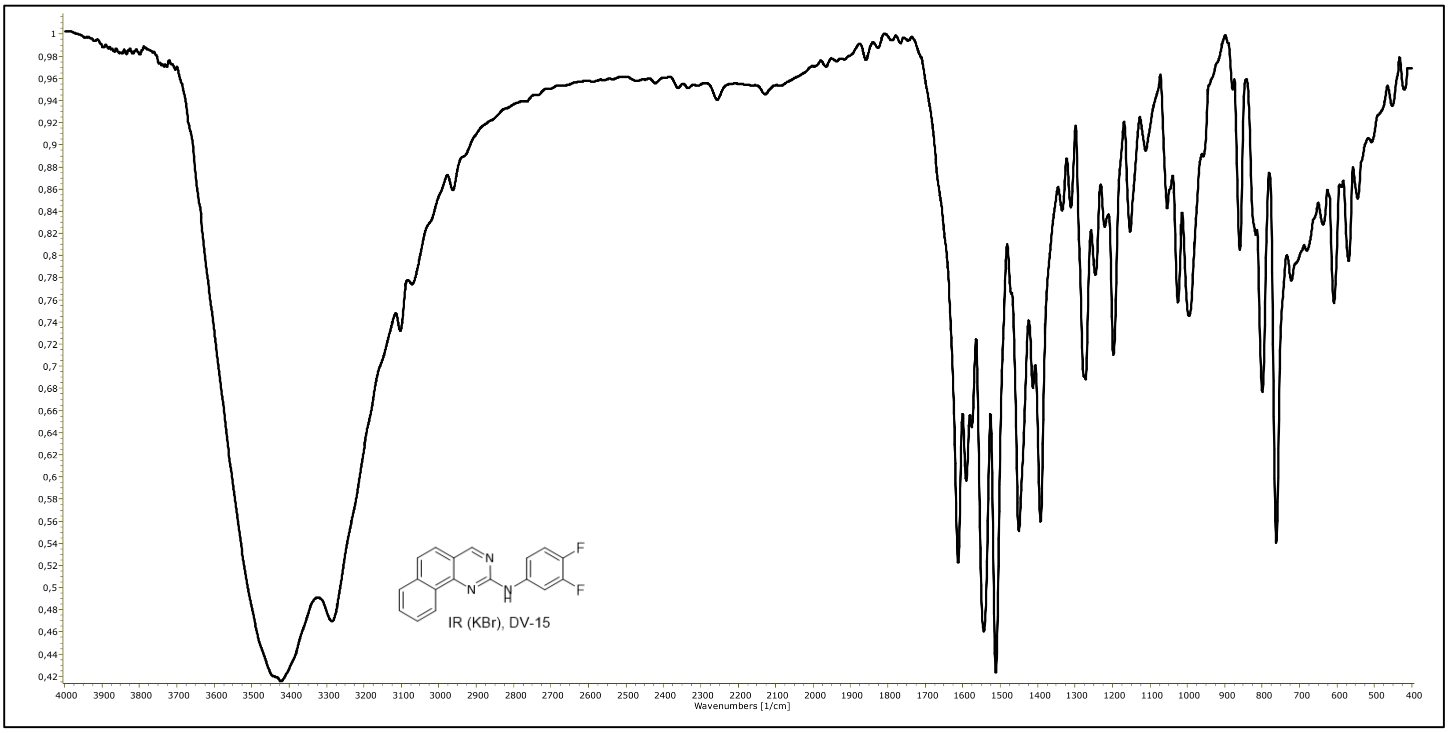
IR (KBr), DV-15
Wavenumbers [1/cm]
N
N
N
H
F
F

Spectra: ^{1}H, ^{13}C, ^{13}C Jmod NMR and FT-IR data of compound 4o (DV-16)

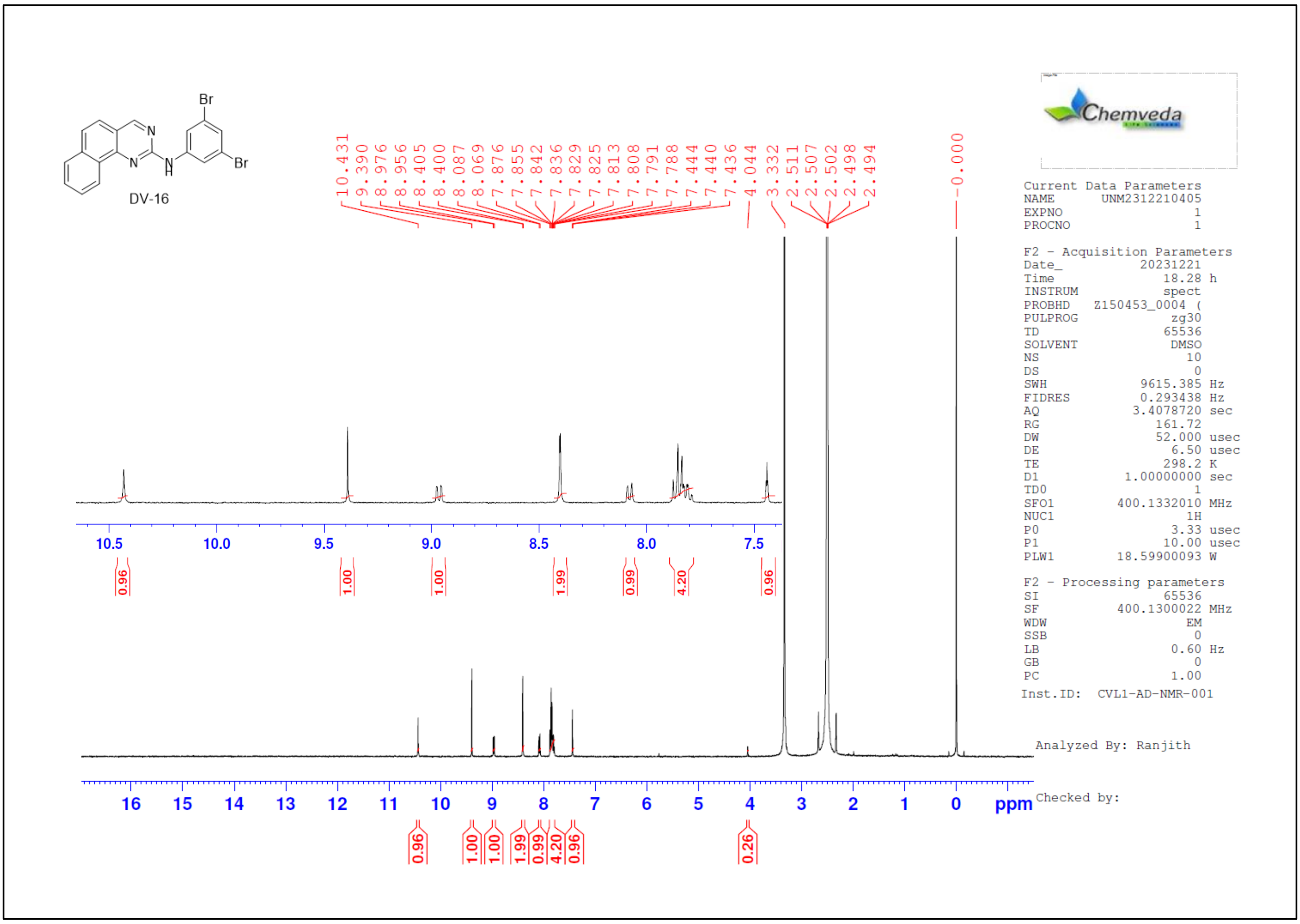

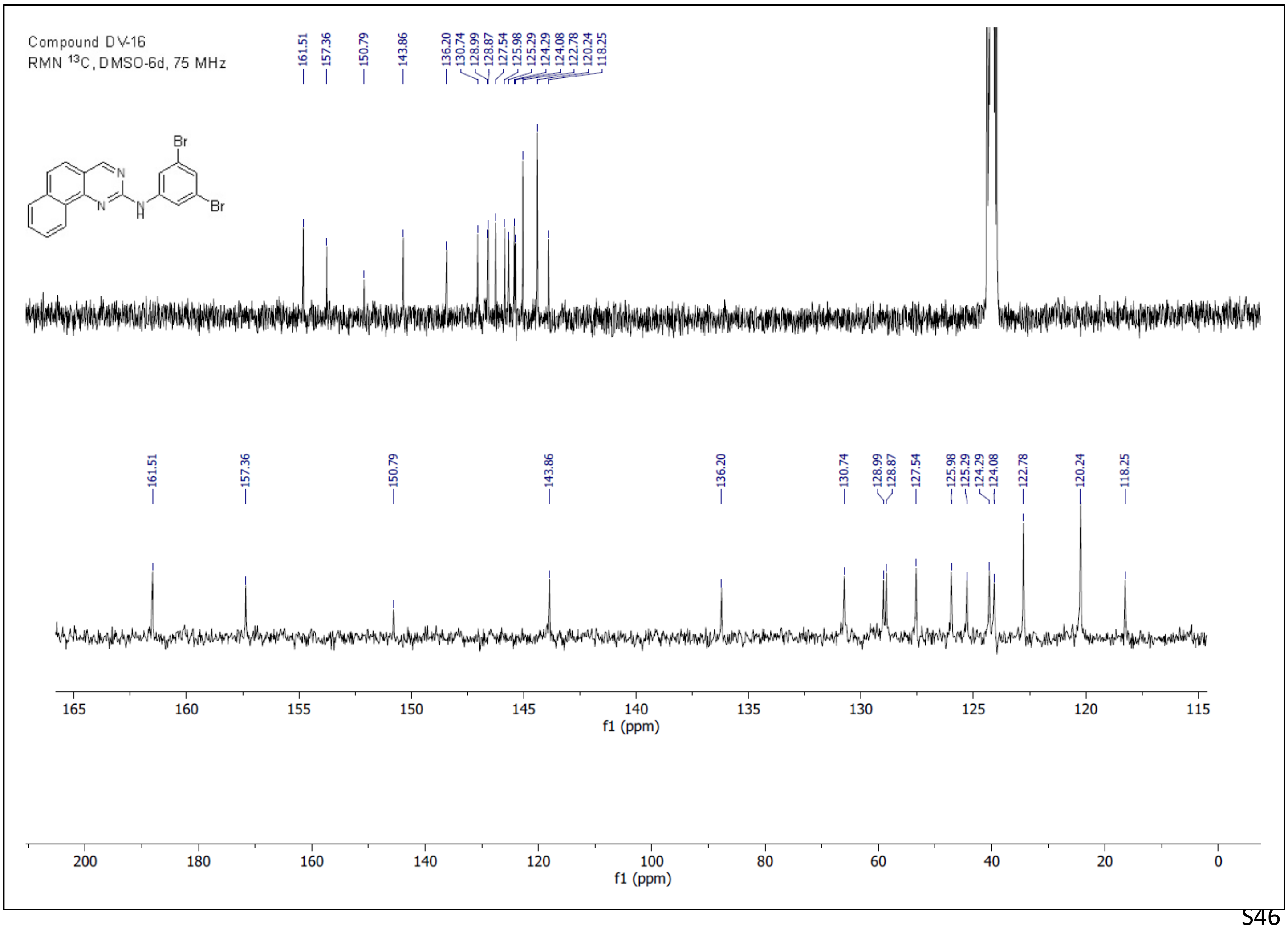

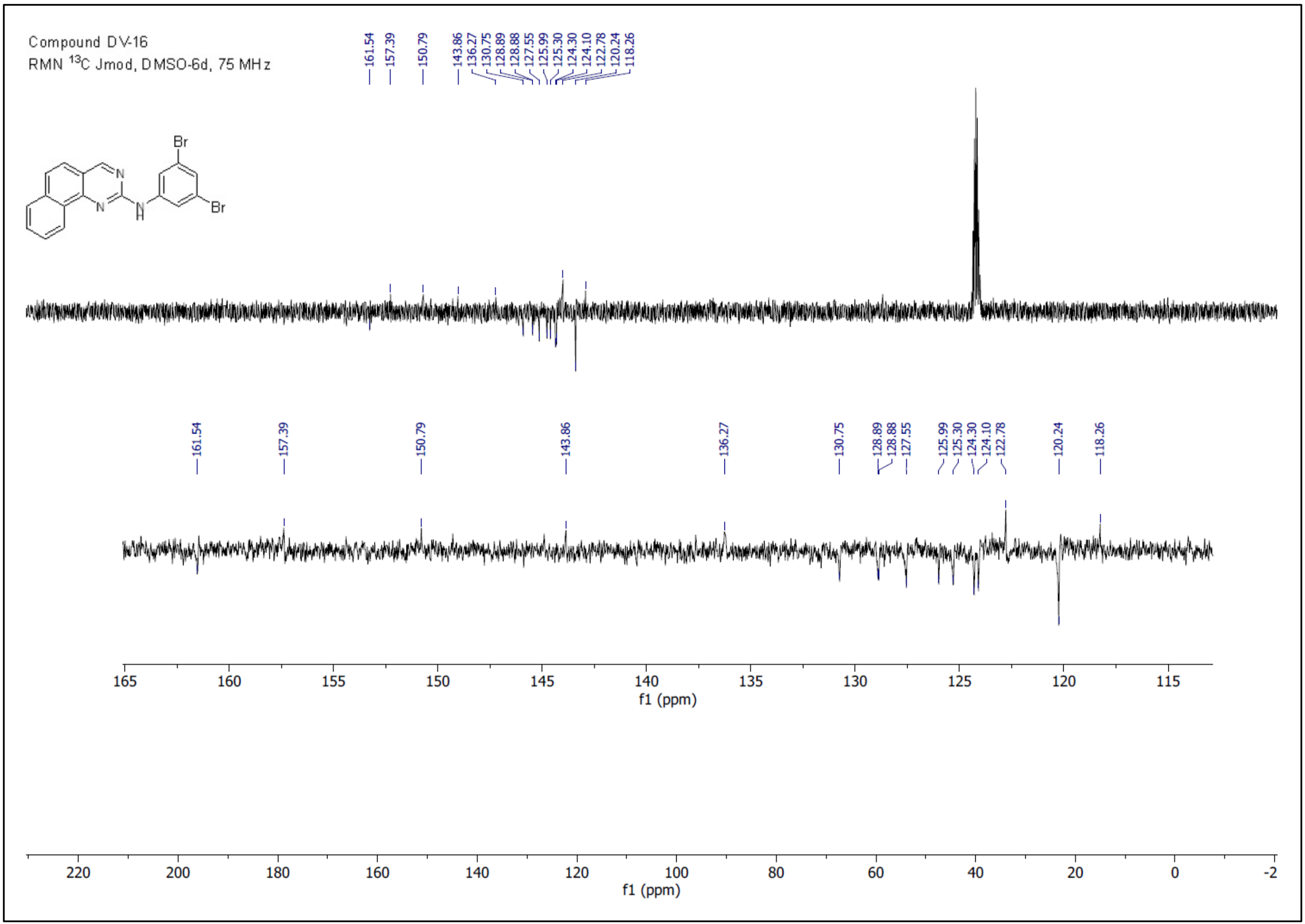

Compound DV-16
RMN 13C Jmod, DMSO-6d, 75 MHz
161.54
157.39
150.79
143.86
136.27
130.75
128.89
128.88
127.55
125.99
125.30
124.30
124.10
122.78
120.24
118.26
Br
N
N
N
H
Br
f1 (ppm)

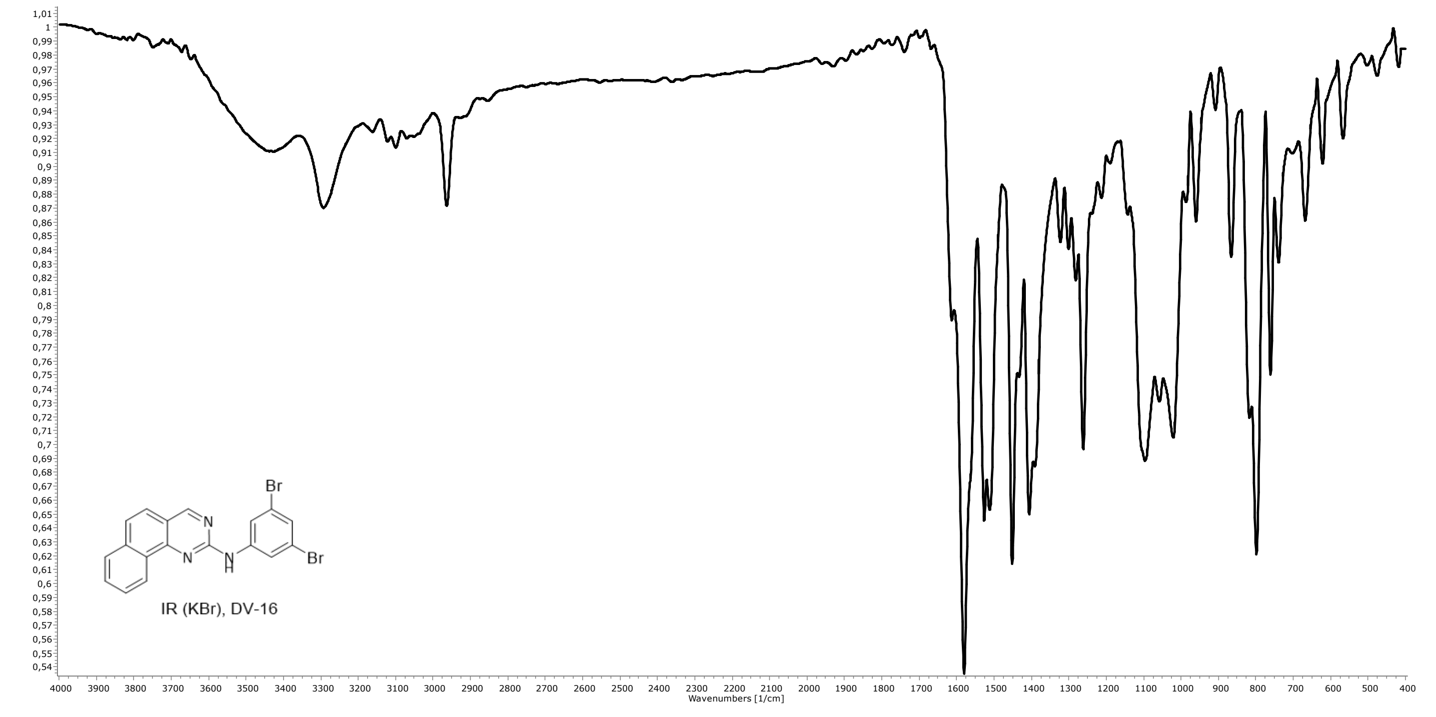

Wavenumbers [1/cm]
Br
N
N
N
H
Br
IR (KBr), DV-16

Spectra: ^{1}H, ^{13}C, ^{13}C Jmod NMR and FT-IR data of compound 4p (DV-17)

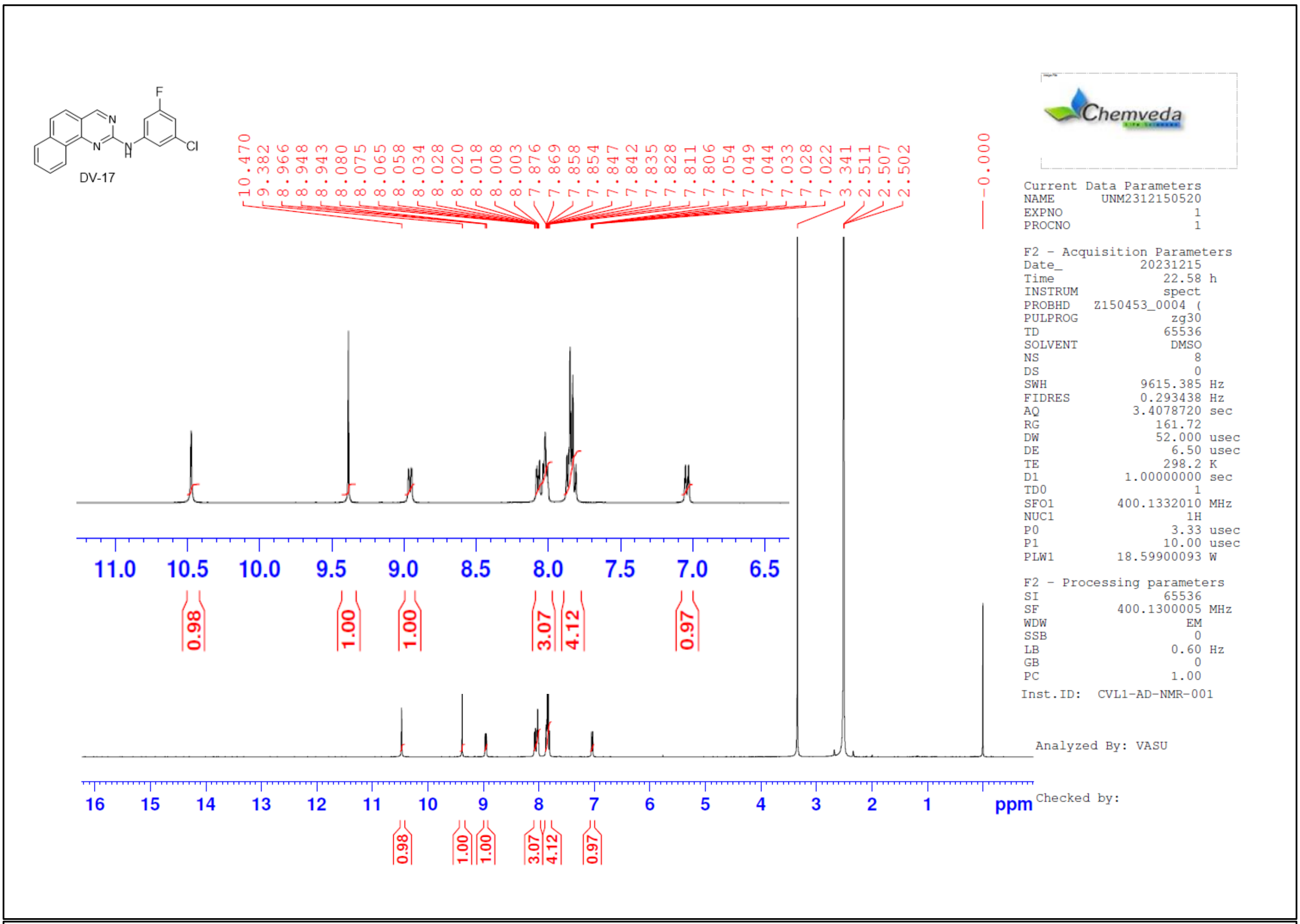

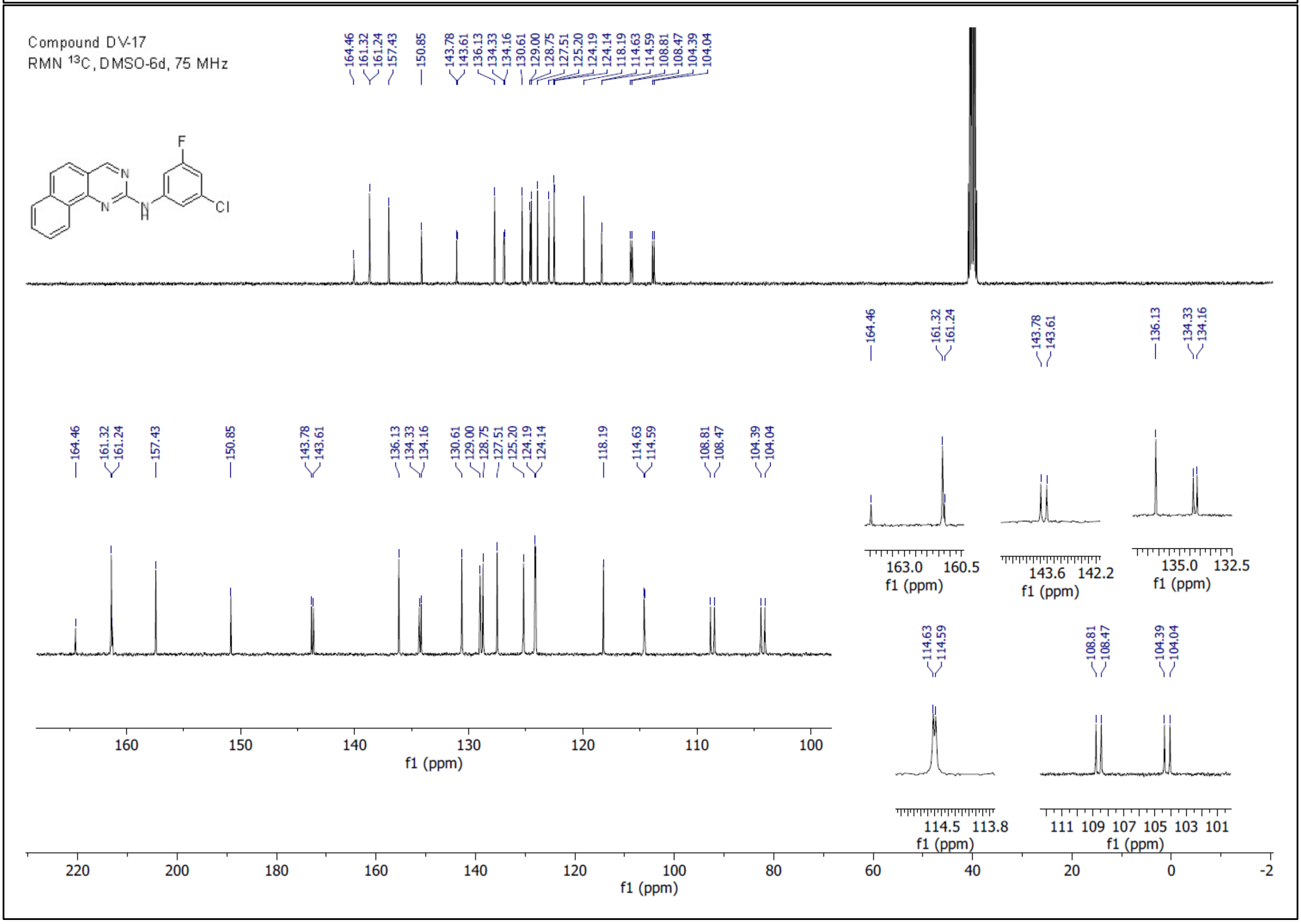

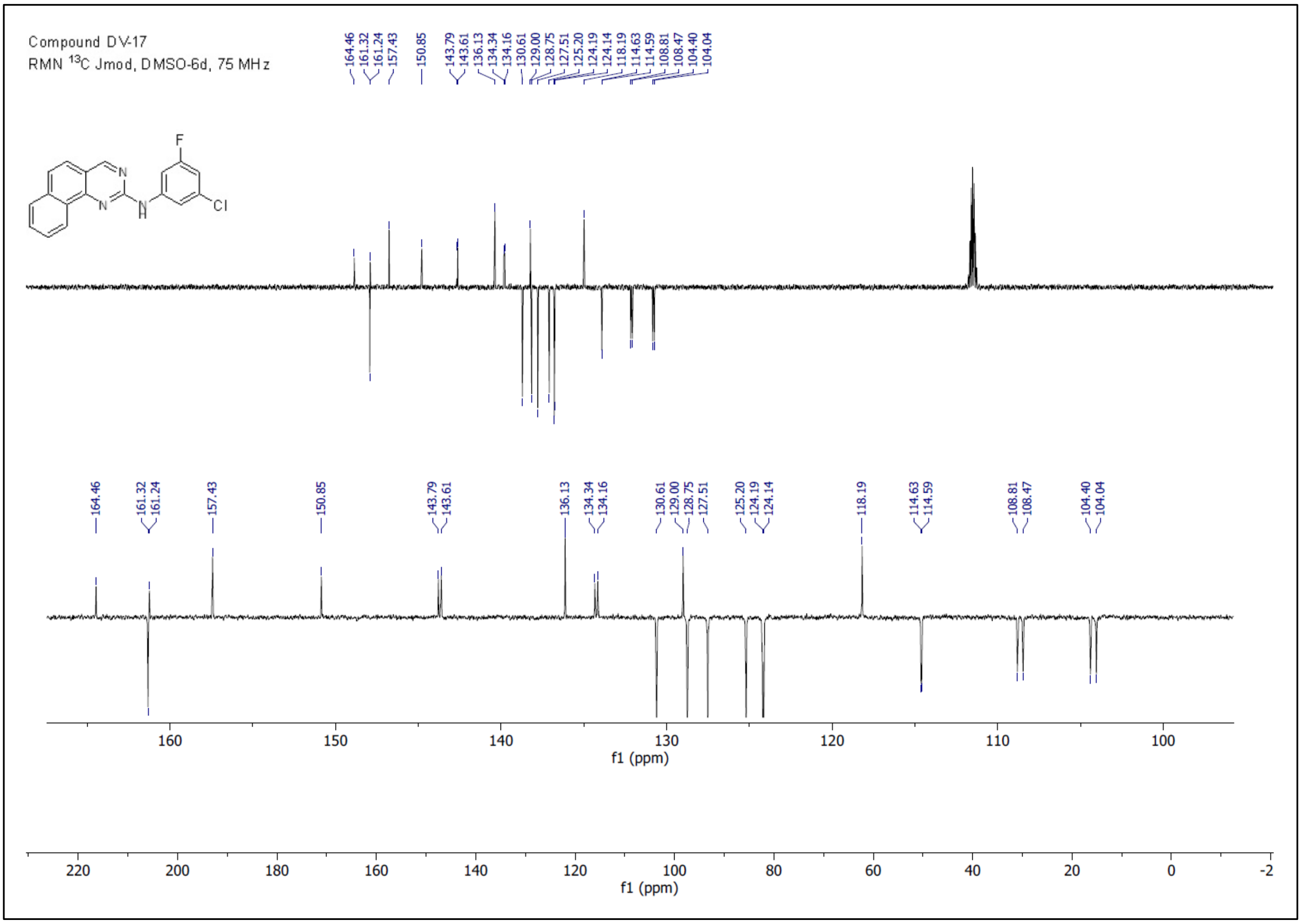
Compound DV-17
RMN 13C Jmod, DMSO-6d, 75 MHz
164.46
161.32
161.24
157.43
150.85
143.79
143.61
136.13
134.34
134.16
130.61
129.00
128.75
127.51
125.20
124.19
124.14
118.19
114.63
114.59
108.81
108.47
104.40
104.04
F
N
N
H
Cl
f1 (ppm)

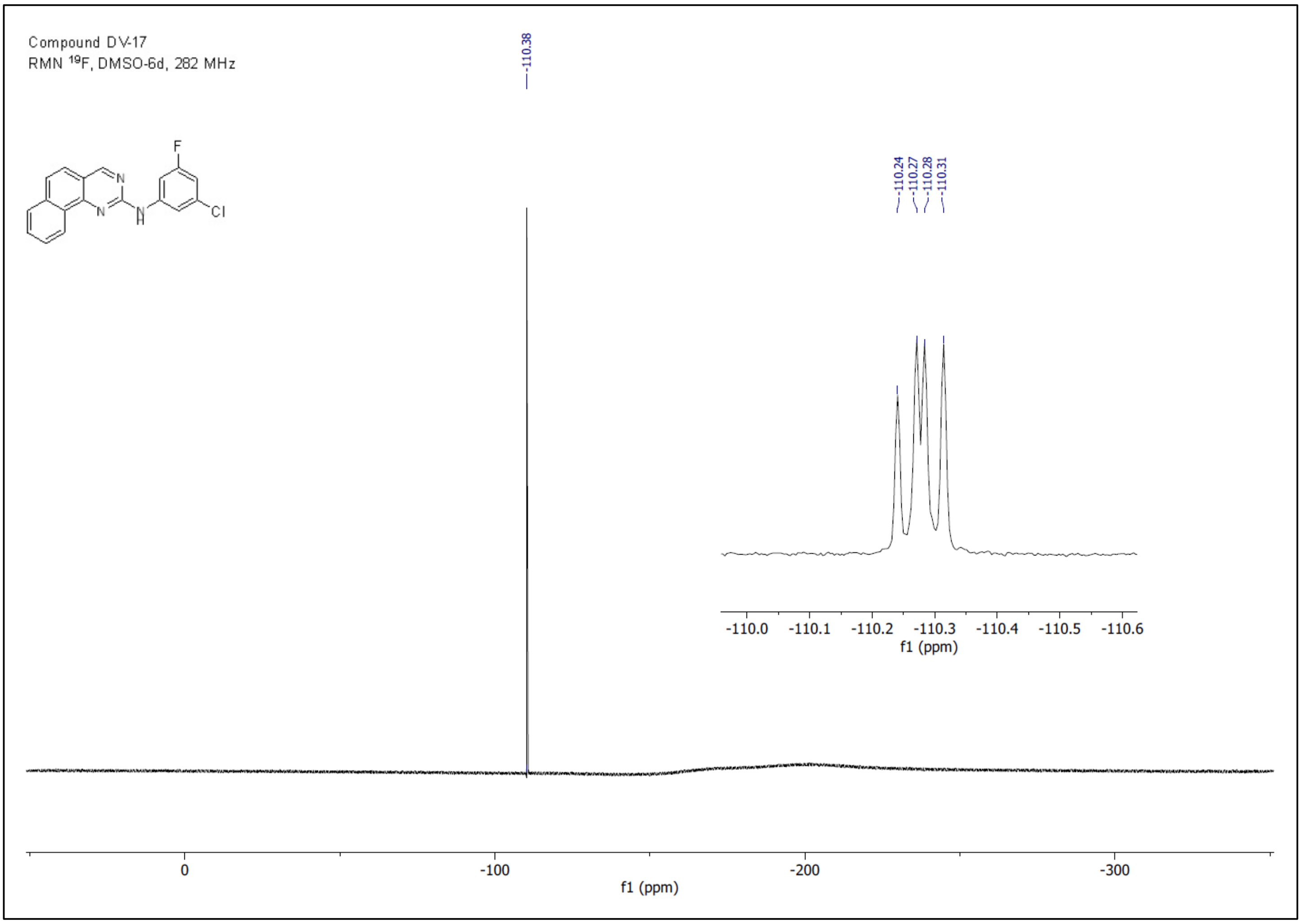
Compound DV-17
RMN 19F, DMSO-6d, 282 MHz
-110.38
-110.24
-110.27
-110.28
-110.31
F
N
N
H
Cl
f1 (ppm)

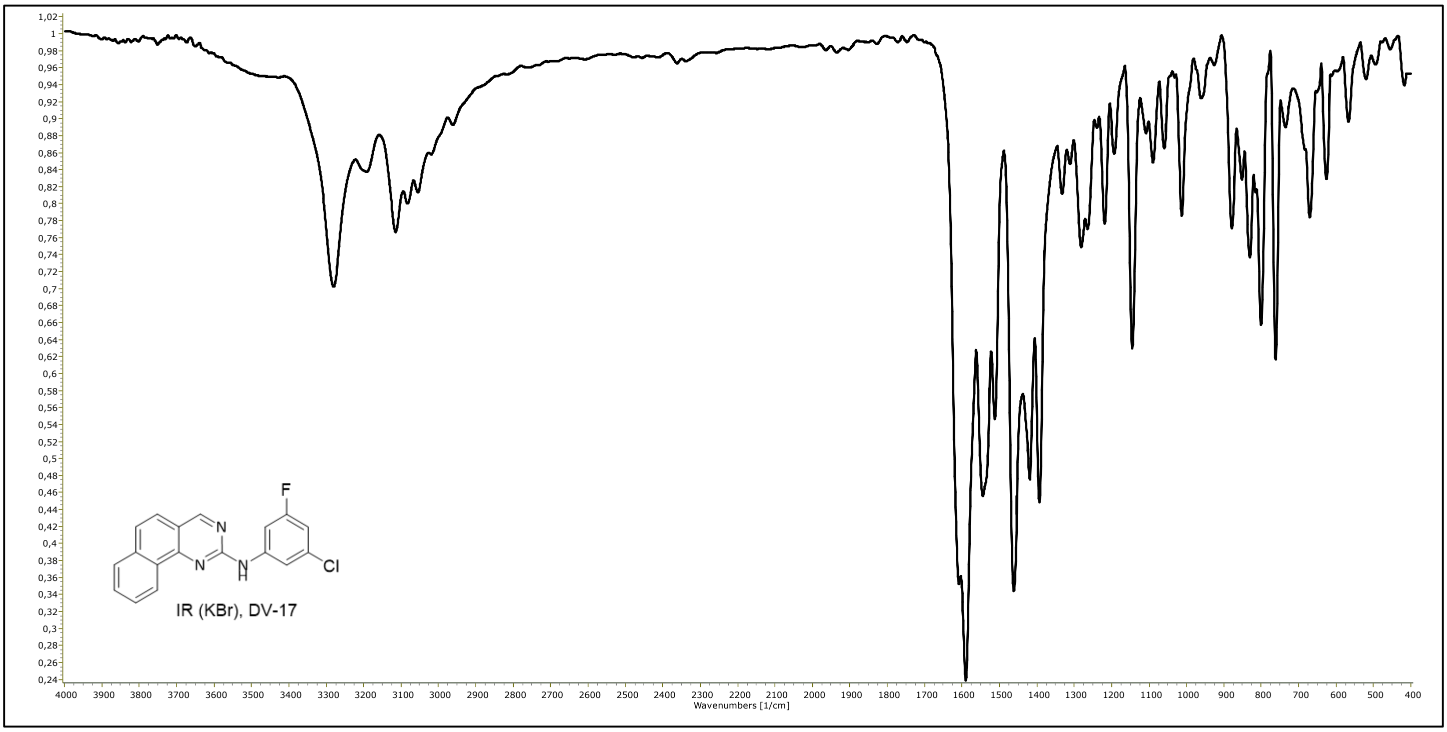

F
N
N
N
H
Cl
IR (KBr), DV-17
Wavenumbers [1/cm]

Spectra: ^{1}H, ^{13}C, ^{13}C Jmod NMR and FT-IR data of compound 4q (DV-18)

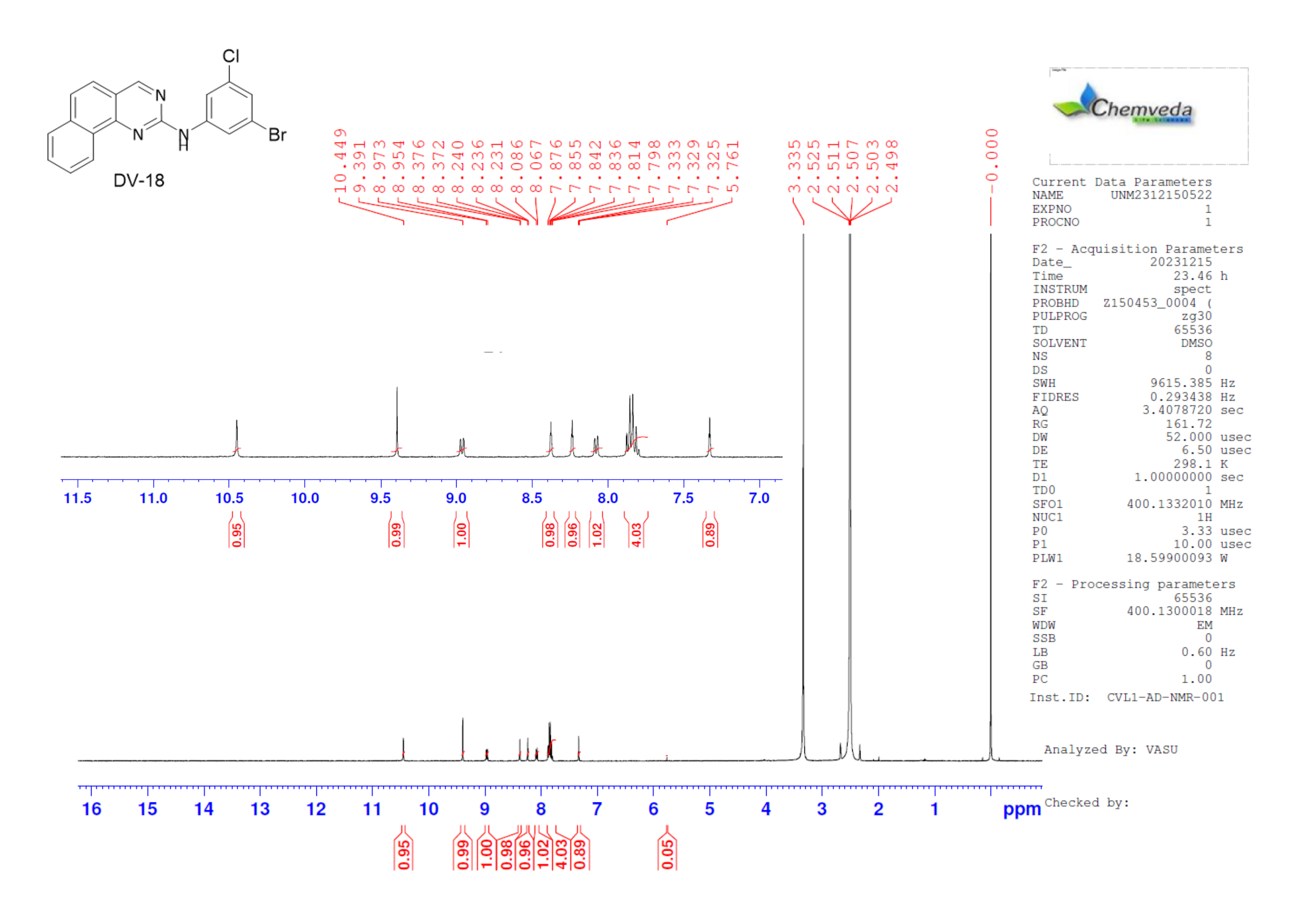

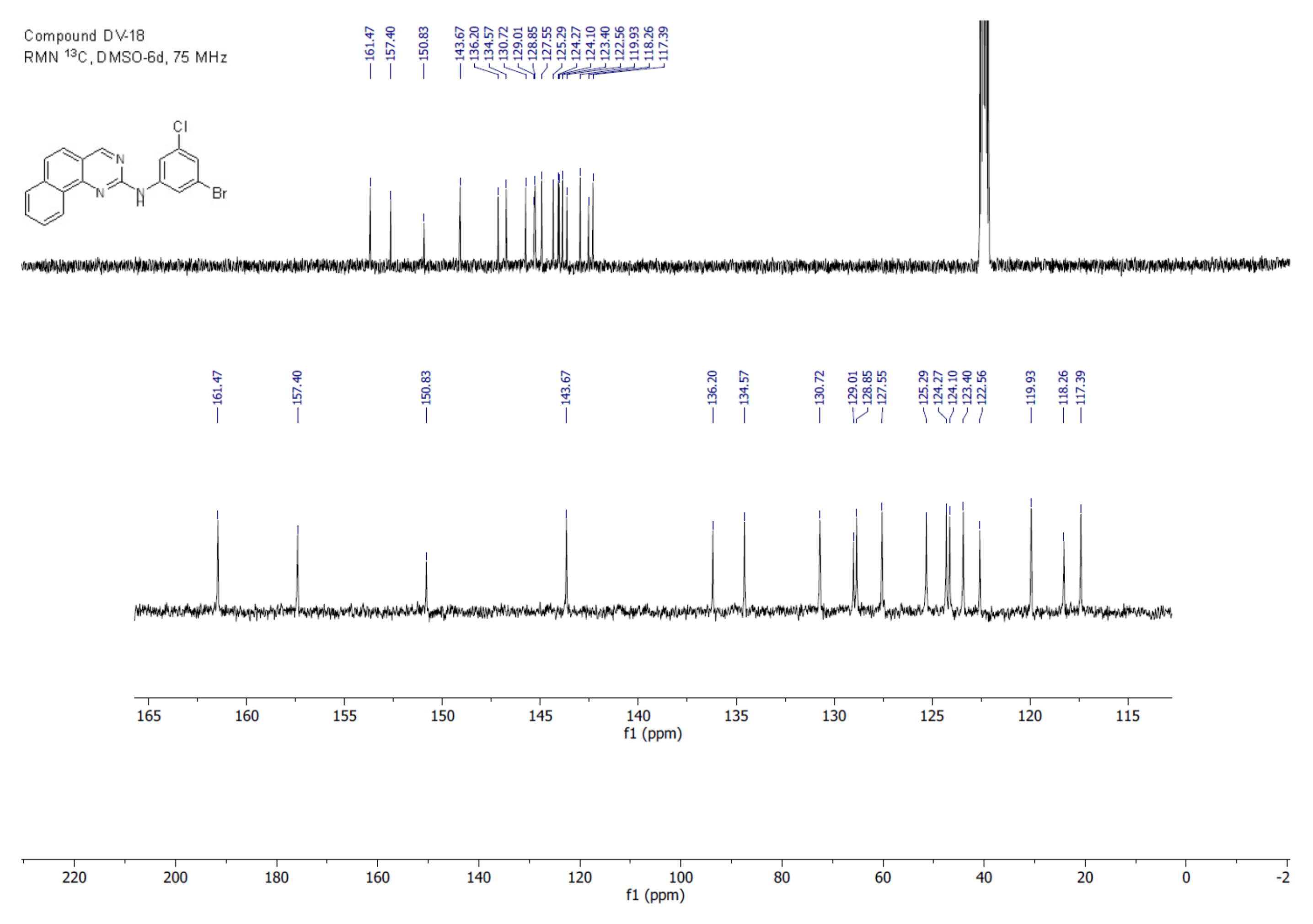

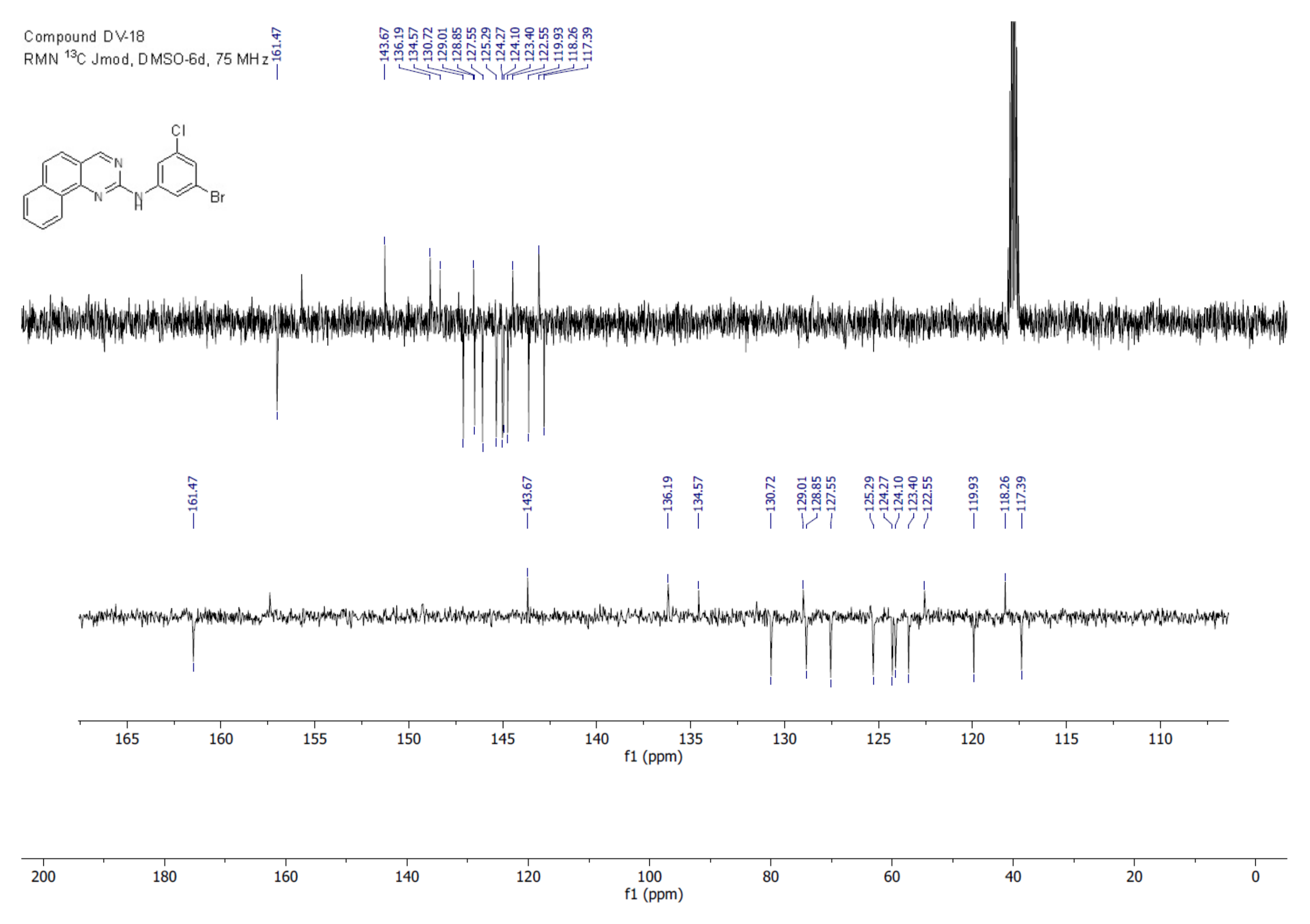
Compound DV-18
RMN ^{13}C Jmod, DMSO-6d, 75 MHz
161.47
143.67
136.19
134.57
130.72
129.01
128.85
127.55
125.29
124.27
124.10
123.40
122.55
119.93
118.26
117.39
Cl
N
N
N
H
Br
f1 (ppm)
f1 (ppm)

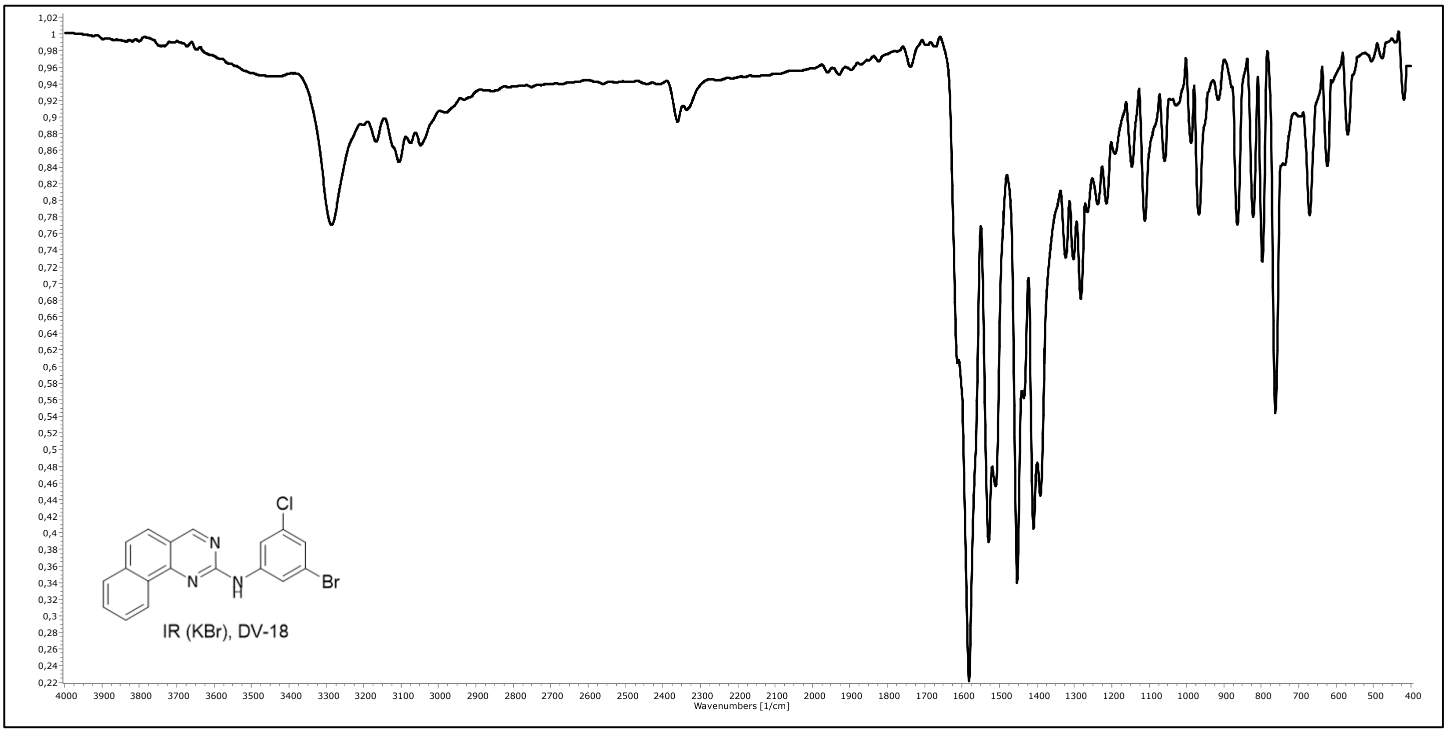
Cl
N
N
N
H
Br
IR (KBr), DV-18
Wavenumbers [1/cm]

Spectra: ^{1}H, ^{13}C, ^{13}C Jmod NMR and FT-IR data of compound 4s (DV-20)

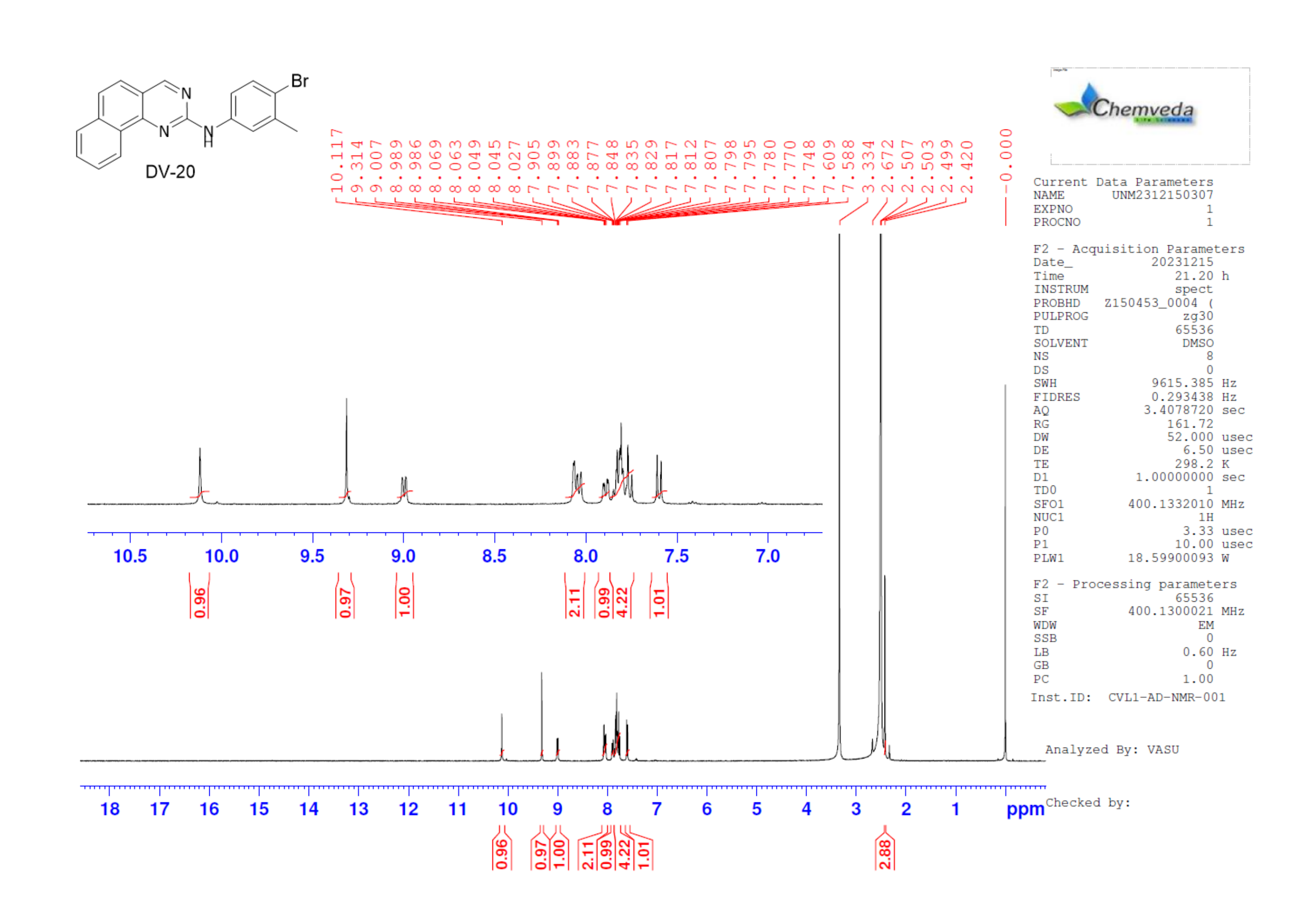

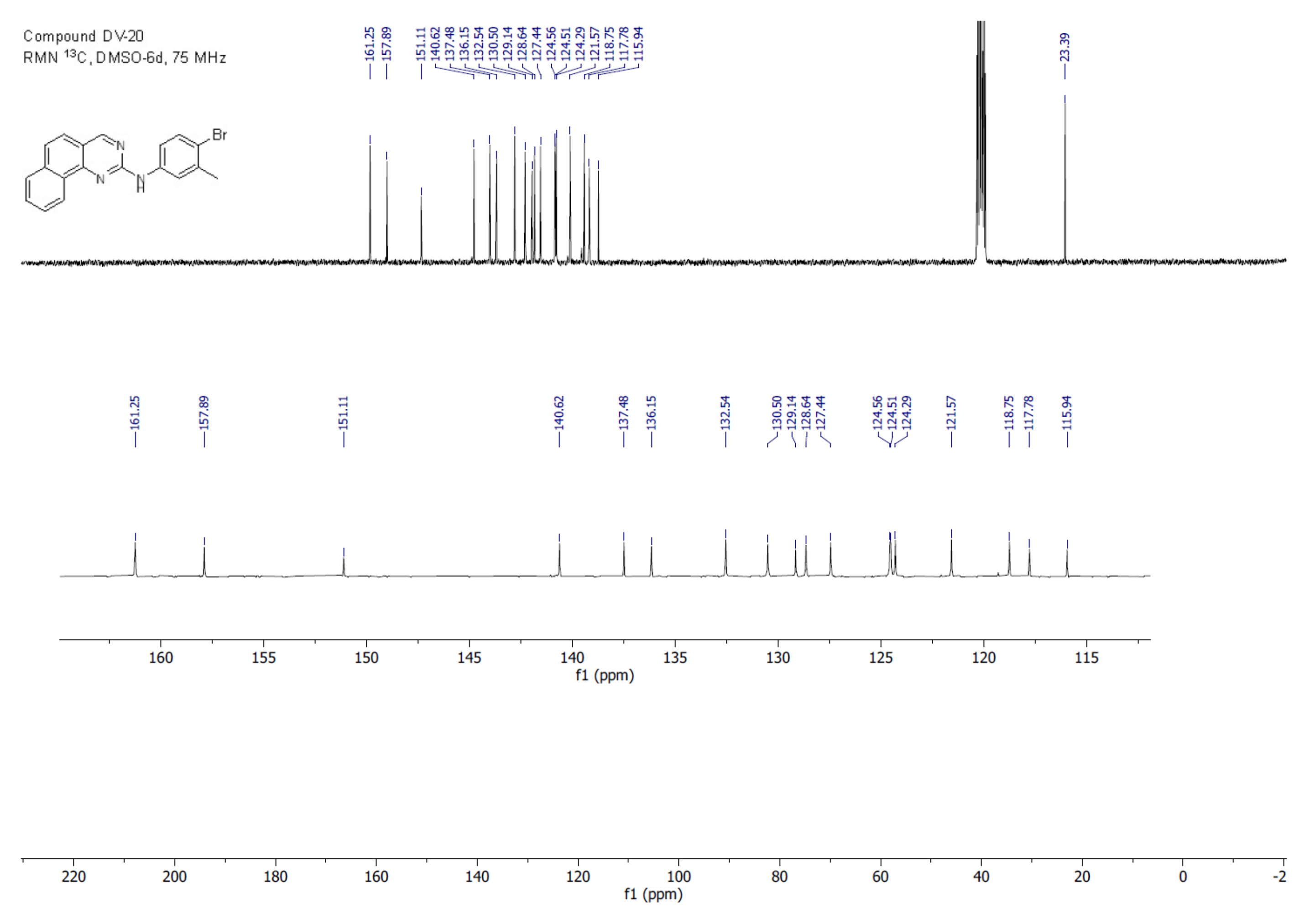

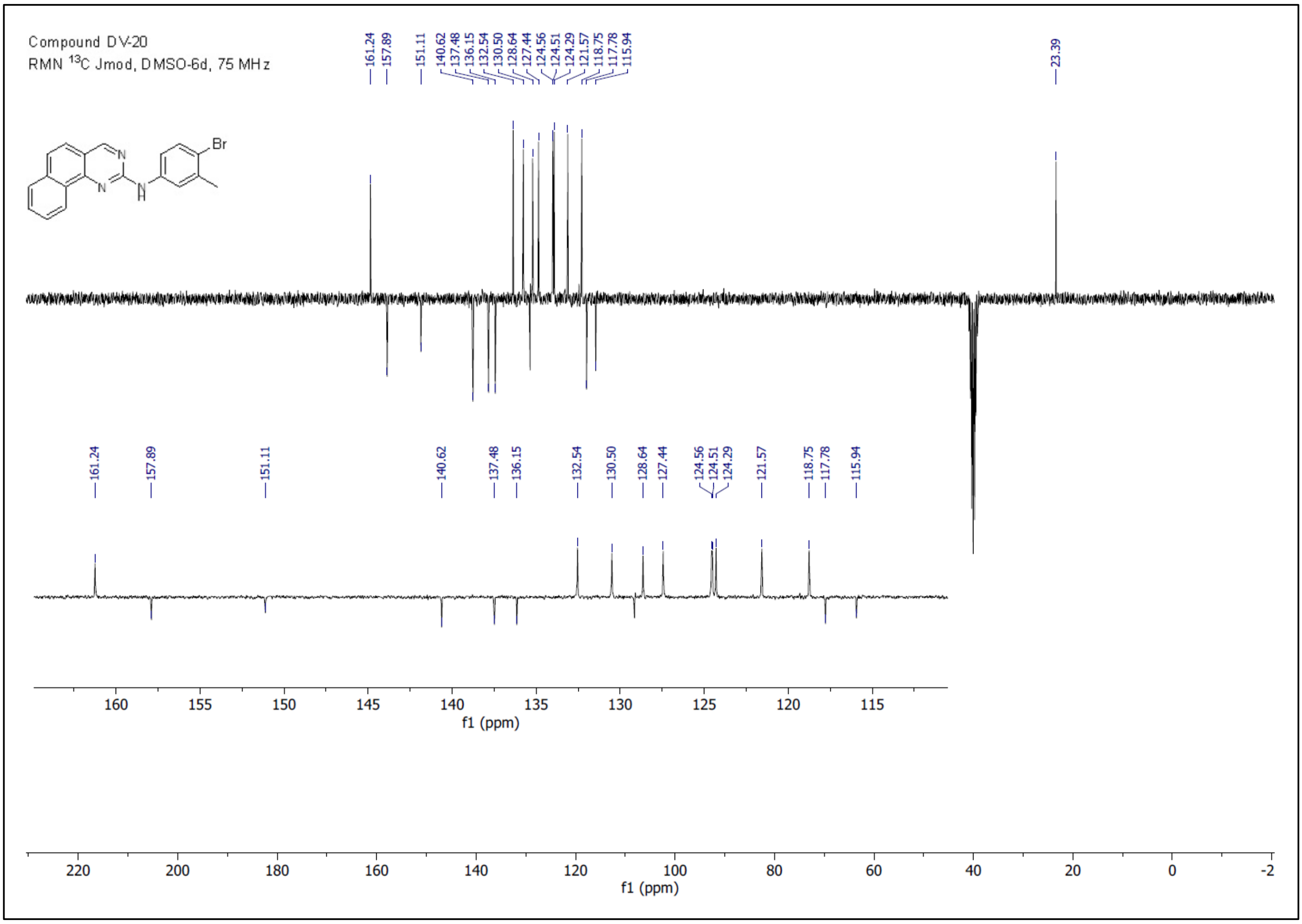
Compound DV-20
RMN ^{13}C Jmod, DMSO-6d, 75 MHz
161.24
157.89
151.11
140.62
137.48
136.15
132.54
130.50
128.64
127.44
124.56
124.51
124.29
121.57
118.75
117.78
115.94
23.39
Br
N
N
N
H
f1 (ppm)

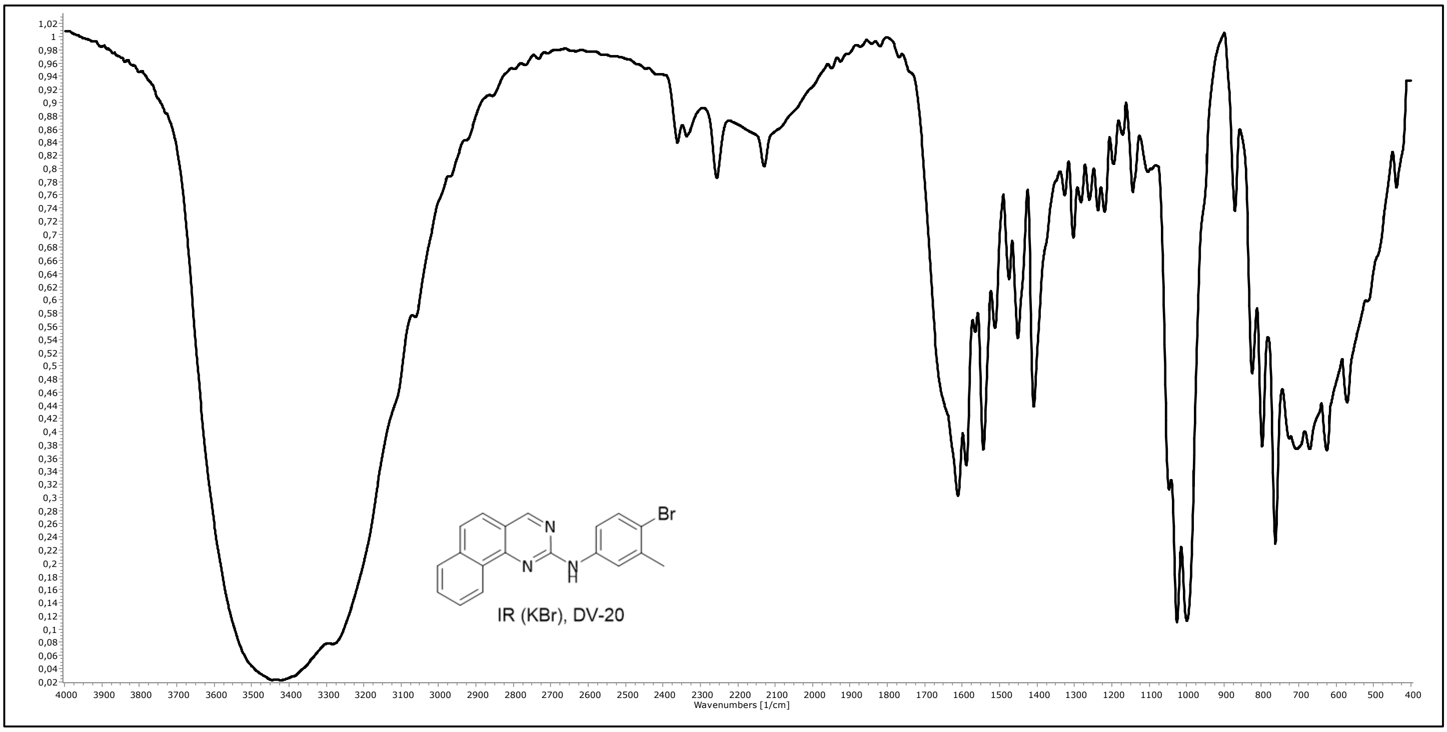
Br
N
N
N
H
IR (KBr), DV-20
Wavenumbers [1/cm]

Spectra: ^{1}H, ^{13}C, ^{13}C Jmod NMR and FT-IR data of compound 4r (DV-19)

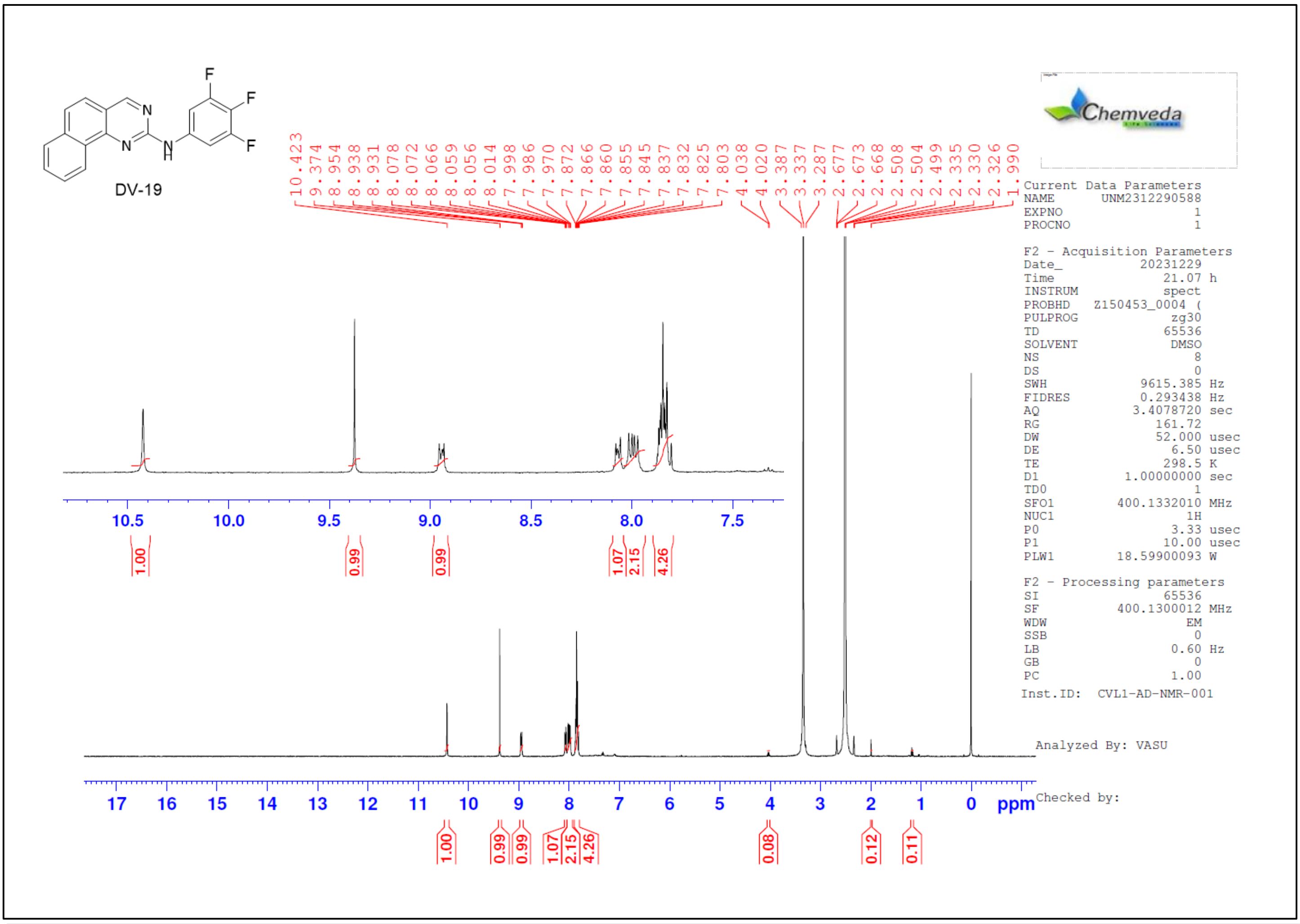

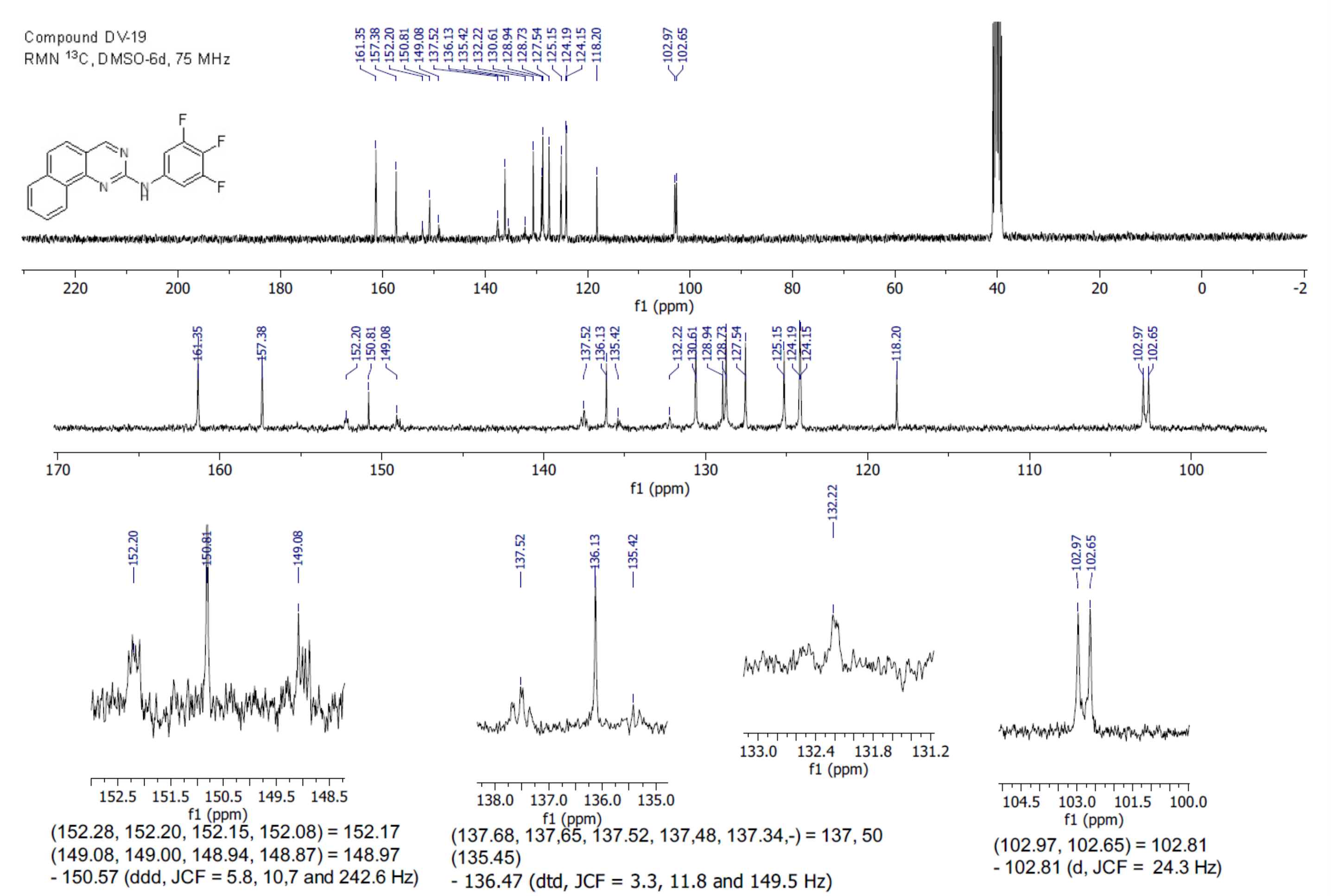

(152.28, 152.20, 152.15, 152.08) = 152.17
(149.08, 149.00, 148.94, 148.87) = 148.97
- 150.57 (ddd, JCF = 5.8, 10,7 and 242.6 Hz)

(137.68, 137,65, 137.52, 137,48, 137.34,-) = 137, 50
(135.45)
- 136.47 (dtd, JCF = 3.3, 11.8 and 149.5 Hz)

(102.97, 102.65) = 102.81
- 102.81 (d, JCF = 24.3 Hz)

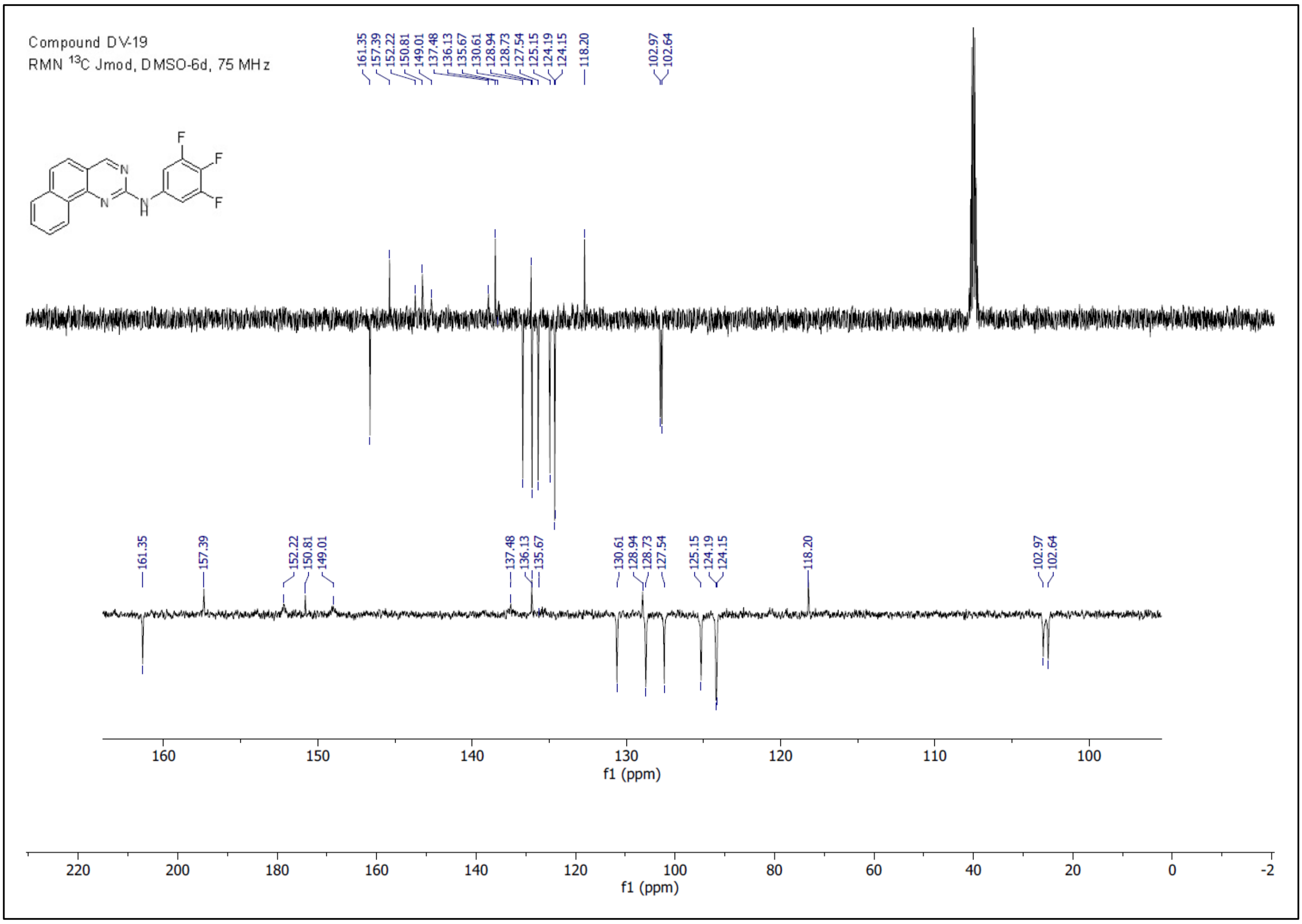
Compound DV-19
RMN ^{13}C Jmod, DMSO-6d, 75 MHz
161.35
157.39
152.22
150.81
149.01
137.48
136.13
135.67
130.61
128.94
128.73
127.54
125.15
124.19
124.15
118.20
102.97
102.64
f1 (ppm)

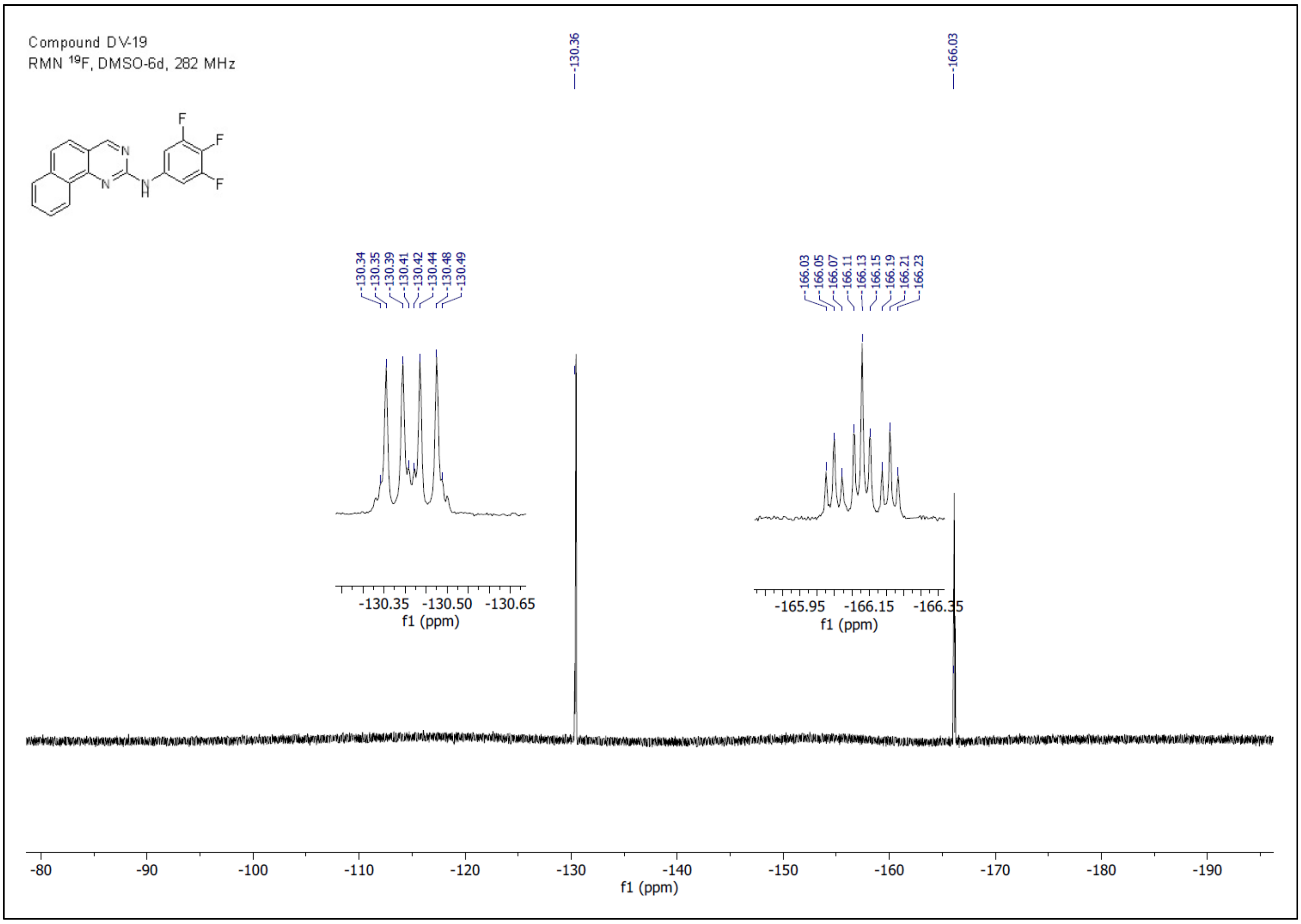
Compound DV-19
RMN ^{19}F, DMSO-6d, 282 MHz
-130.36
-166.03
-130.34
-130.35
-130.39
-130.41
-130.42
-130.44
-130.48
-130.49
-166.03
-166.05
-166.07
-166.11
-166.13
-166.15
-166.19
-166.21
-166.23
f1 (ppm)

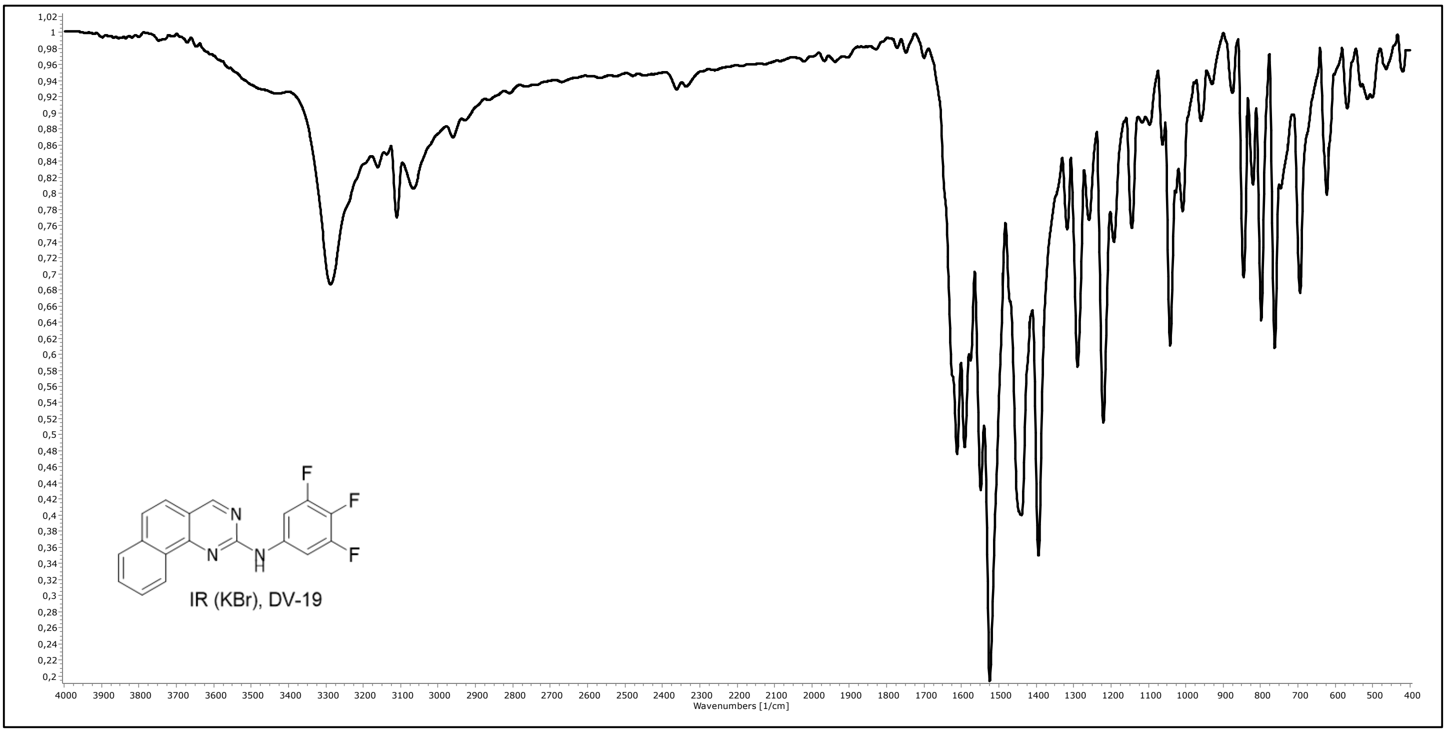

IR (KBr), DV-19
Wavenumbers [1/cm]
